# Supplementary material for: Genetic survey of biomarkers at early and mid-pregnancy identifies pregnancy-specialized immune regulation
Source: PLoS Genet. 2026 Jun 30;22(6):e1012204. doi: 10.1371/journal.pgen.1012204 (PMC13340790; doi:10.1371/journal.pgen.1012204)
Supplement: S1 Table — a. The table includes biomarkers where heritability estimate was significant in T1 but not in T2. Per biomarker, three rows are listed under Source column: V(G): estimated genetic variance, Vp: estimated phenotypic variance, V(G)/Vp: heritability estimate. SE columns list standard errors of these estimates. The left half of the table includes estimates from full T1 and T2 datasets. The right half with T1 Subset/T2 Subset includes estimates from repeated analyses with individuals that have both T1 and T2 measurements. Near-zero heritability estimates are shown in bold. b. Same as (a), with biomarkers where heritability estimate was significant in T2 but not in T1. (PDF) [file pgen.1012204.s001.pdf]

a

	Source	T1 Var	T1 SE	T2 Var	T2 SE		T1 Subset Var	T1 Subset SE	T2 Subset Var	T2 Subset SE
**TNFβ**	V(G)	1.56	0.47	0.46	0.47		1.85	0.57	0.87	0.55
	Vp	2.24	0.09	2.18	0.09		2.27	0.10	2.22	0.1
	V(G)/Vp	0.70	0.20	0.21	0.21		0.82	0.23	0.39	0.24
**IL-12p70**	V(G)	1.64	0.59	0.20	0.57		1.41	0.66	0.68	0.65
	Vp	3.14	0.12	3.15	0.12		3.15	0.13	3.22	0.13
	V(G)/Vp	0.52	0.18	0.062	0.18		0.45	0.20	0.21	0.2
**IL-22**	V(G)	0.57	0.31	0	0.3		0.67	0.37	0	0.32
	Vp	1.32	0.06	1.23	0.06		1.32	0.06	1.18	0.06
	V(G)/Vp	0.43	0.23	**0**	0.25		0.51	0.27	**0**	0.27
**IL-4**	V(G)	0.29	0.12	0.024	0.11		0.21	0.13	0.017	0.12
	Vp	0.82	0.03	0.82	0.03		0.82	0.03	0.83	0.03
	V(G)/Vp	0.35	0.14	**0.029**	0.14		0.26	0.16	**0.020**	0.15
**IL-17F**	V(G)	0.65	0.33	0	0.31		0.60	0.37	0	0.34
	Vp	2.22	0.07	2.20	0.07		2.21	0.08	2.22	0.08
	V(G)/Vp	0.29	0.14	**0**	0.14		0.27	0.16	**0**	0.15
**IL-12p40**	V(G)	0.12	0.06	0	0.07		0.11	0.07	0	0.08
	Vp	0.44	0.01	0.54	0.02		0.45	0.02	0.54	0.02
	V(G)/Vp	0.28	0.13	**0**	0.14		0.23	0.16	**0**	0.15

b

	Source	T1 Var	T1 SE	T2 Var	T2 SE		T1 Subset Var	T1 Subset SE	T2 Subset Var	T2 Subset SE
**MIP-1α**	V(G)	0	0.10	0.22	0.11		0	0.12	0.22	0.12
	Vp	0.71	0.02	0.69	0.02		0.72	0.03	0.69	0.03
	V(G)/Vp	**0**	0.14	0.32	0.15		**0**	0.16	0.32	0.17
**IL-1RA**	V(G)	0.029	0.06	0.13	0.07		0.10	0.08	0.14	0.08
	Vp	0.50	0.02	0.51	0.02		0.50	0.02	0.51	0.02
	V(G)/Vp	0.058	0.13	0.25	0.14		0.20	0.15	0.26	0.15

S1 Table. SNP-based heritability estimates for full cohort and T1/T2 subset. a. The table includes biomarkers where heritability estimate was significant in T1 but not in T2. Per biomarker, three rows are listed under Source column: V(G): estimated genetic variance, Vp: estimated phenotypic variance, V(G)/Vp: heritability estimate. SE columns list standard errors of these estimates. The left half of the table includes estimates from full T1 and T2 datasets. The right half with T1 Subset/T2 Subset includes estimates from repeated analyses with individuals that have both T1 and T2 measurements. Near-zero heritability estimates are shown in bold. **b.** Same as (a), with biomarkers where heritability estimate was significant in T2 but not in T1.
