# Supplementary material for: Genetic survey of biomarkers at early and mid-pregnancy identifies pregnancy-specialized immune regulation
Source: PLoS Genet. 2026 Jun 30;22(6):e1012204. doi: 10.1371/journal.pgen.1012204 (PMC13340790; doi:10.1371/journal.pgen.1012204)
Supplement: S2 Table — If there are multiple SNPs annotated to the same gene, only the most significant SNP is displayed. (PDF) [file pgen.1012204.s002.pdf]

Biomarker	CHR	Gene (TF)	SNP	Δ *P*
IL-1β	7q21.3	*BHLHA15*	rs73151168	6.16×10^{-7}
GM-CSF	2p21	*EPAS1*	rs79824099	8.07×10^{-7}
IL-13	7p22.1	*FOXK1*	rs78685992	7.93×10^{-7}
TGFα	7p22.2	*FOXK1*	rs28609201	2.99×10^{-7}
IL-1α	6p25.3	*FOXQ1*	rs114247784	1.73×10^{-7}
IL-15	3q29	*HES1*	rs4686663	1.92×10^{-7}
GM-CSF	16q23.1	*MAF*	rs12444900	4.89×10^{-7}
TNFβ	5q14.3	*MEF2C*	rs145965369	4.76×10^{-7}
Eotaxin	8q13.1	*MYBL1*	rs547031641	8.46×10^{-7}
IP-10	14q13.3	*PAX9*	rs4904582	1.48×10^{-8}
MIP-1α	15q22.2	*RORA*	rs1370432	1.49×10^{-7}
IFNγ	6p21.1	*RUNX2*	rs78548485	1.74×10^{-7}
IL-12p70	9q34.2	*RXRA*	rs3132301	2.65×10^{-7}
M-CSF	18q23	*SALL3*	rs34653042	7.63×10^{-7}
RANTES	18q23	*SALL3*	rs878916908	5.01×10^{-7}
M-CSF	2p25.2	*SOX11*	rs73913776	8.67×10^{-7}
IL-12p40	2p25.2	*SOX11*	rs1389491716	3.25×10^{-7}
FGF-2	12p12.1	*SOX5*	rs544998569	4.16×10^{-7}
FLT-3L	22q13.31	*TBC1D22A*	rs16996074	5.34×10^{-7}
TNFα	15q23	*TLE3*	rs11072135	8.63×10^{-7}
IL-1α	10q24.31	*TLX1*	rs10128244	5.11×10^{-8}
IL-2	19q12	*URI1*	rs75515131	1.65×10^{-7}
IL-6	17q22	*VEZF1*	rs62081792	3.06×10^{-7}
IL-1α	2q22.3	*ZEB2*	rs74468904	6.17×10^{-7}

S2 Table. Δ-associated SNPs ($P<10^{-6}$) annotated to genes encoding transcription factors. If there are multiple SNPs annotated to the same gene, only the most significant SNP is displayed.
