# Supplementary material for: Genetic survey of biomarkers at early and mid-pregnancy identifies pregnancy-specialized immune regulation
Source: PLoS Genet. 2026 Jun 30;22(6):e1012204. doi: 10.1371/journal.pgen.1012204 (PMC13340790; doi:10.1371/journal.pgen.1012204)
Supplement: S1 Fig — a-c. Pairwise Pearson correlation matrices of T1 (a), T2 (b), and Δ (c). T1 heatmap (red - positive correlation; blue – negative correlation) is ordered based on hierarchical clustering, and biomarkers in T2 and Δ heatmaps are presented in the same order for comparison. The yellow squares highlight 11 highly correlated biomarkers, the purple squares highlight sCD40L-EGF-IL-7. d-f. Pairwise correlation matrices of T1 & T2 (d), T1 & Δ (e), and T2 & Δ (f). T1 labels are shown in black, T2 in green, and Δ in purple. (PDF) [file pgen.1012204.s003.pdf]

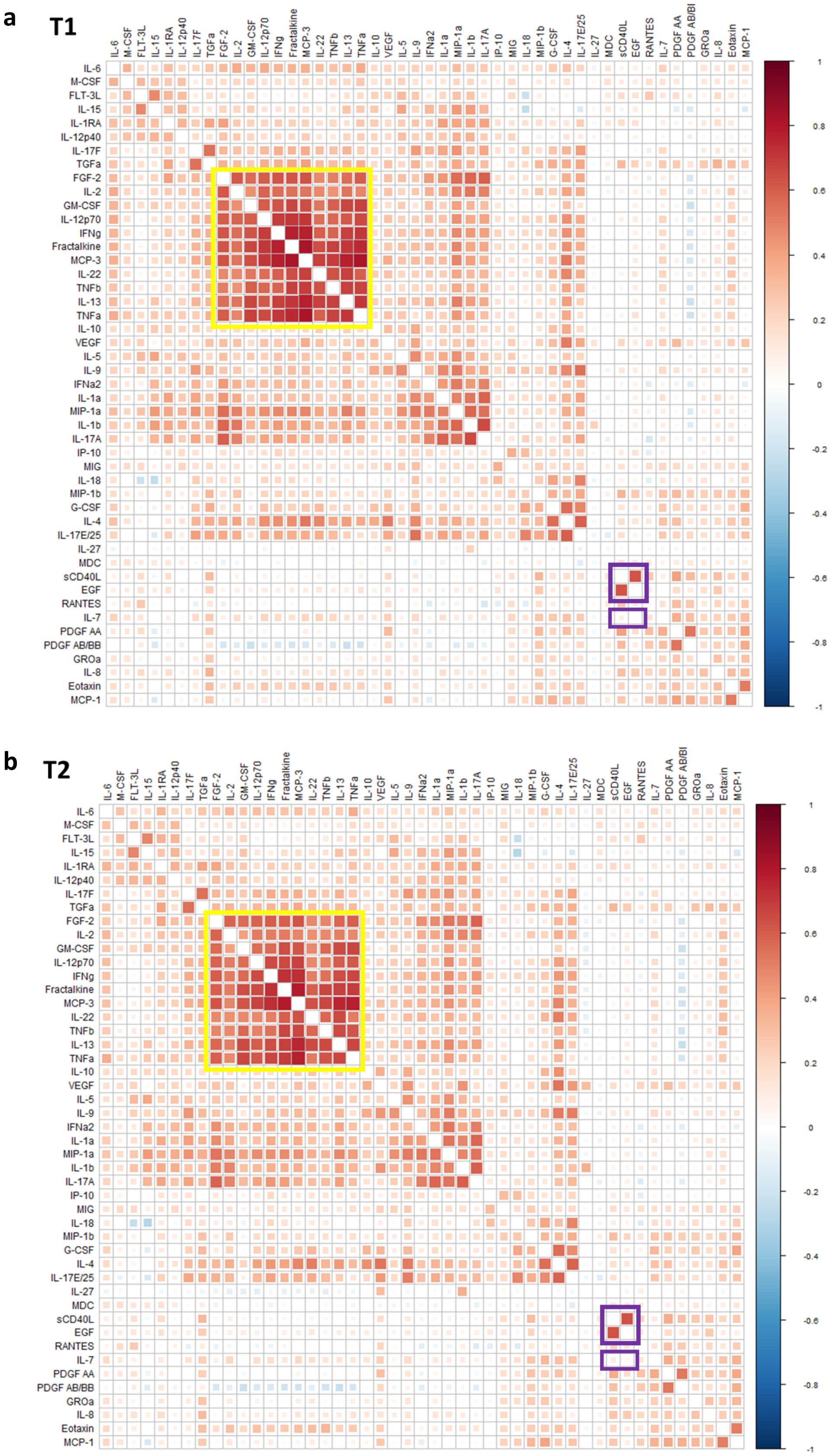
a
T1
IL-6
M-CSF
FLT-3L
IL-15
IL-1RA
IL-12p40
IL-17F
TGFa
FGF-2
IL-2
GM-CSF
IL-12p70
IFNg
Fractalkine
MCP-3
IL-22
TNFb
IL-13
TNFa
IL-10
VEGF
IL-5
IL-9
IFNa2
IL-1a
MIP-1a
IL-1b
IL-17A
IP-10
MIG
IL-18
MIP-1b
G-CSF
IL-4
IL-17E/25
IL-27
MDC
sCD40L
EGF
RANTES
IL-7
PDGF AA
PDGF AB/BB
GROa
IL-8
Eotaxin
MCP-1
1
0.8
0.6
0.4
0.2
0
-0.2
-0.4
-0.6
-0.8
-1
b
T2

c **Δ**

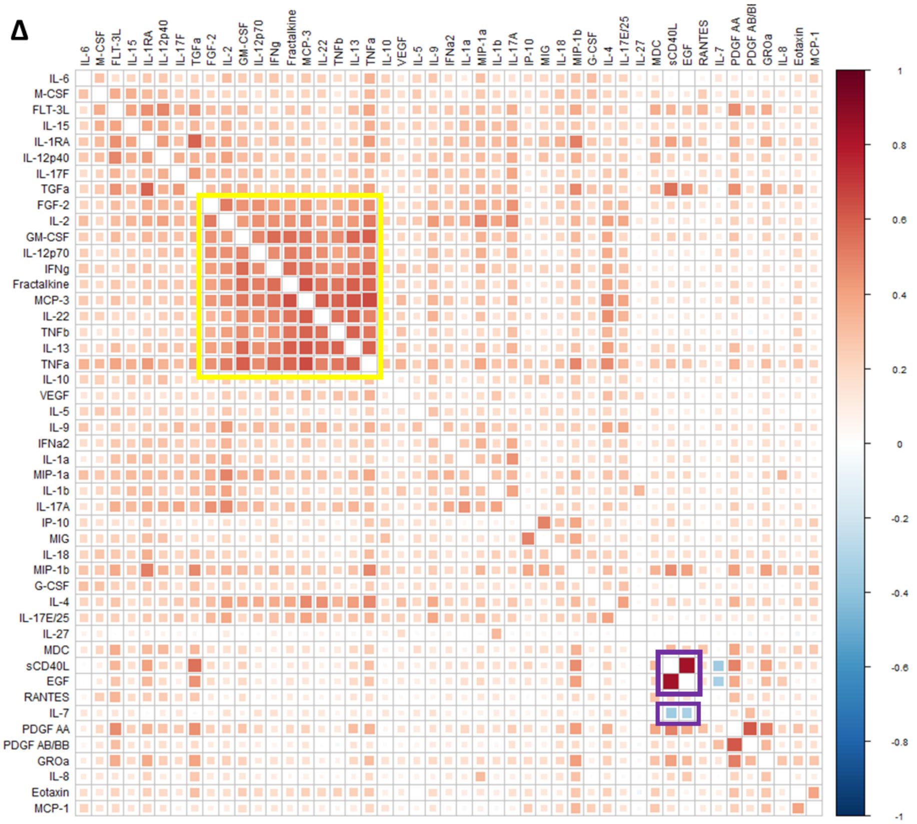
IL-6
M-CSF
FLT-3L
IL-15
IL-1RA
IL-12p40
IL-17F
TGFa
FGF-2
IL-2
GM-CSF
IL-12p70
IFNg
Fractalkine
MCP-3
IL-22
TNFb
IL-13
TNFa
IL-10
VEGF
IL-5
IL-9
IFNa2
IL-1a
MIP-1a
IL-1b
IL-17A
IP-10
MIG
IL-18
MIP-1b
G-CSF
IL-4
IL-17E/25
IL-27
MDC
sCD40L
EGF
RANTES
IL-7
PDGF AA
PDGF AB/BB
GROa
IL-8
Eotaxin
MCP-1
1
0.8
0.6
0.4
0.2
0
-0.2
-0.4
-0.6
-0.8
-1

d **T1 & T2**

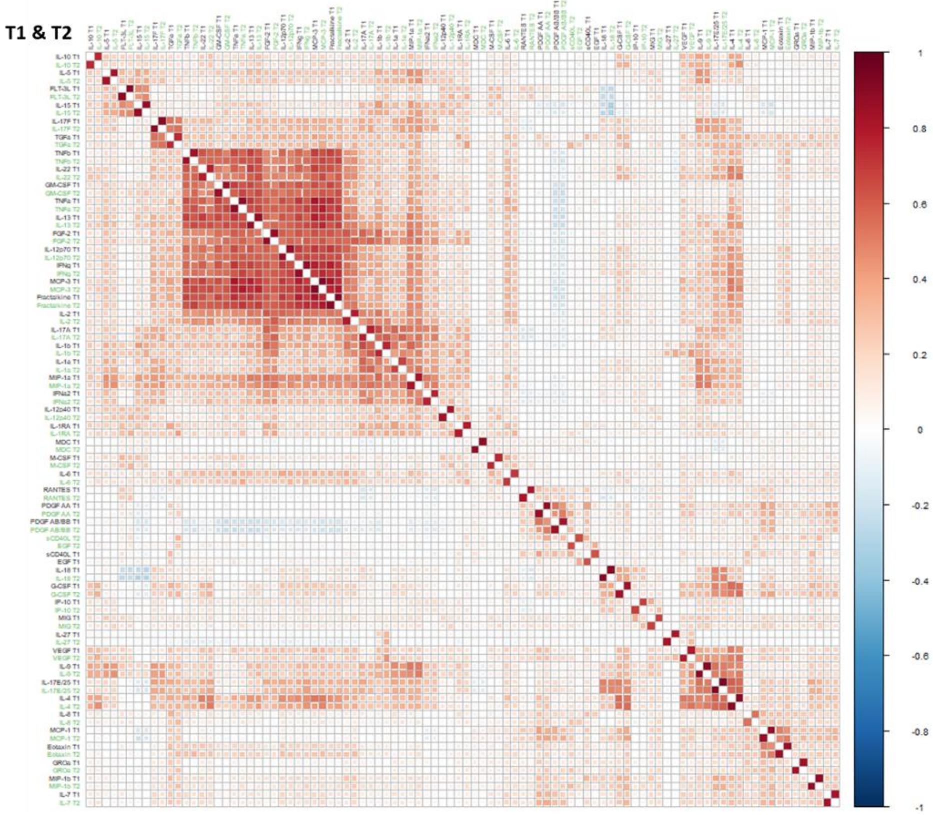
1
0.8
0.6
0.4
0.2
0
-0.2
-0.4
-0.6
-0.8
-1

e

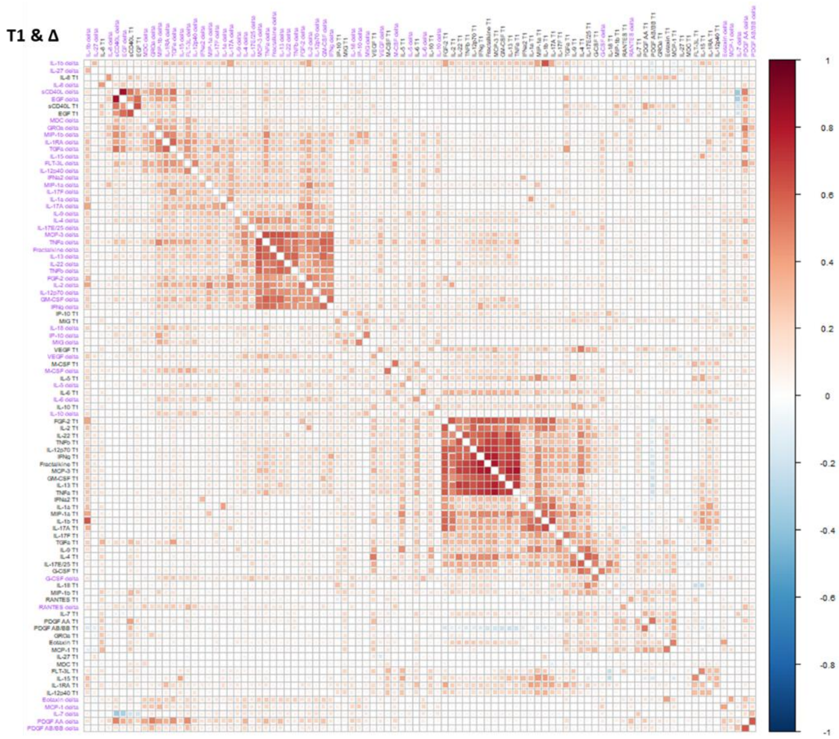

f

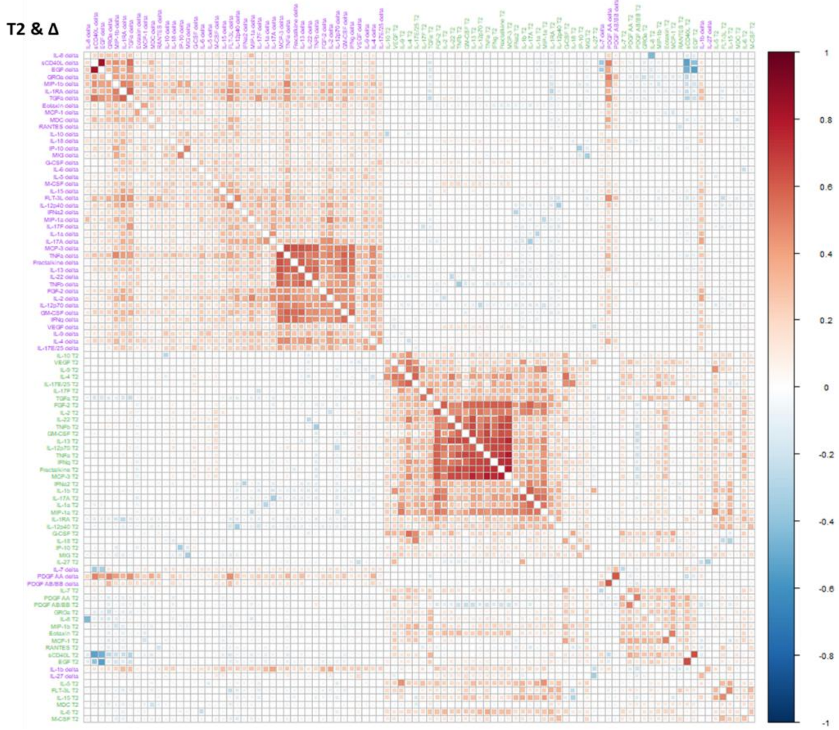

S1 Fig. Biomarker correlation. a-c. Pairwise Pearson correlation matrices of T1 (a), T2 (b), and Δ (c). T1 heatmap (red - positive correlation; blue – negative correlation) is ordered based on hierarchical clustering, and biomarkers in T2 and Δ heatmaps are presented in the same order for comparison. The yellow squares highlight 11 highly correlated biomarkers, the purple squares highlight sCD40L-EGF-IL-7. **d-f.** Pairwise correlation matrices of T1 & T2 (d), T1 & Δ (e), and T2 & Δ (f). T1 labels are shown in black, T2 in green, and Δ in purple.
