# Supplementary figures and images for: Genetic survey of biomarkers at early and mid-pregnancy identifies pregnancy-specialized immune regulation
Source: PLoS Genet. 2026 Jun 30;22(6):e1012204. doi: 10.1371/journal.pgen.1012204 (PMC13340790; doi:10.1371/journal.pgen.1012204)

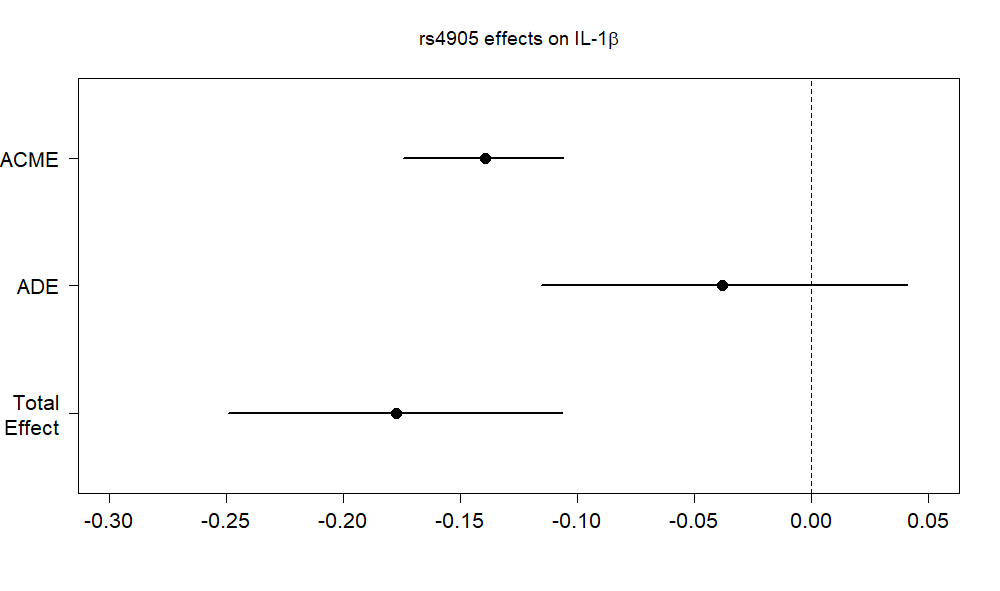

Supplement: S2 Fig — Average causal mediation effect (ACME) corresponds to indirect effect mediated through IL-27 (estimate: -0.14, 95% CI: -0.17, -0.11). Average direct effect (ADE) corresponds to rs4905’s direct effect on IL-1β (estimate: -0.038, 95% CI: -0.12, 0.04). Total effect is the combined effect of the two (estimate: -0.18, 95% CI: -0.25, -0.11). Proportion of mediated effects is 0.78. (PNG) [file pgen.1012204.s004.png]

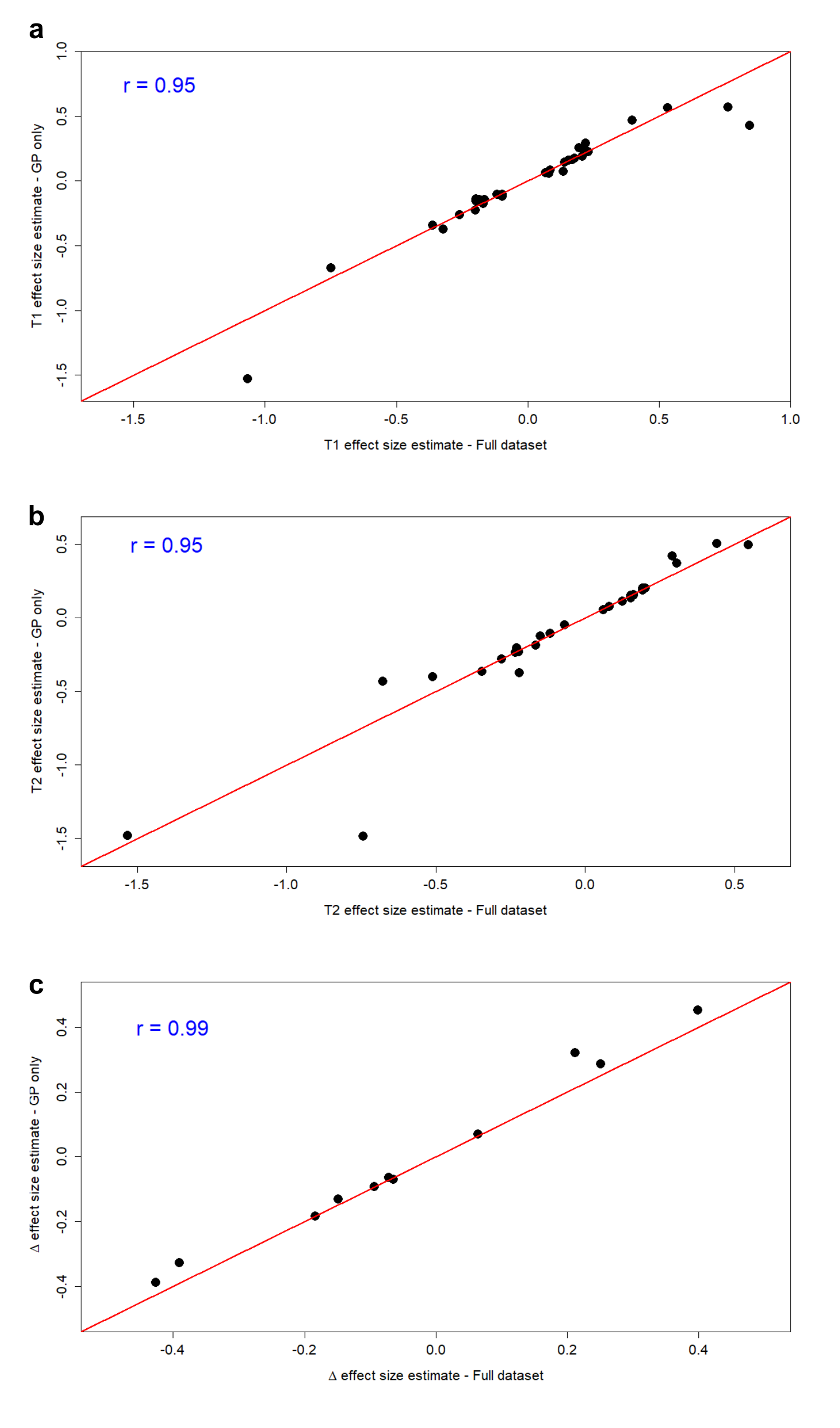

Supplement: S3 Fig — Effect size estimates of T1 (a), T2 (b), and Δ (c) GWS SNPs are plotted against estimates obtained when GWAS is repeated with GP-only samples, excluding pregnancies with ASD or DD outcomes. Correlation coefficients are displayed on the plots. (PNG) [file pgen.1012204.s005.png]

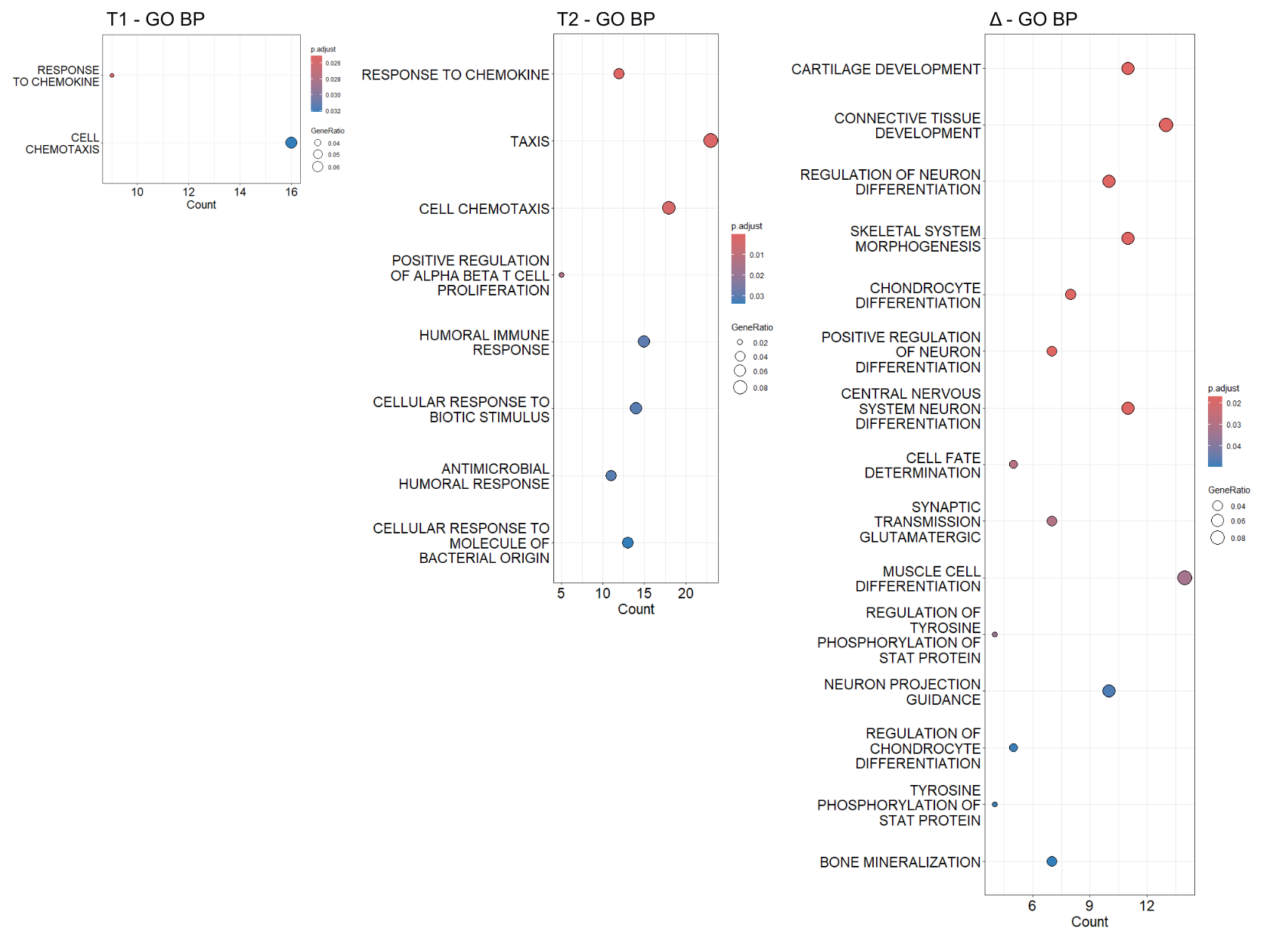

Supplement: S4 Fig — Significantly enriched GO-Biological Process pathways within each time variable’s set of query genes assigned to nearby suggestive SNPs (P < 10-6). Categories are ordered based on adjusted p-values and circles are color coded based on adjusted p-values. Count in x-axis denotes the number of genes in the query set that belong to the category. Circle sizes are proportional to gene ratio, which denotes the ratio of the number of genes in the query set in that category to the total number of genes in the category. (PNG) [file pgen.1012204.s006.png]

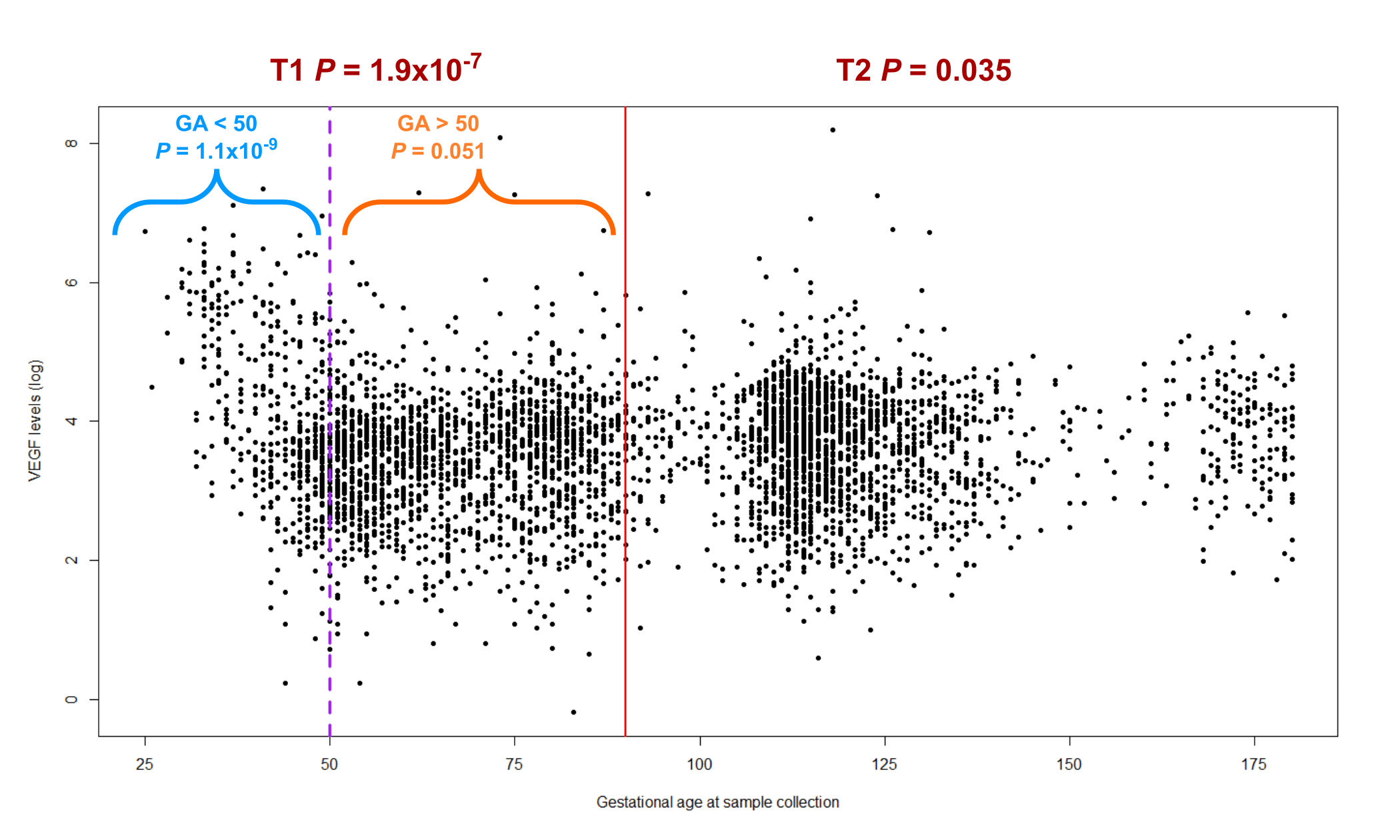

Supplement: S5 Fig — Log-normalized VEGF level from each sample is plotted against the corresponding gestational age. Red line indicates 90 days, the threshold between first and second trimesters. Dashed purple line indicates 50 days. T1 data were divided based on this 50-day timepoint and p-values of the association between rs6921438 and VEGF levels within each group are shown in blue and orange. The p-values of the association between rs6921438 and VEGF levels within full T1 and T2 datasets are shown in red. (PNG) [file pgen.1012204.s007.png]

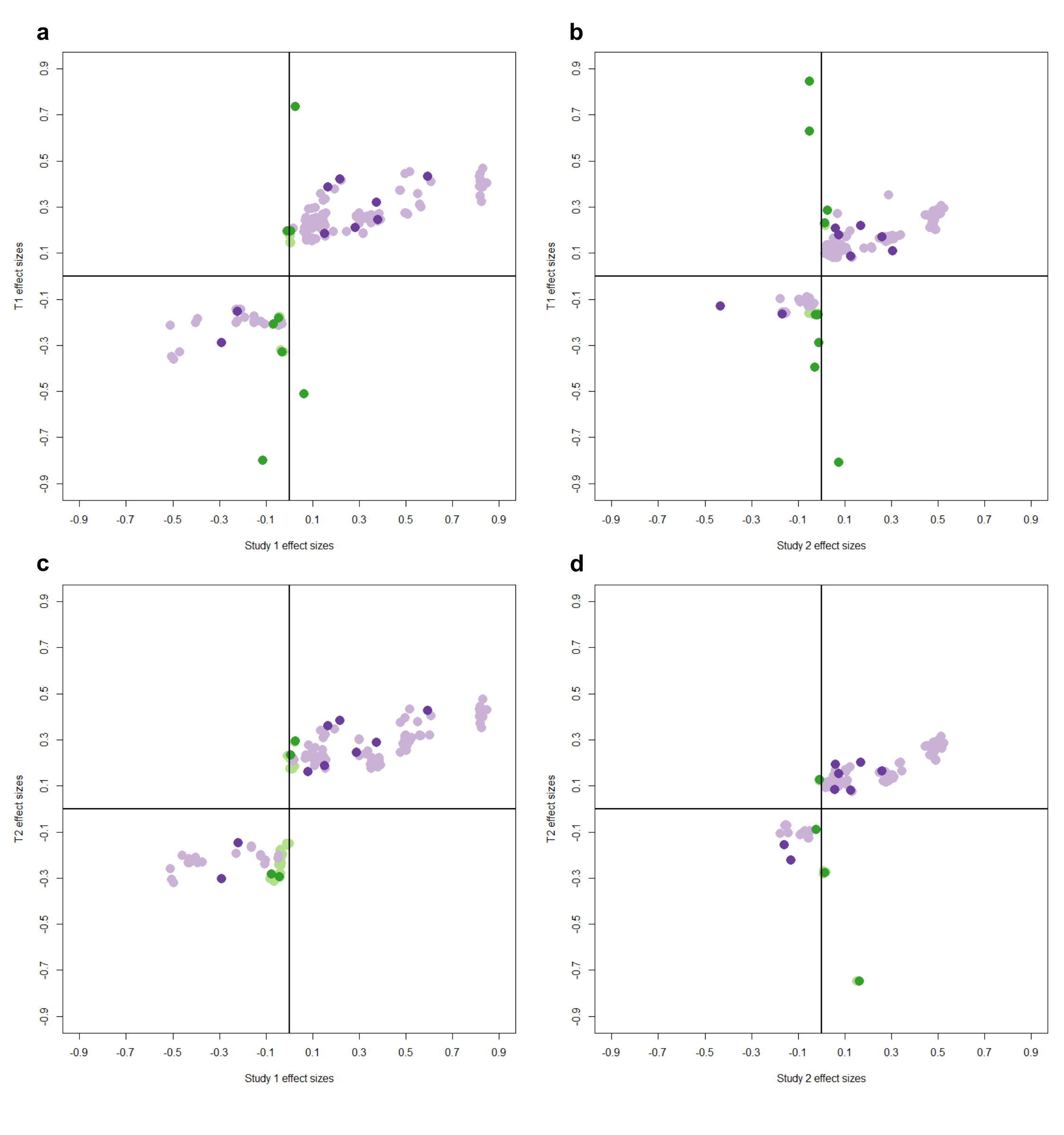

Supplement: S6 Fig — Each SNP shown in Fig 3 is included here where x-axis shows the effect size estimates of the comparison study and y-axis shows the effect size estimates of T1/T2. Purple dots correspond to shared SNPs and green dots correspond to unique SNPs. Darker shades represent the most significant index SNP of each independent genomic region. Study 1 comparisons (a and c) show estimates in SD units. a. T1-associated loci compared against study 1 b. T1-associated loci compared against study 2 c. T2-associated loci compared against study 1 d. T2-associated loci compared against study 2. (PNG) [file pgen.1012204.s008.png]

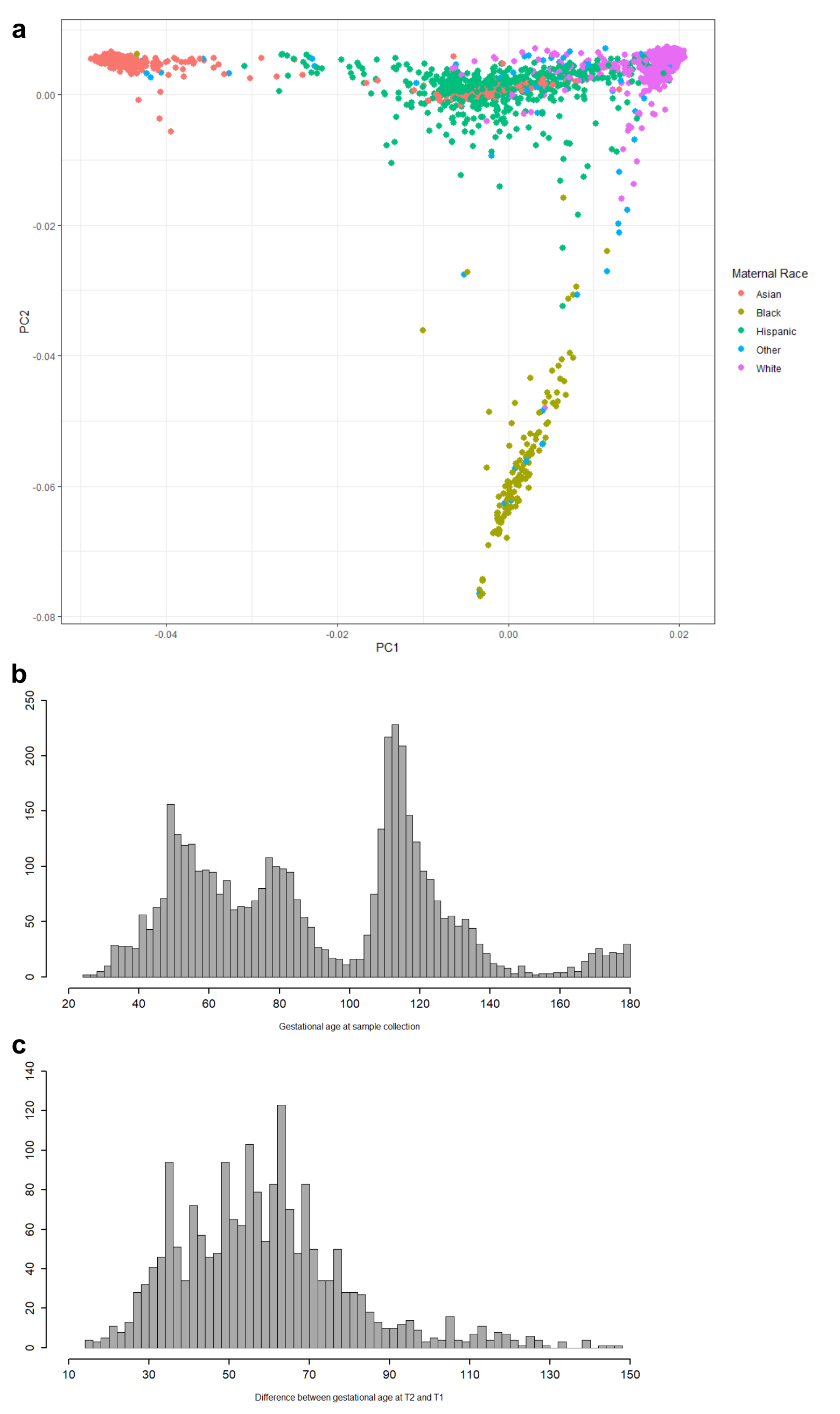

Supplement: S7 Fig — a. Genetic principal components PC1 and PC2 are plotted to visualize genetic ancestry. Dots are colored based on self-reported maternal race/ethnicity. b. Histogram of gestational age in days at sample collection. c. Histogram of differences in gestational age between first and second measurement collection. (PNG) [file pgen.1012204.s009.png]

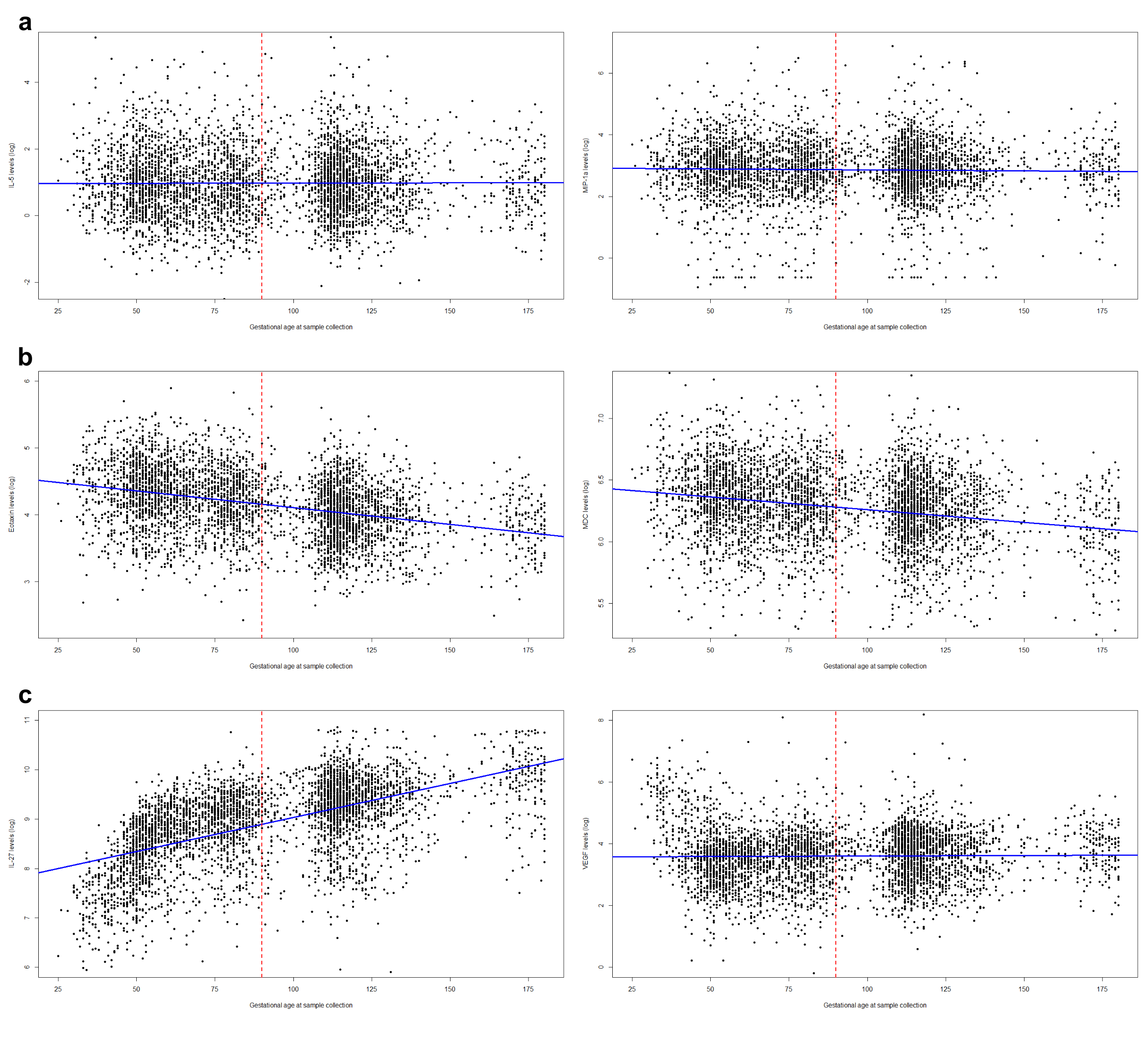

Supplement: S8 Fig — Log-normalized immune biomarker levels from each sample are plotted against the corresponding gestational age at sample collection. Dashed red line indicates 90 days, the threshold between first and second trimesters. A subset of biomarkers is selected to represent biomarkers with stable levels across time (a), biomarkers with linear relationship with gestational age (decreasing over time) (b), and biomarkers with non-linear relationship with gestational age (c). The fitted linear line is shown in blue to highlight these different trends. (PNG) [file pgen.1012204.s010.png]

**a**

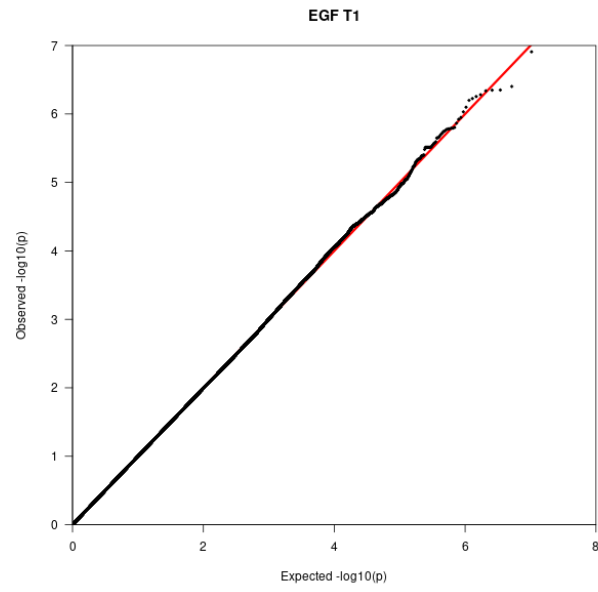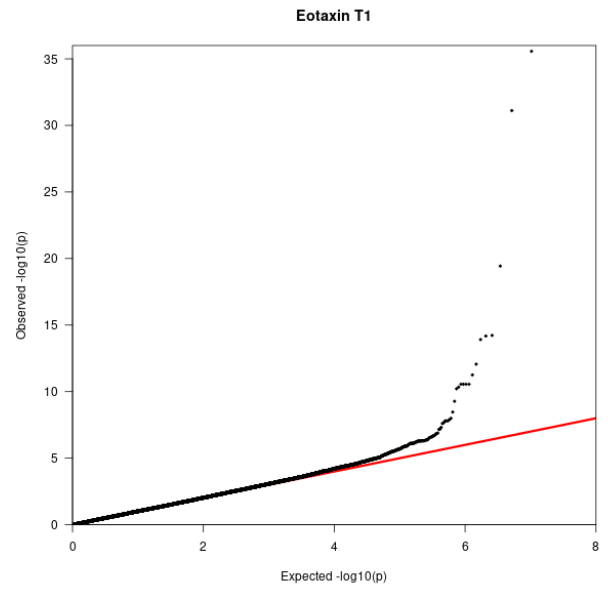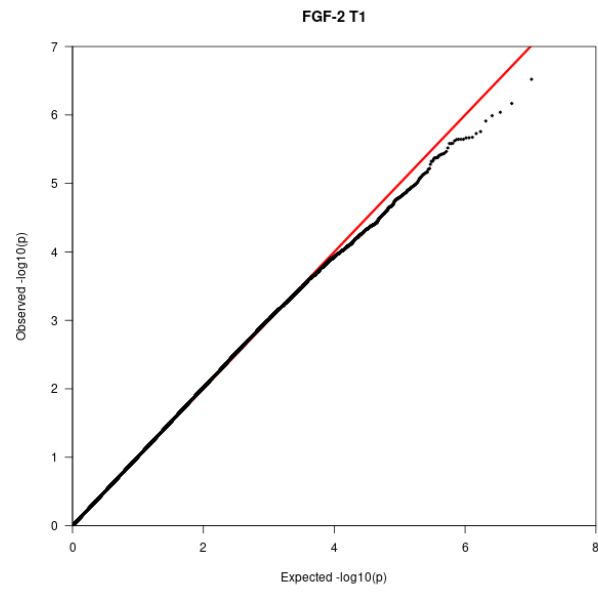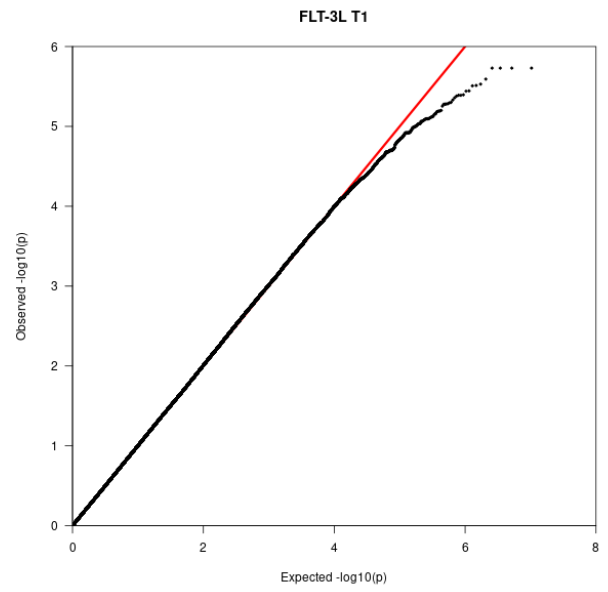

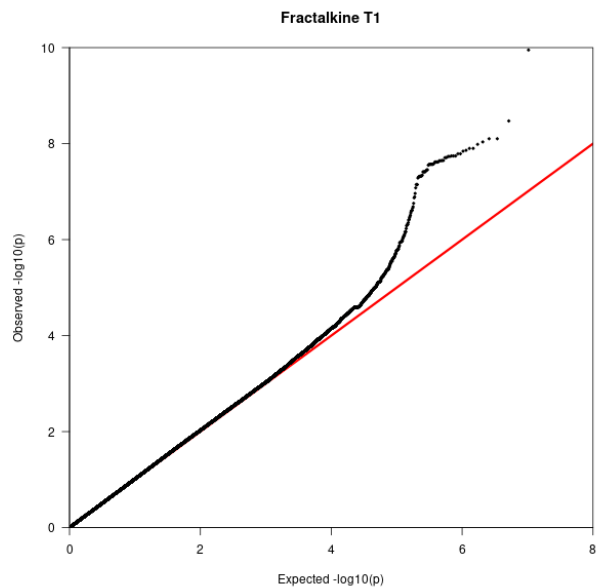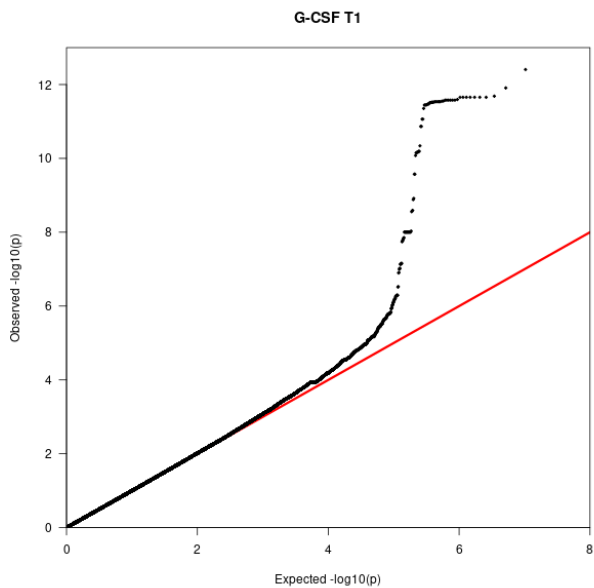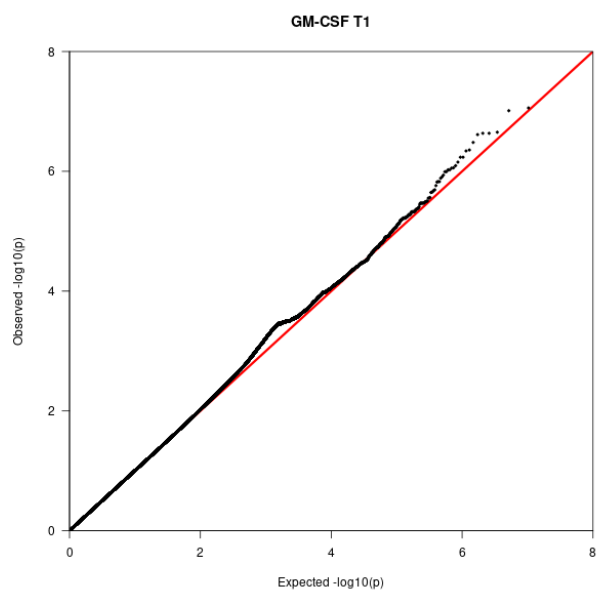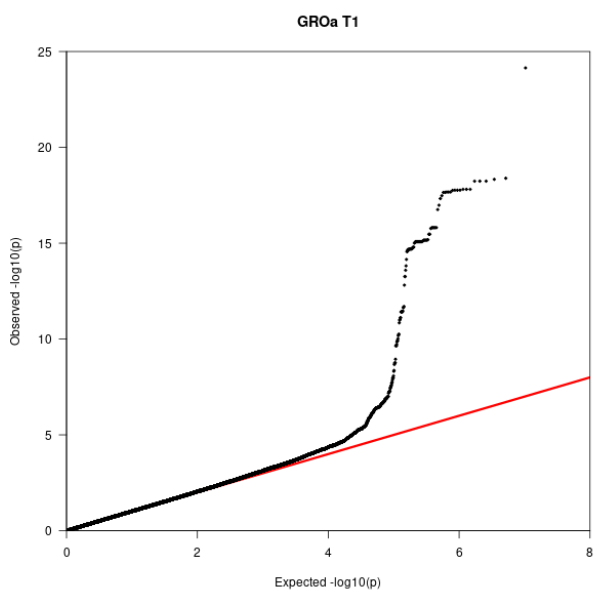

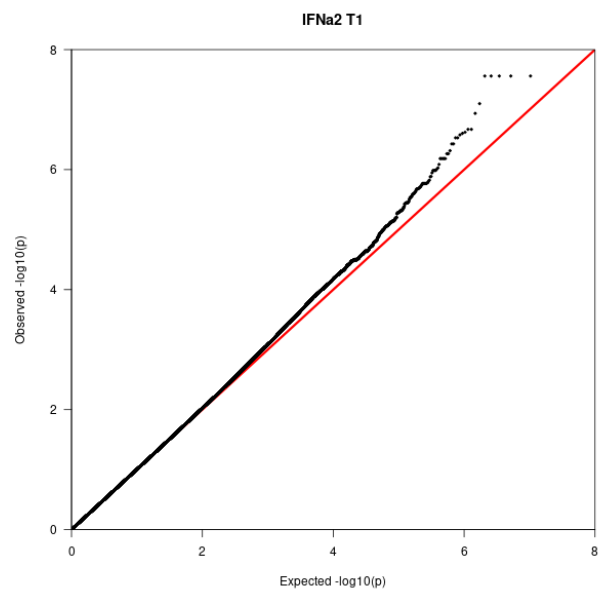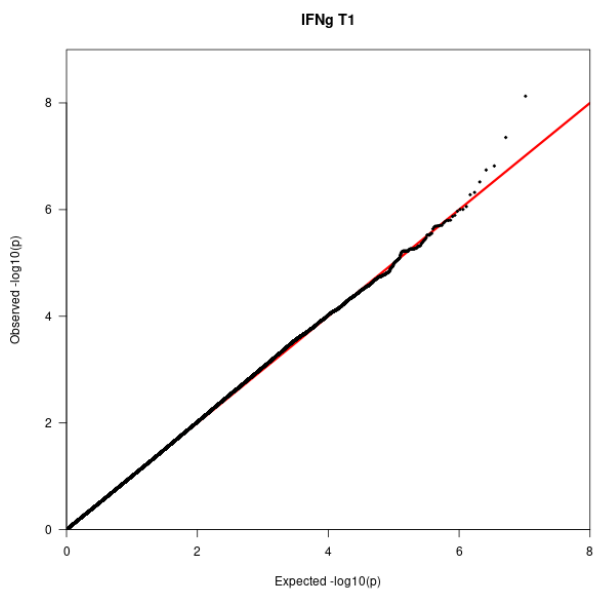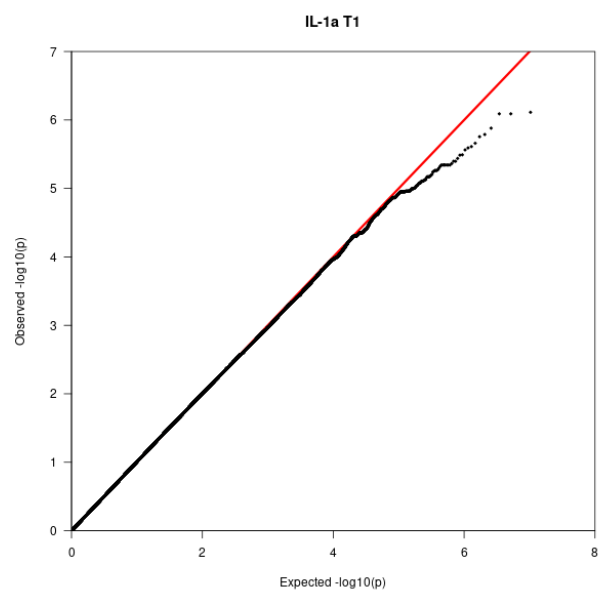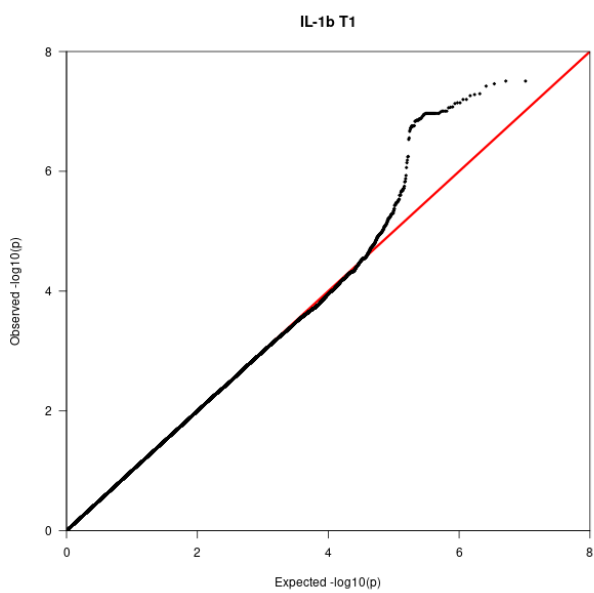

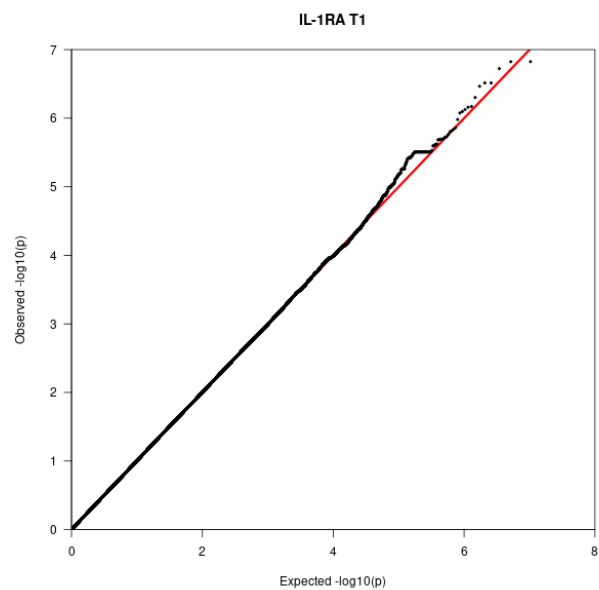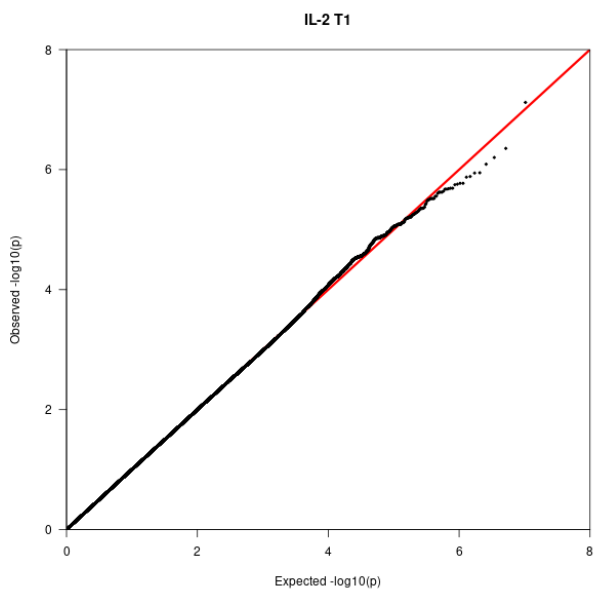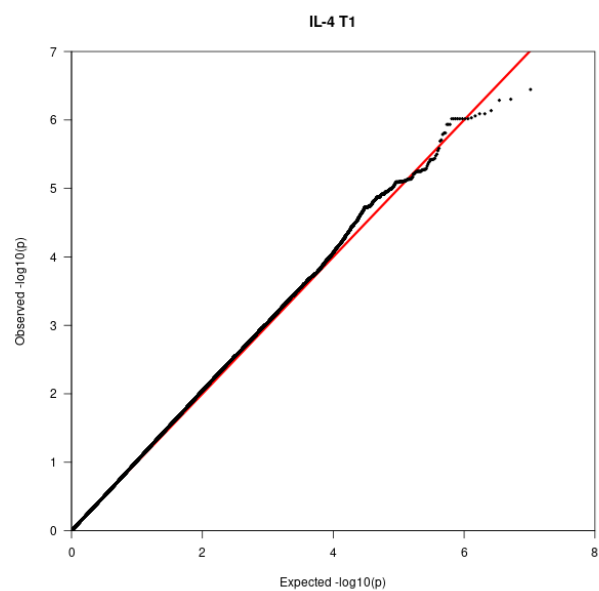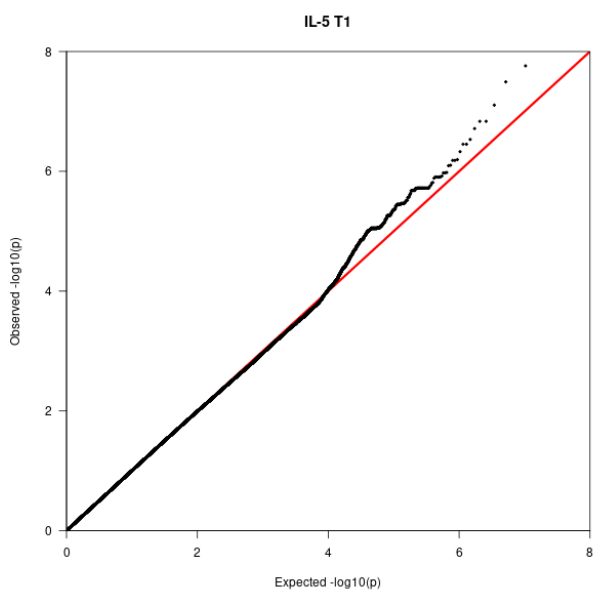

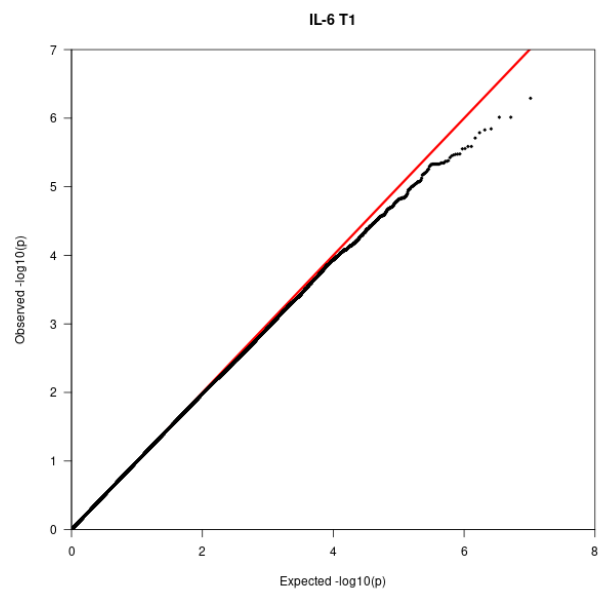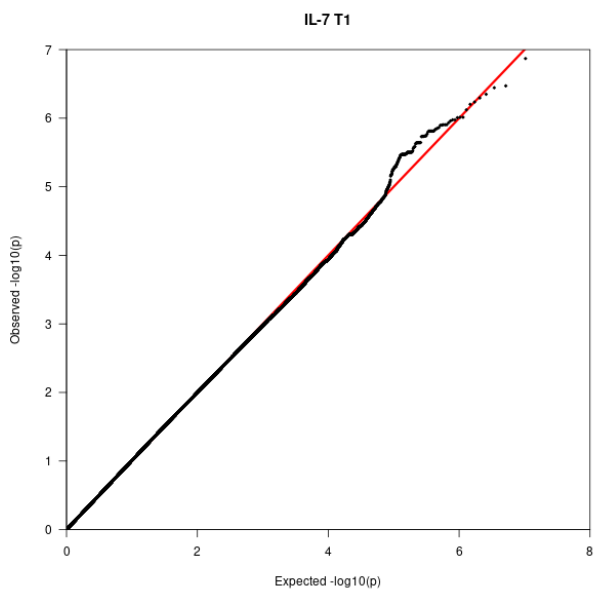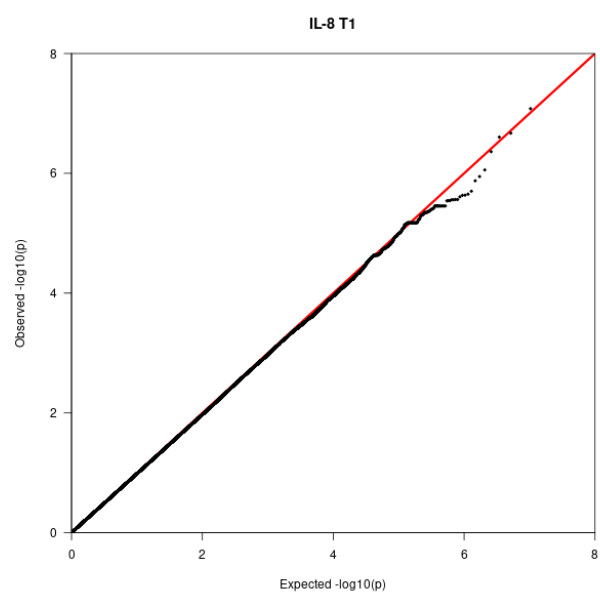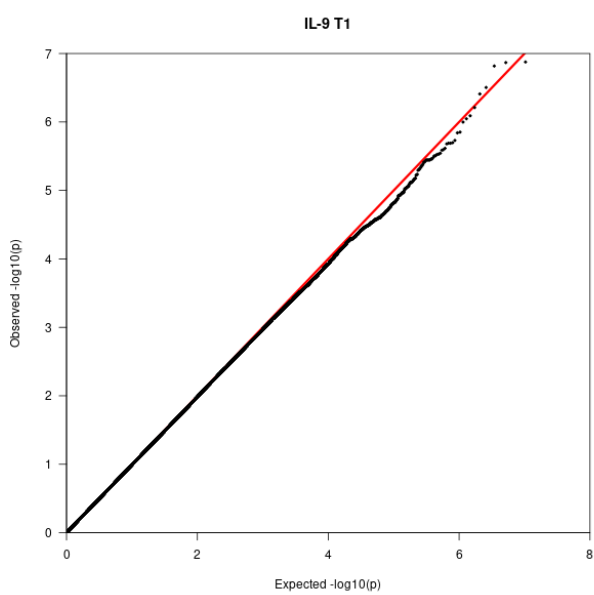

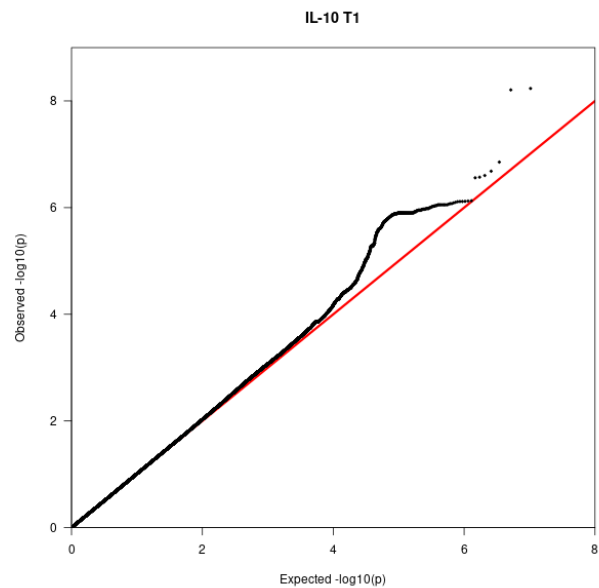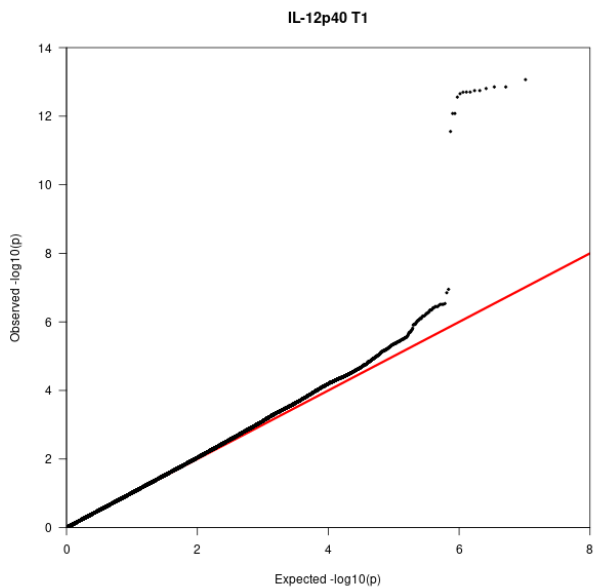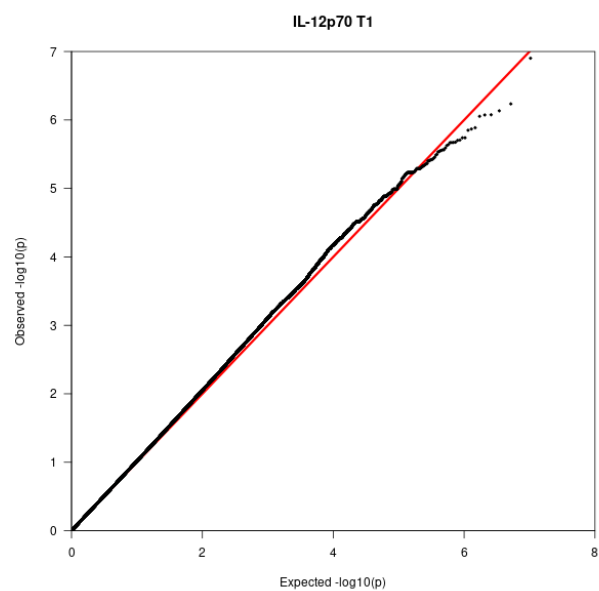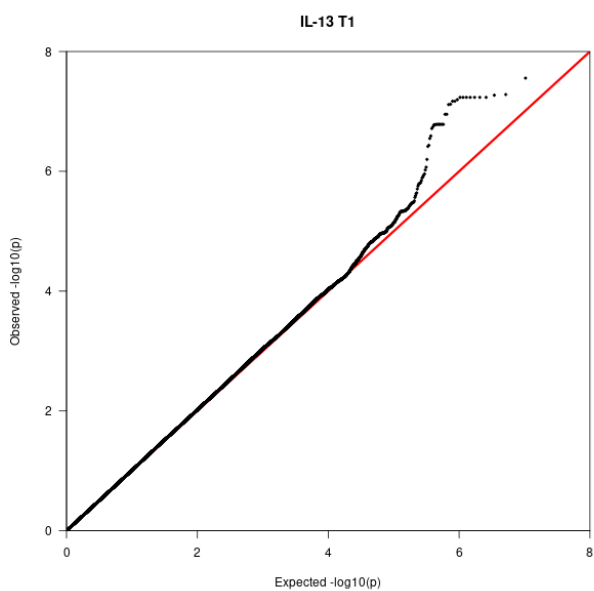

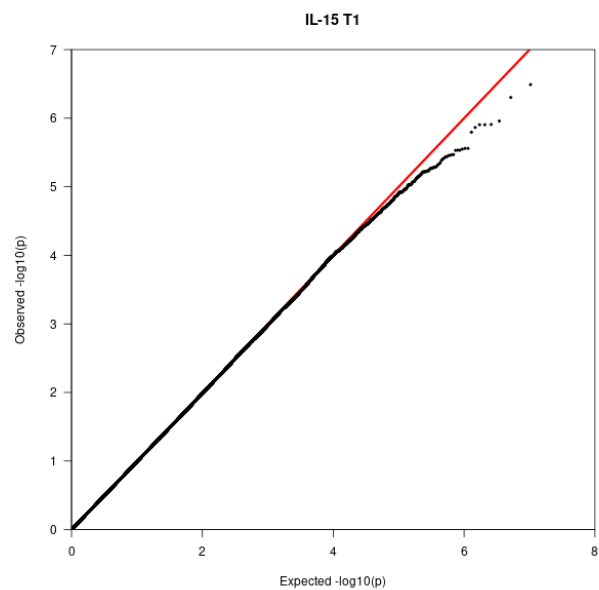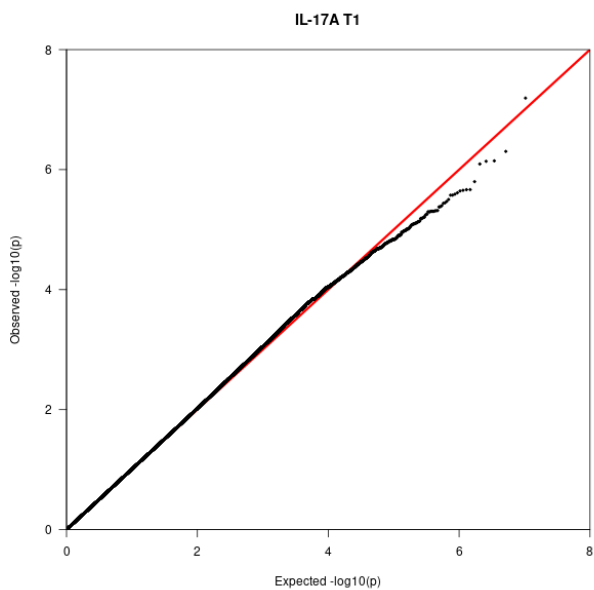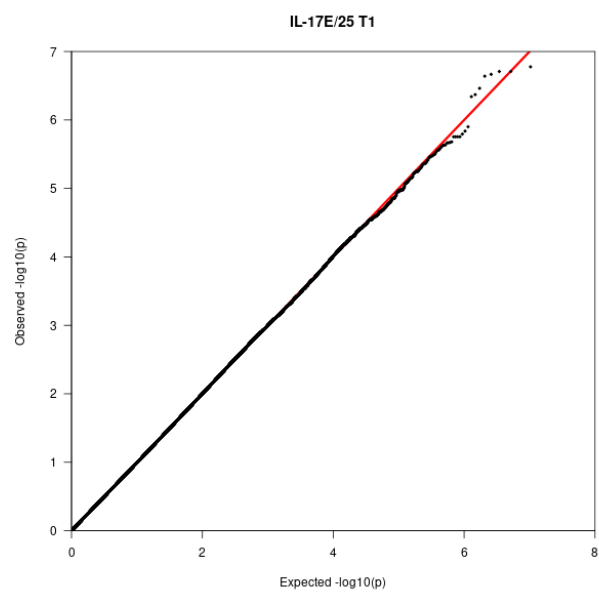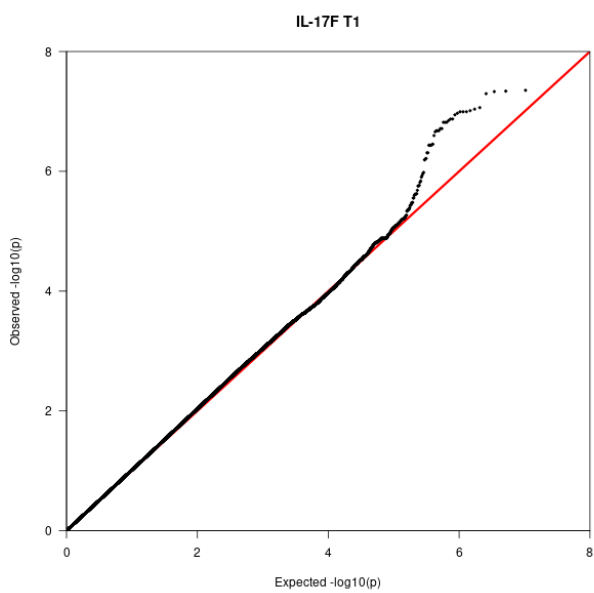

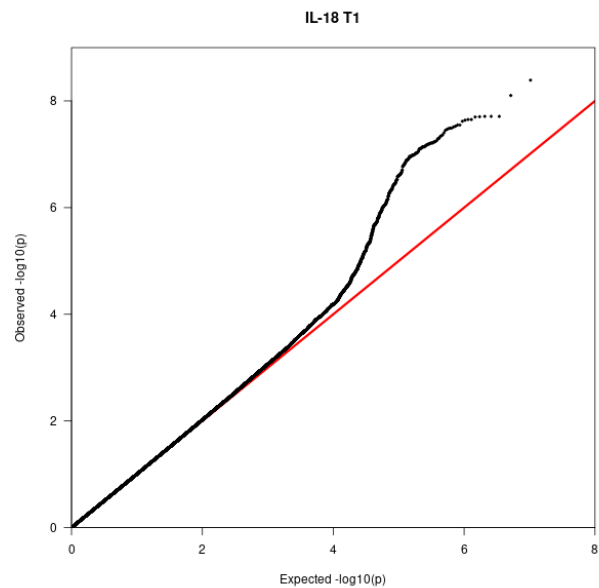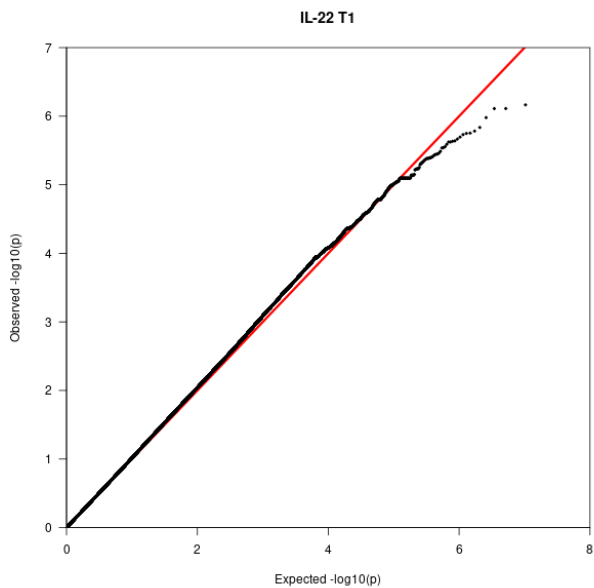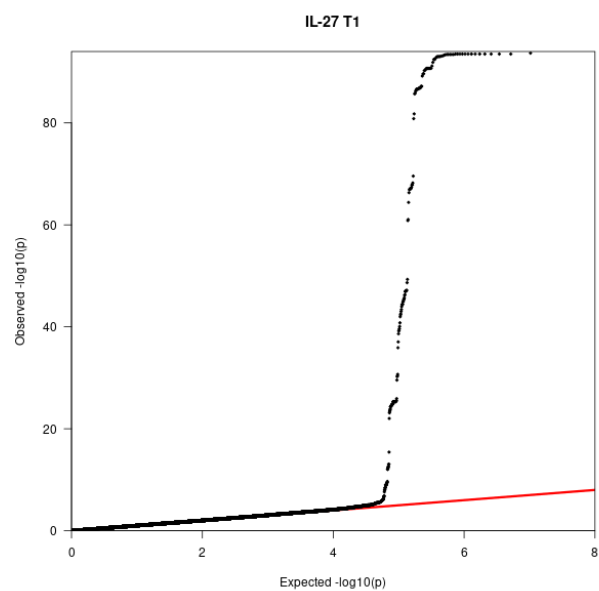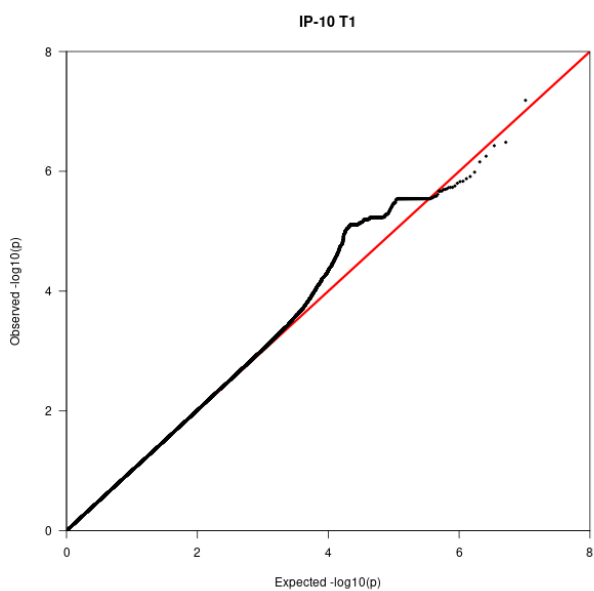

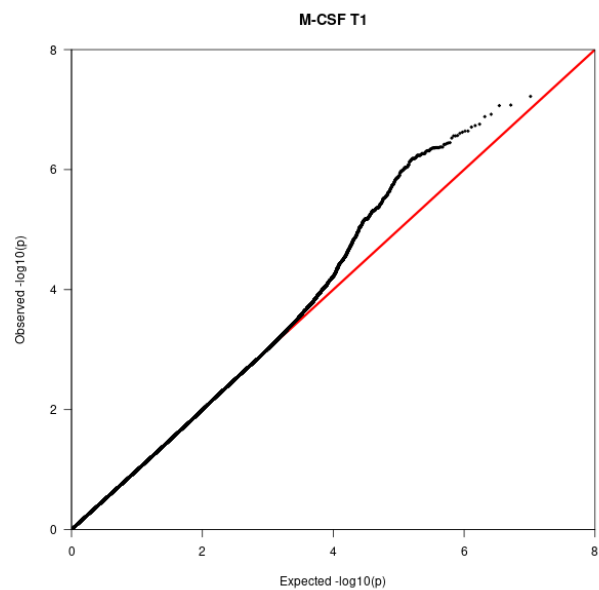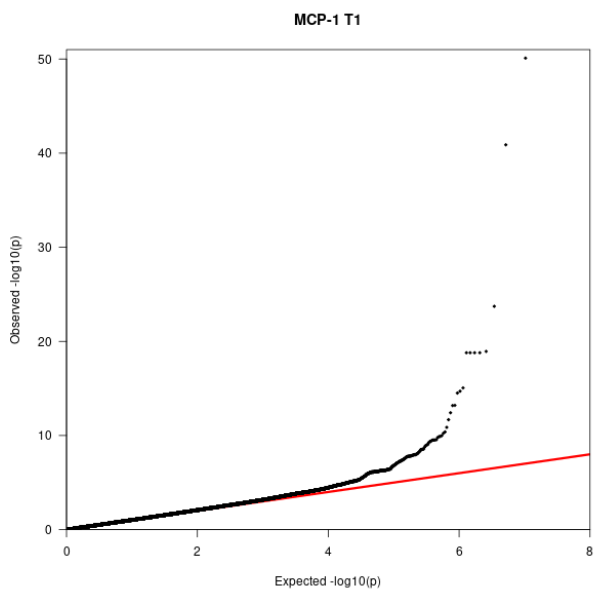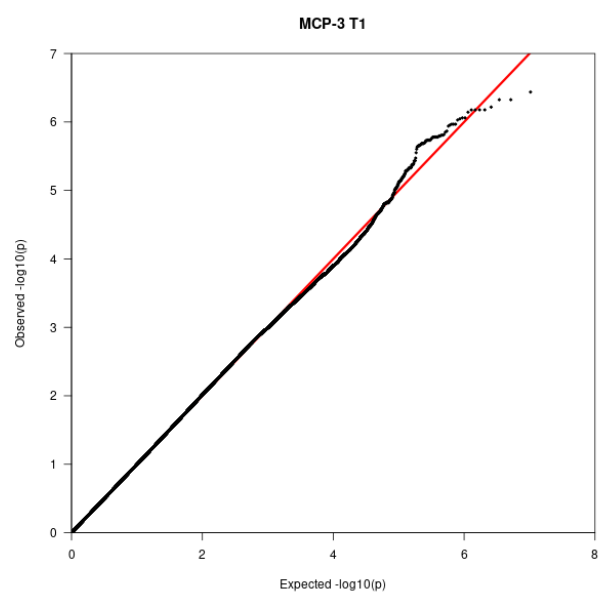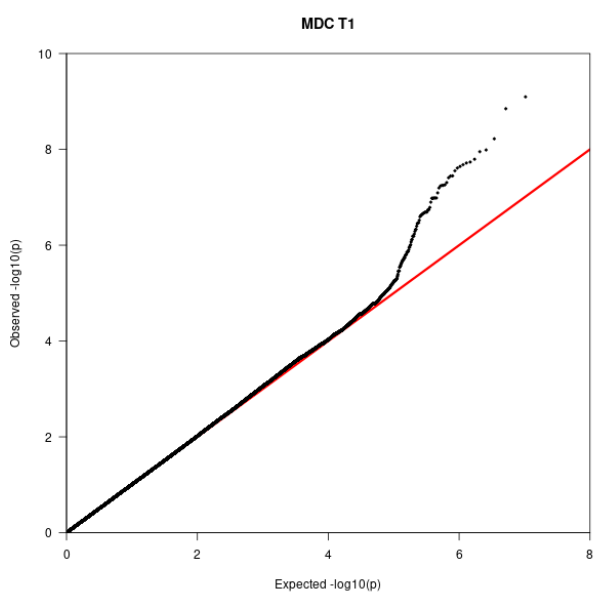

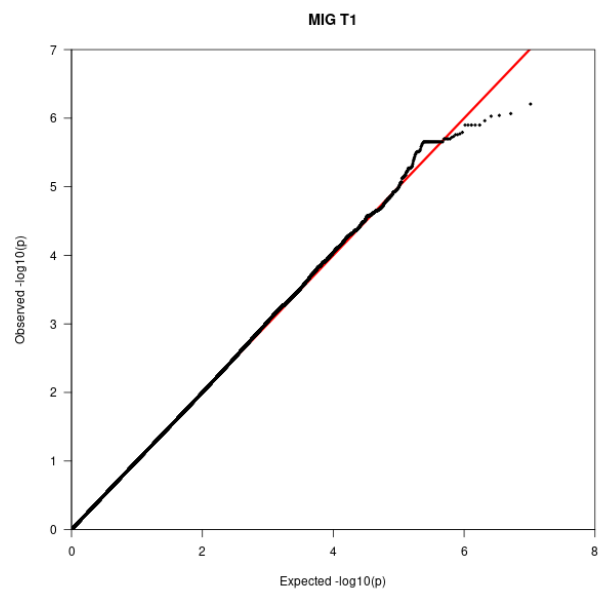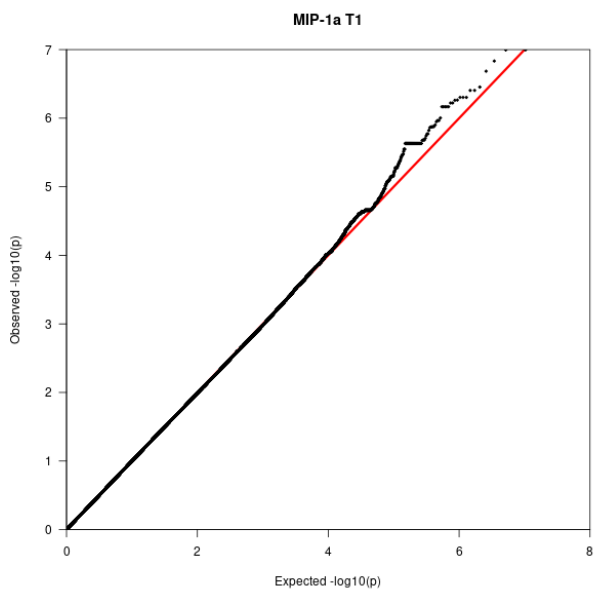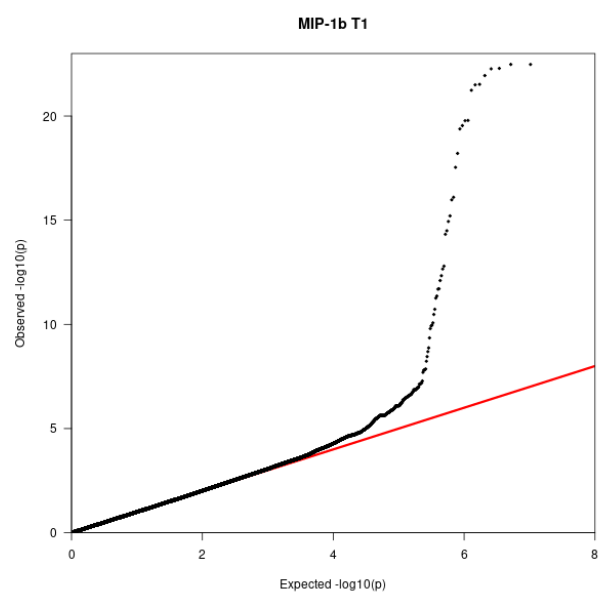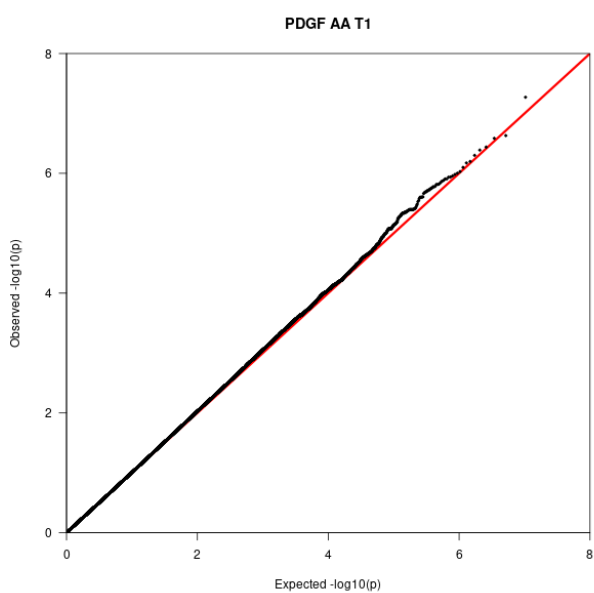

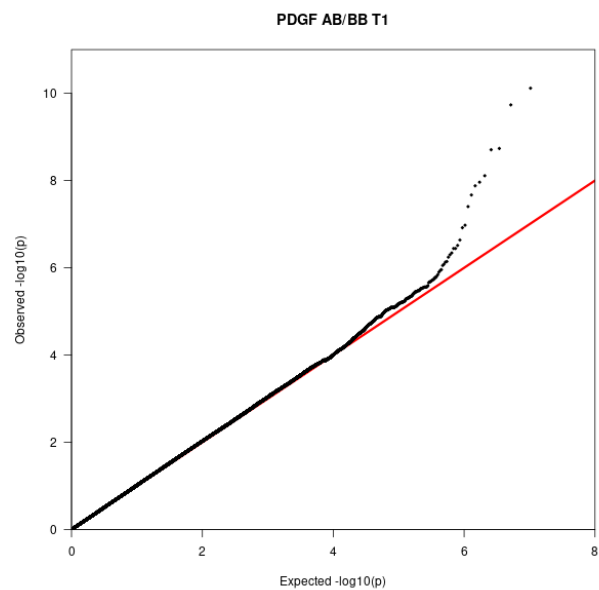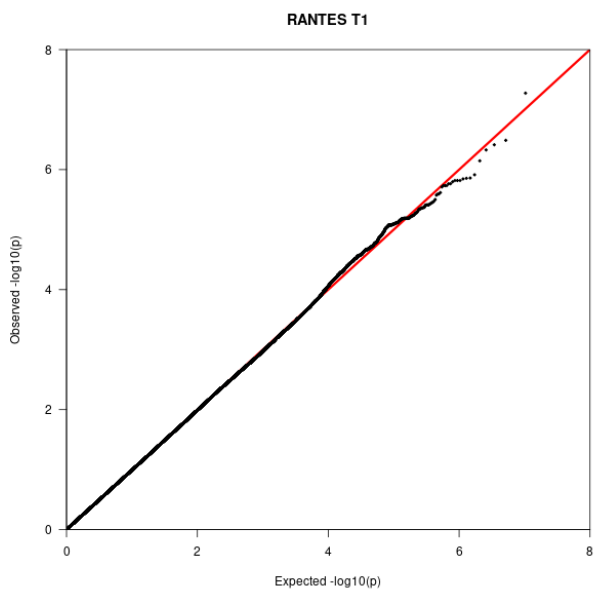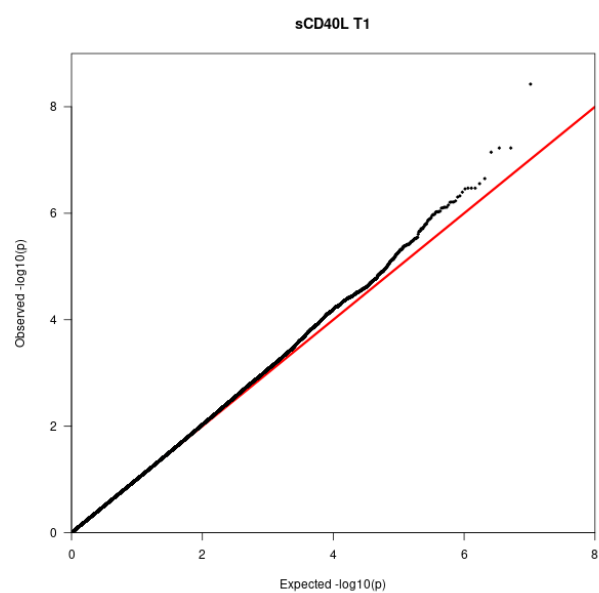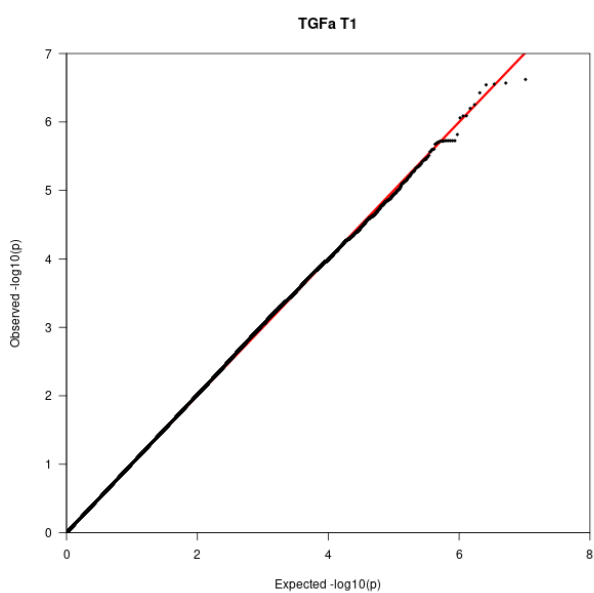

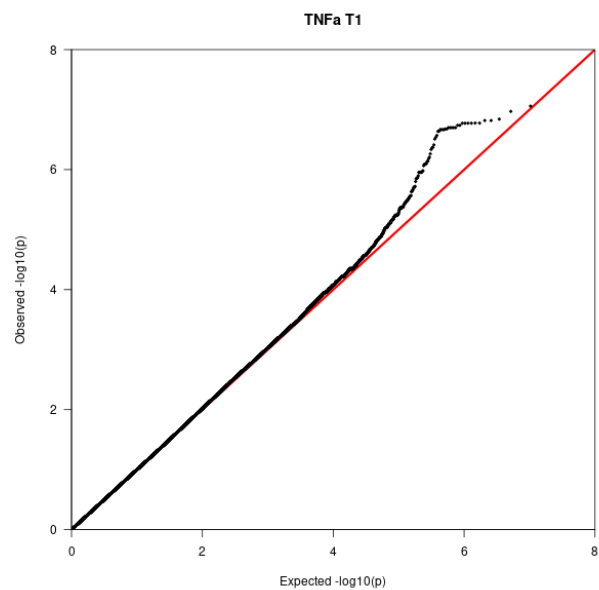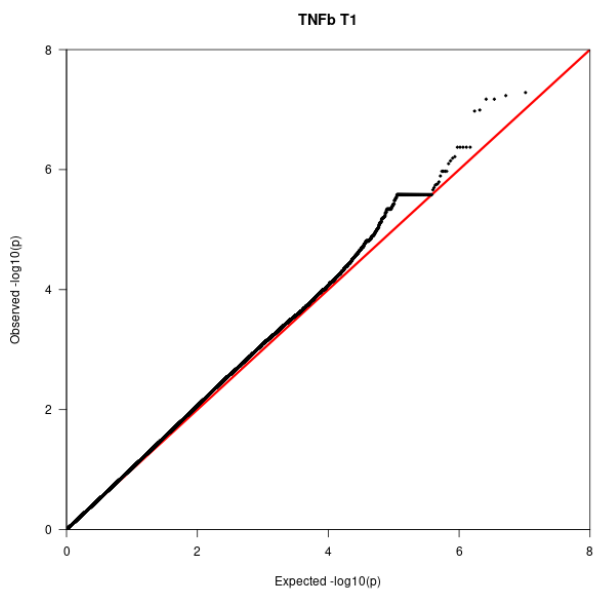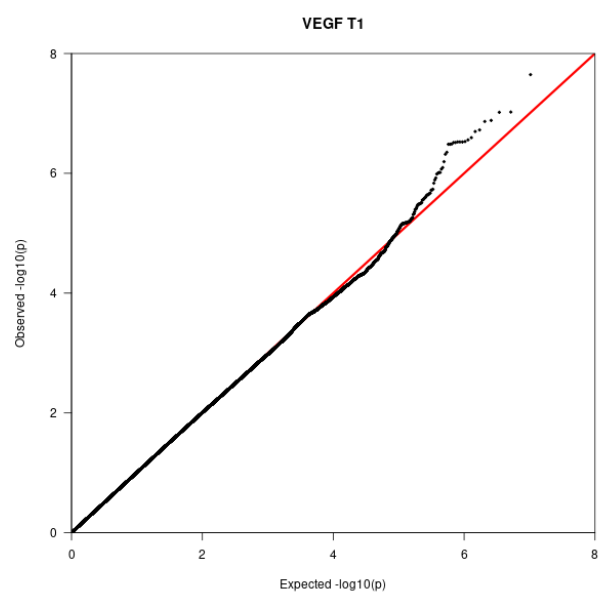

**b**

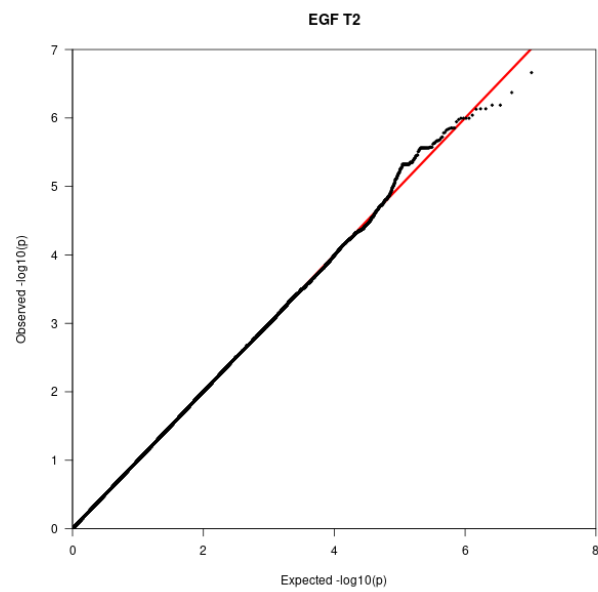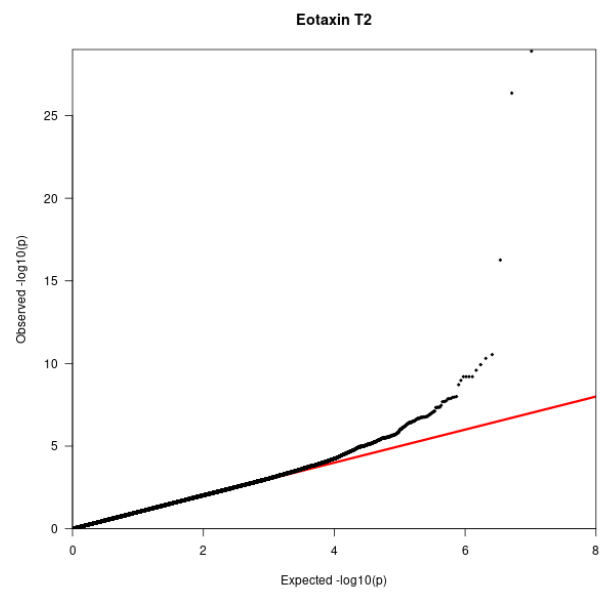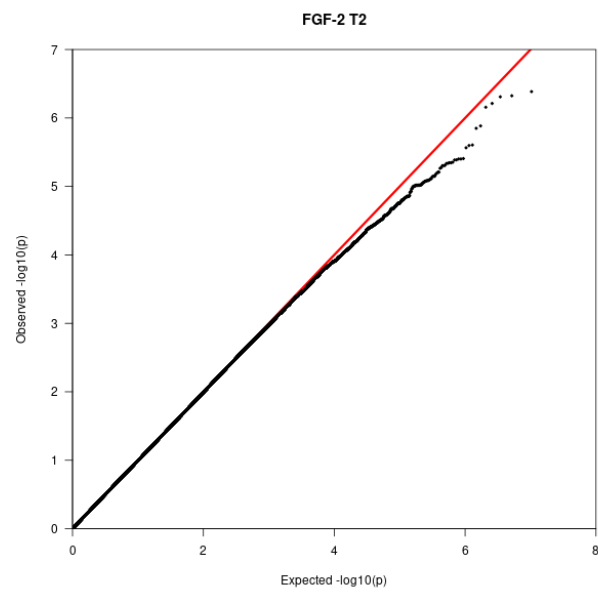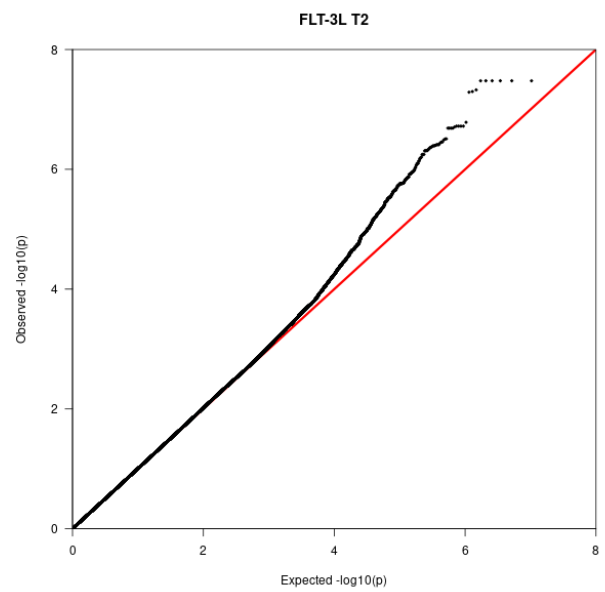

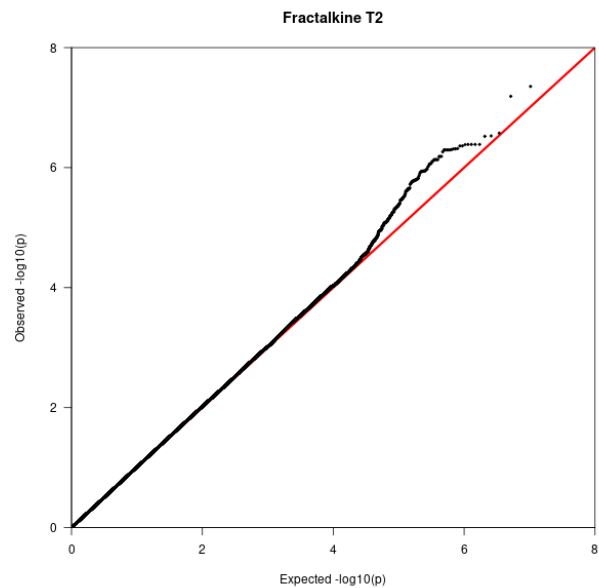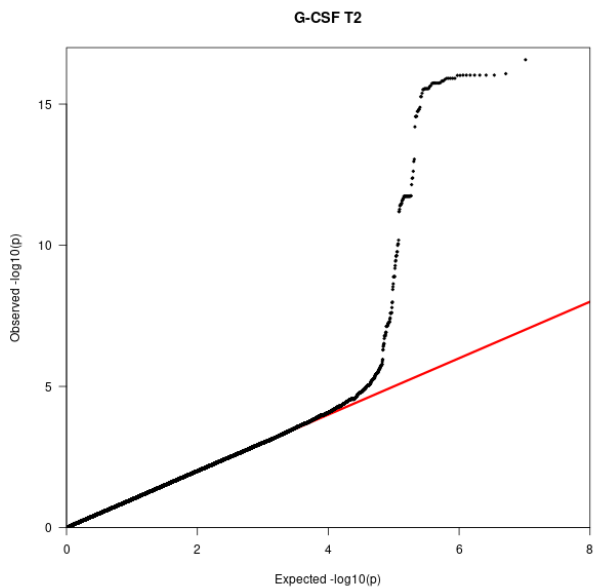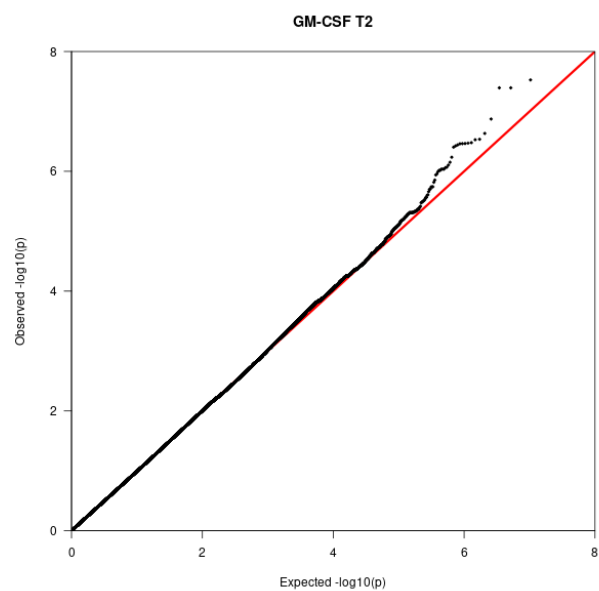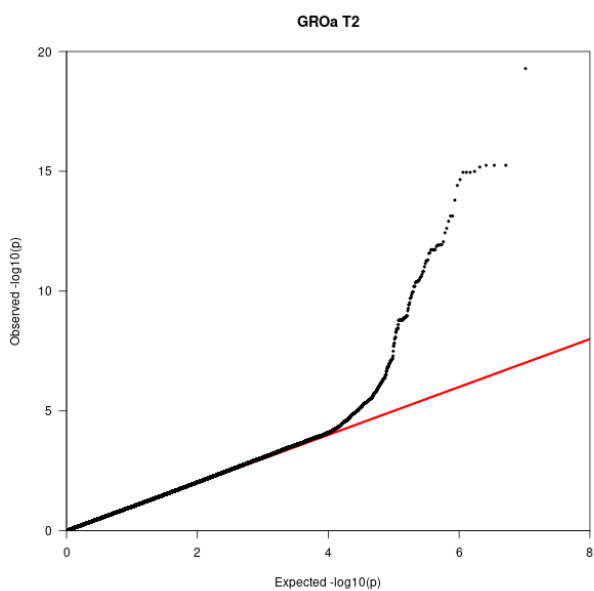

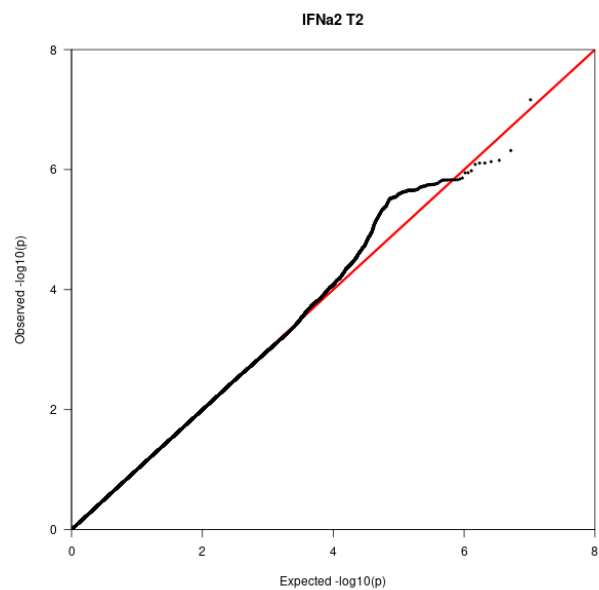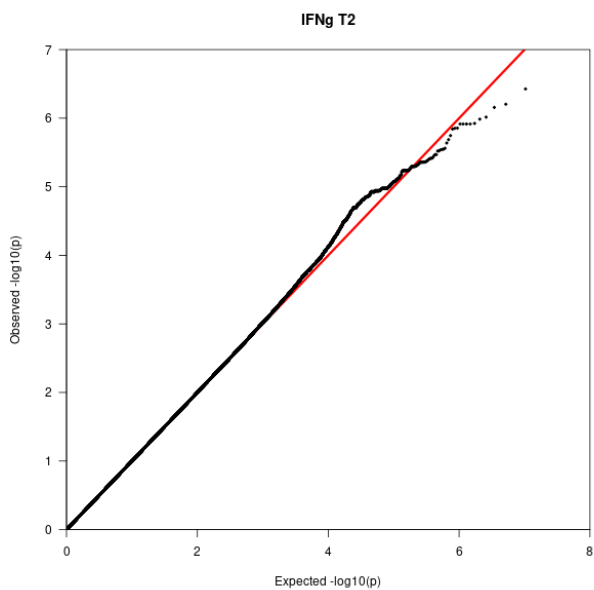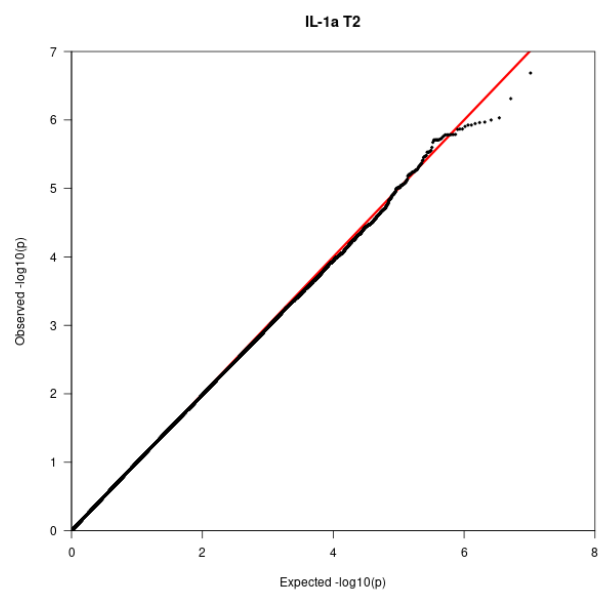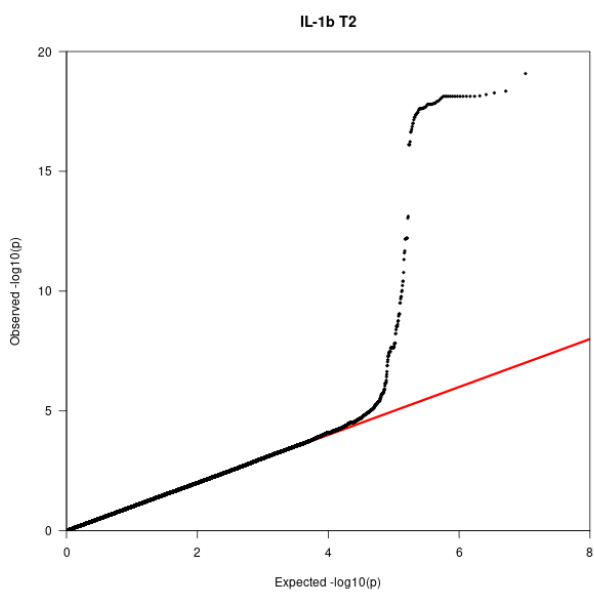

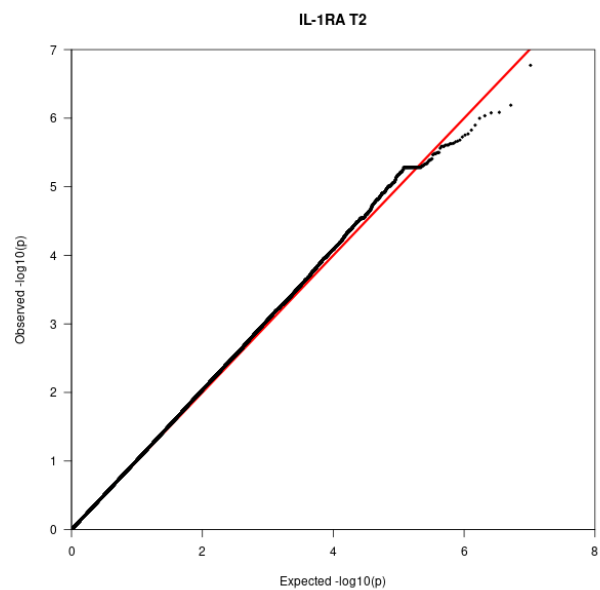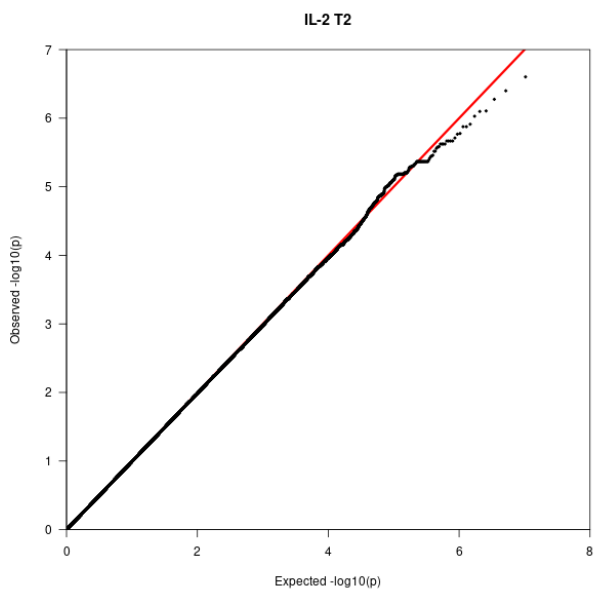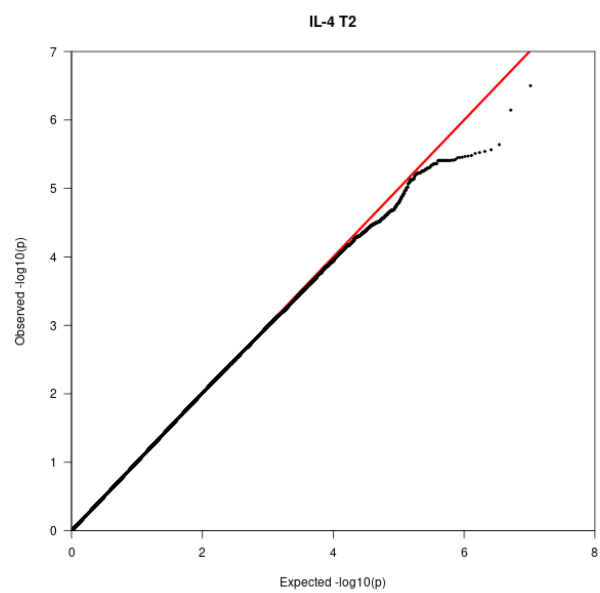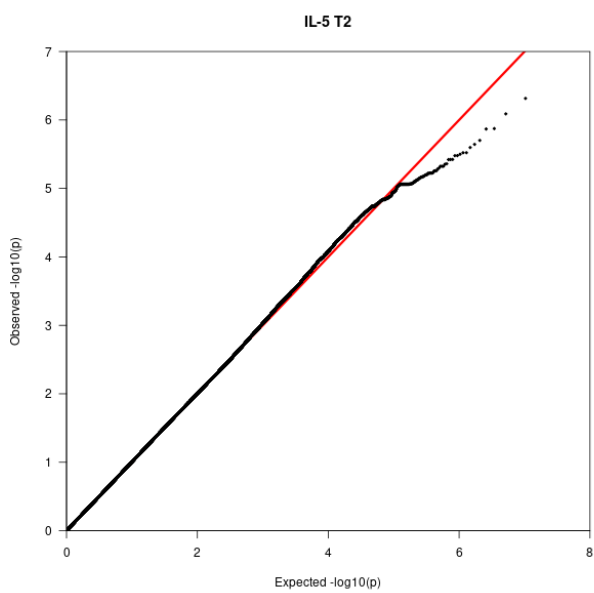

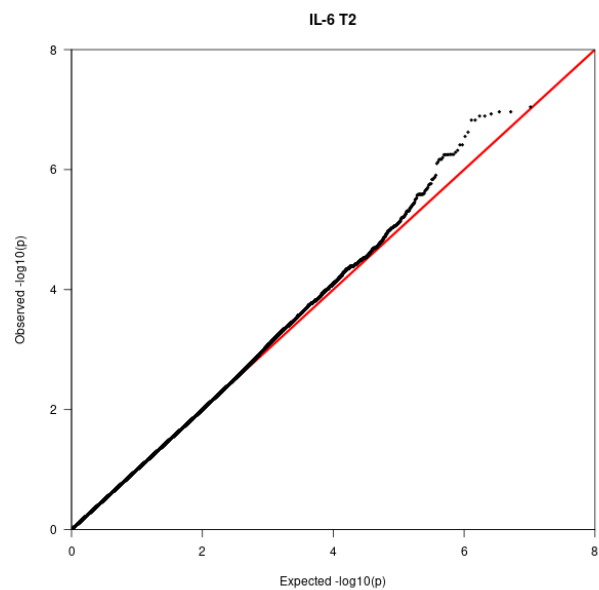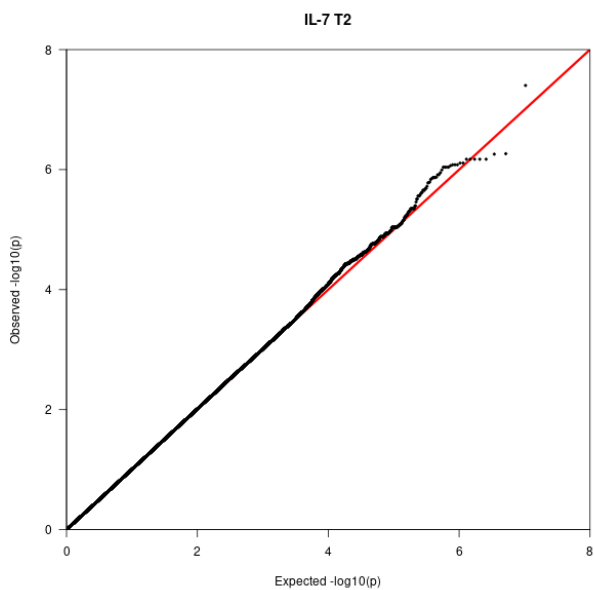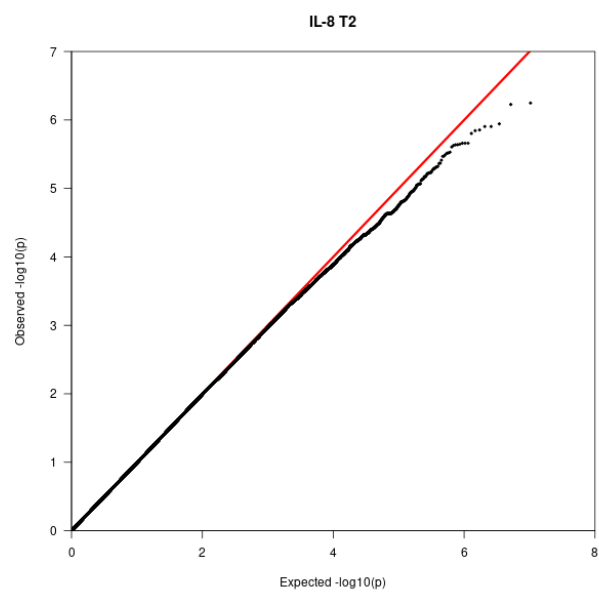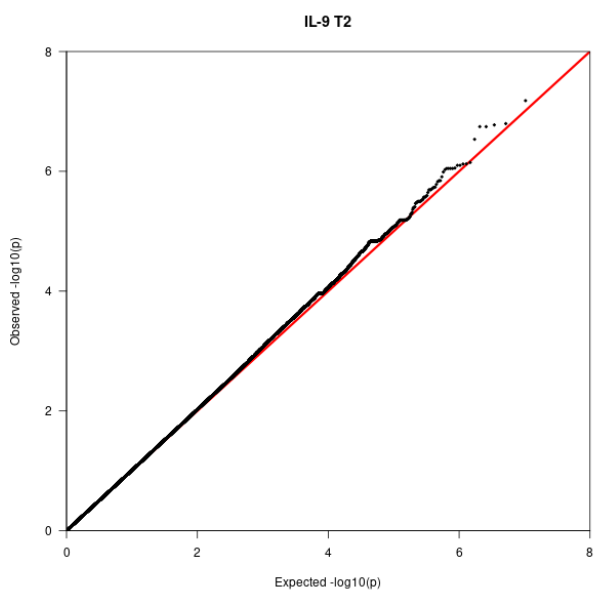

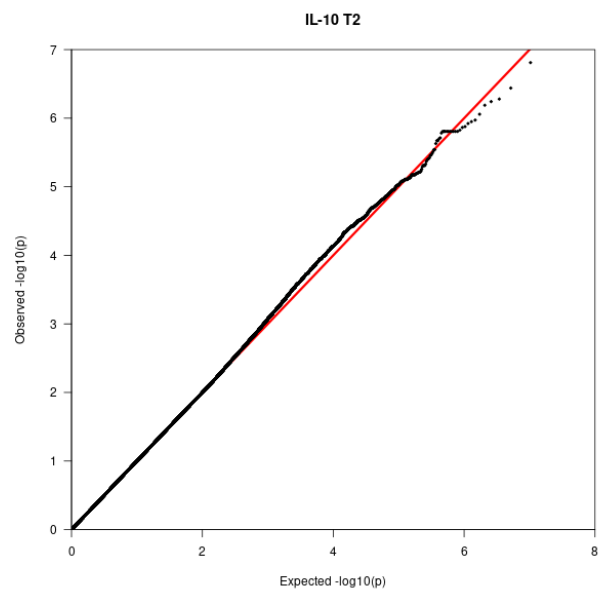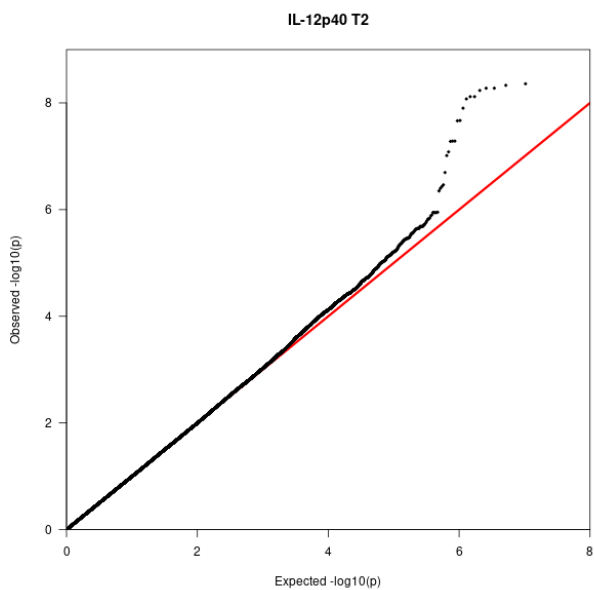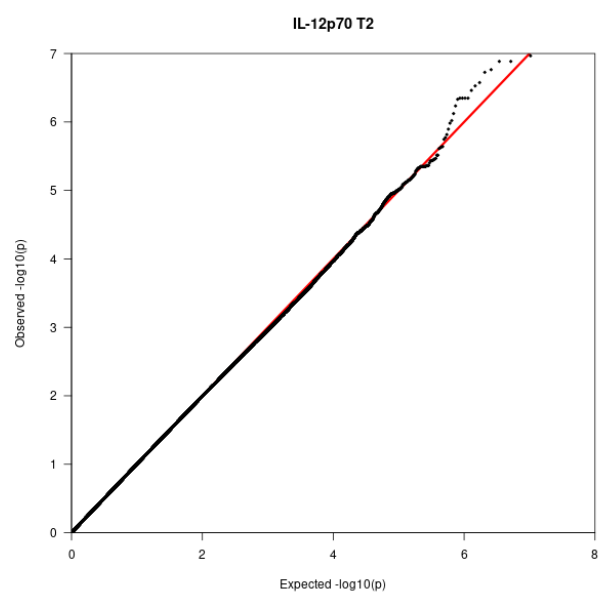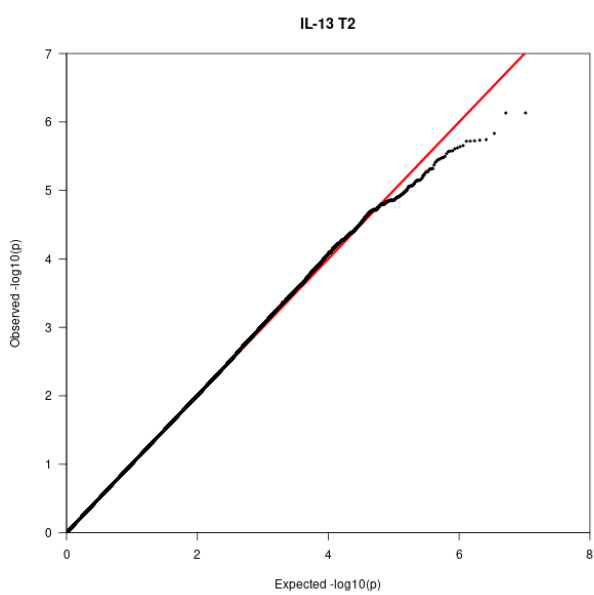

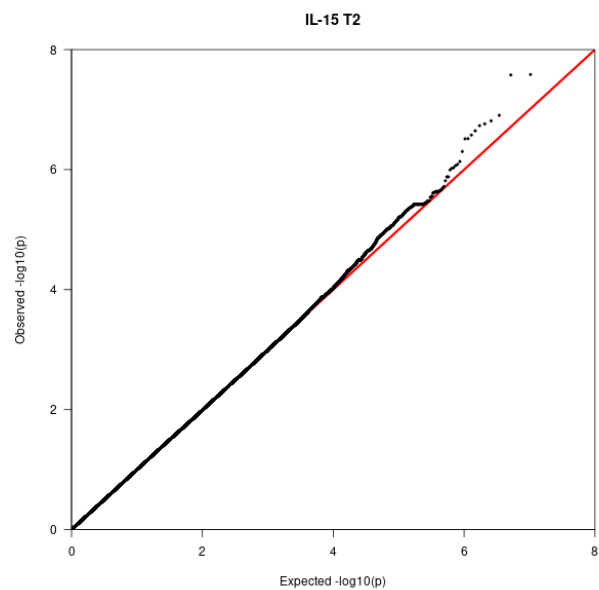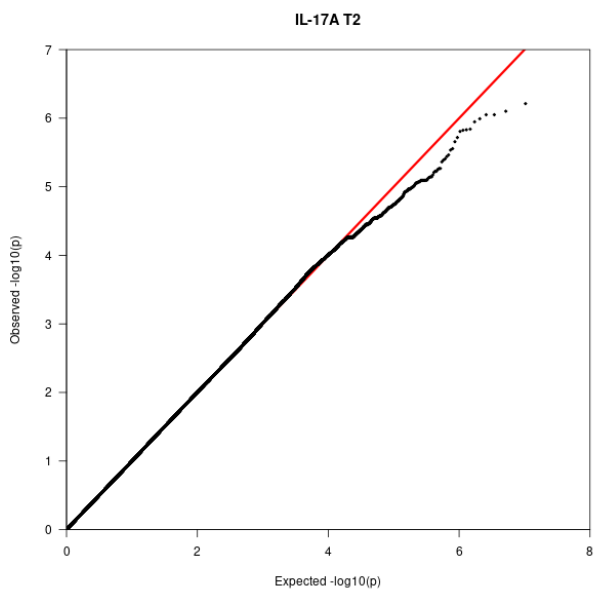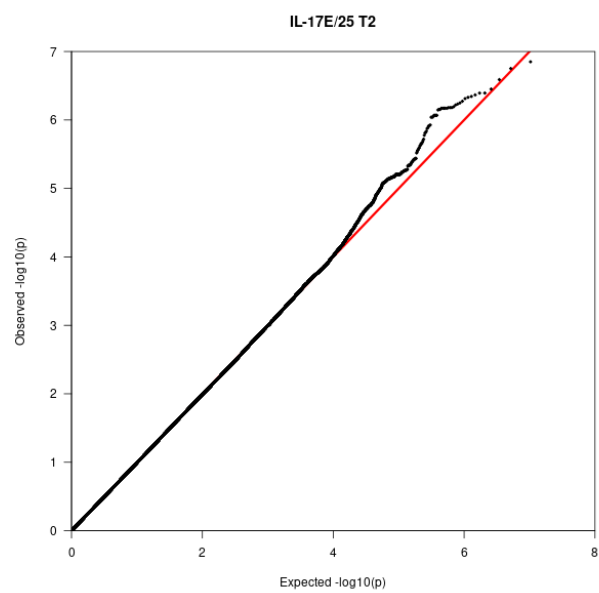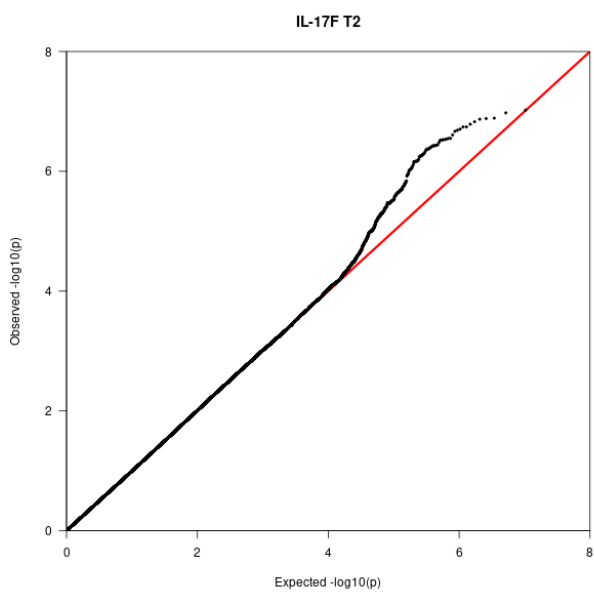

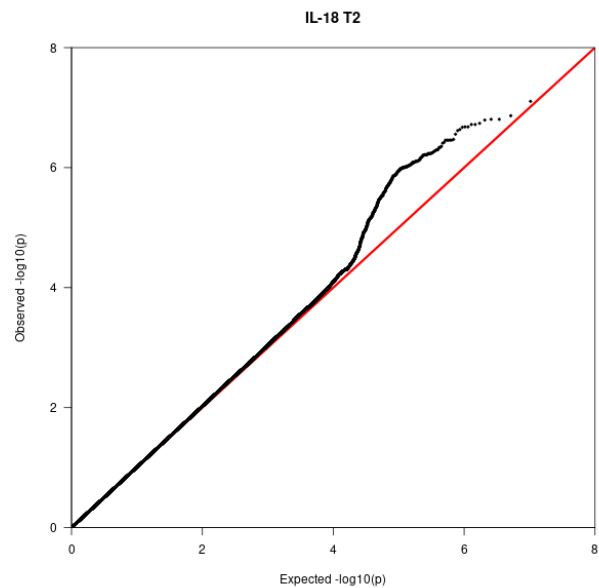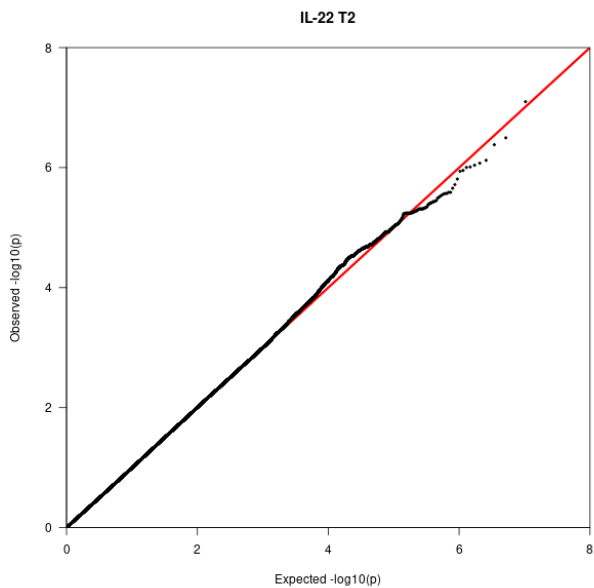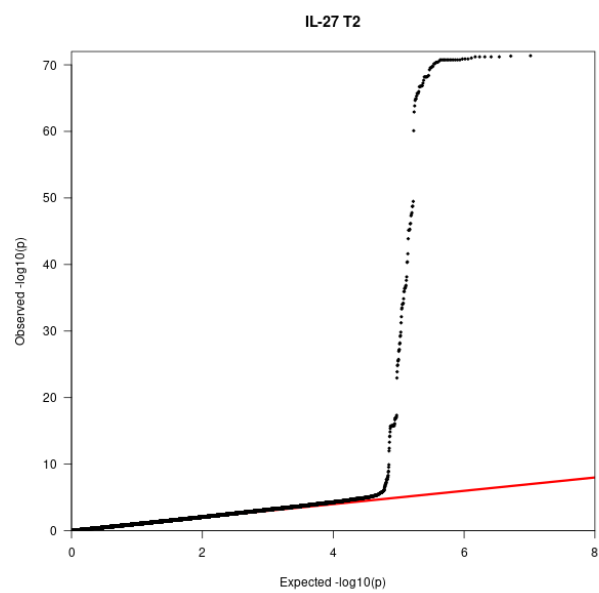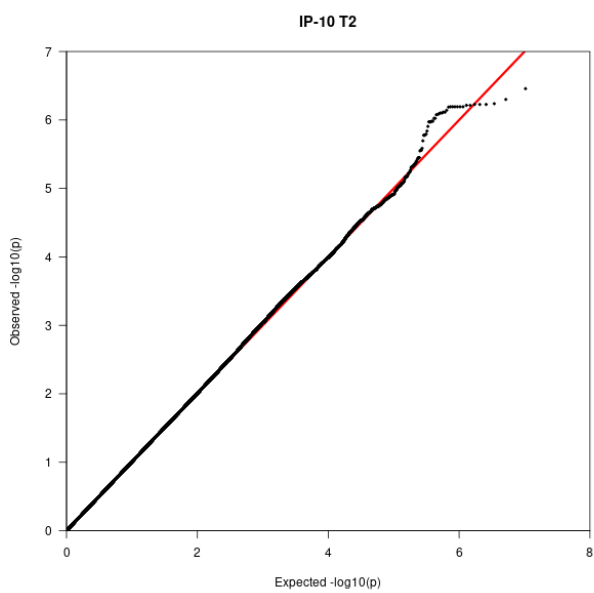

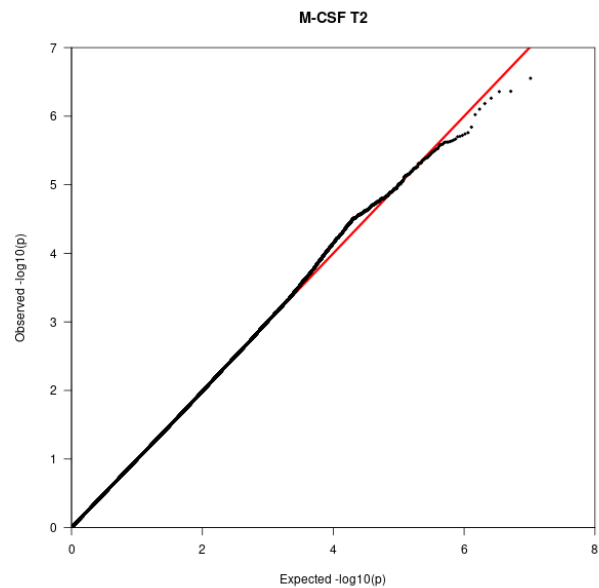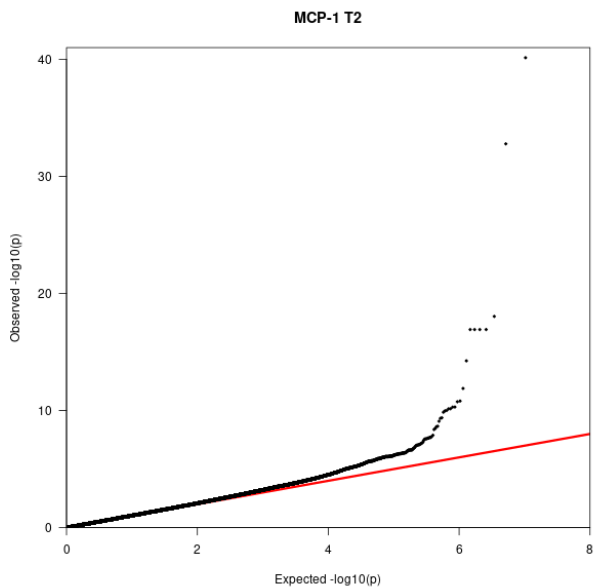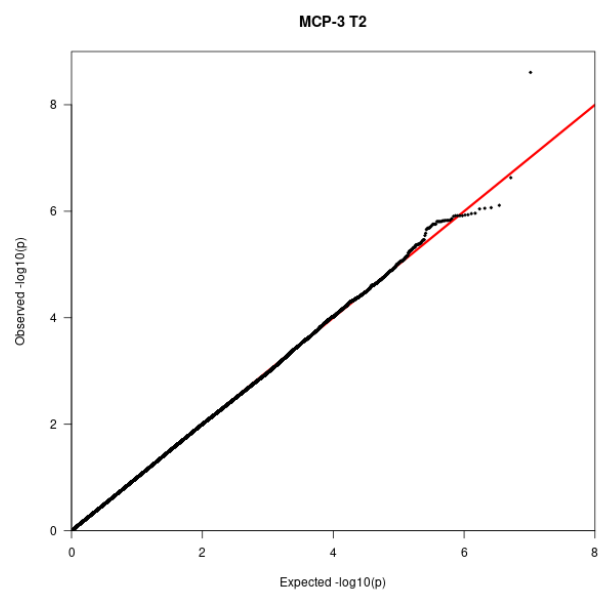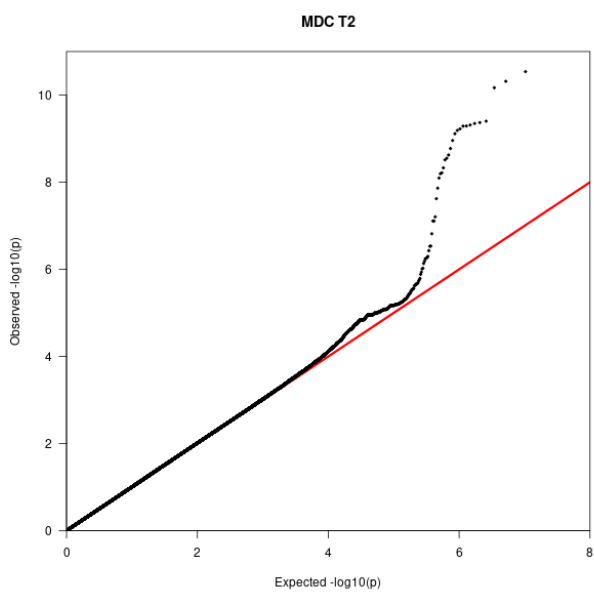

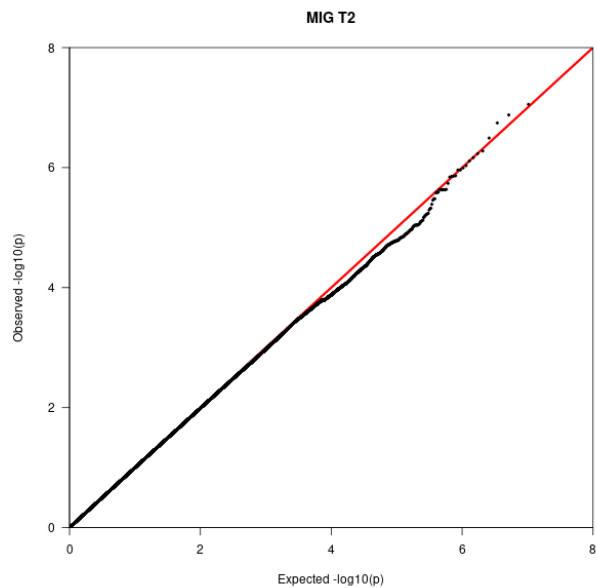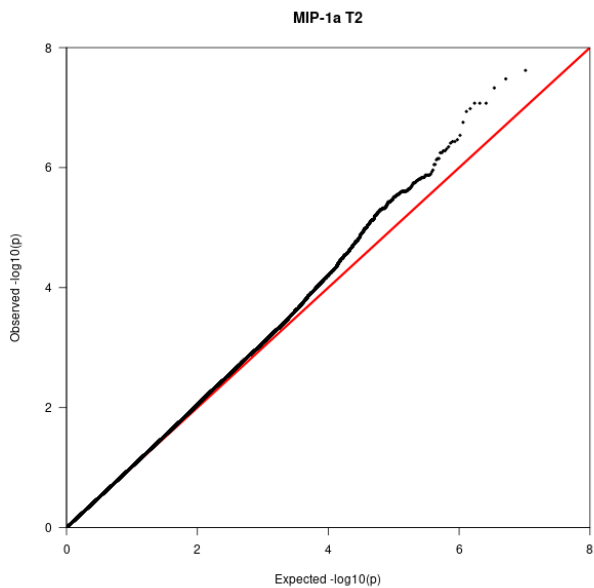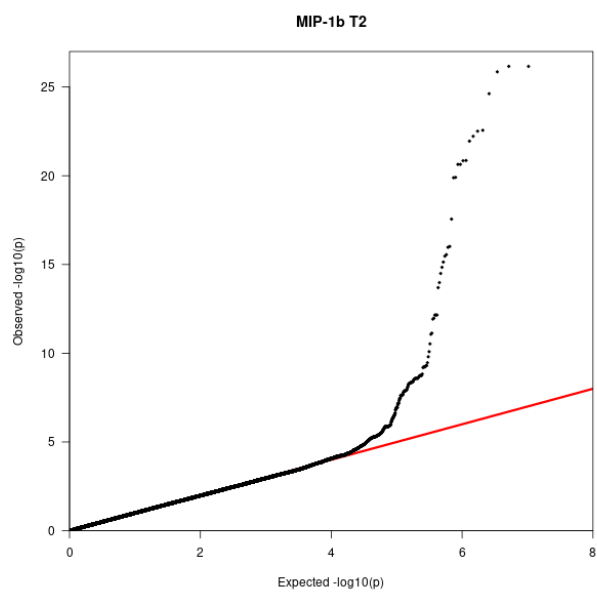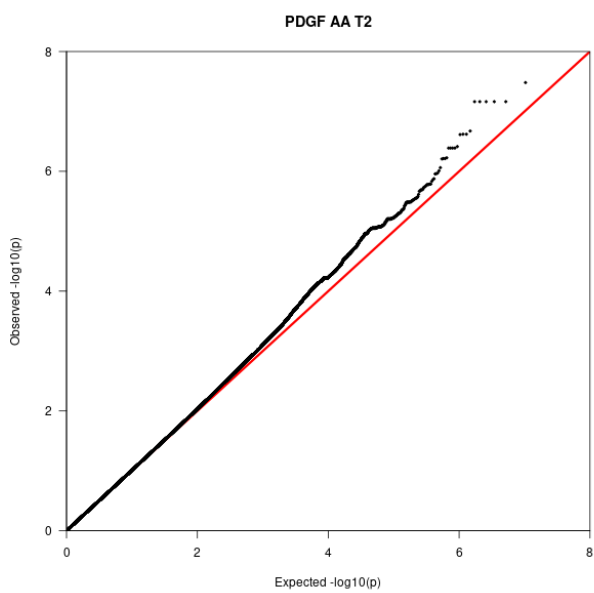

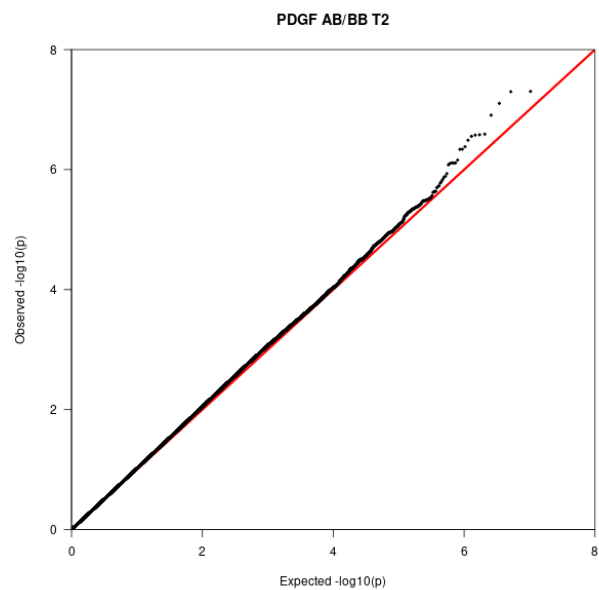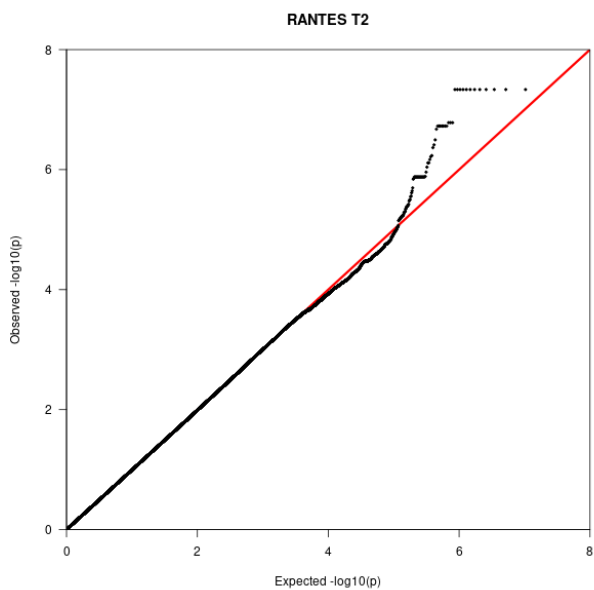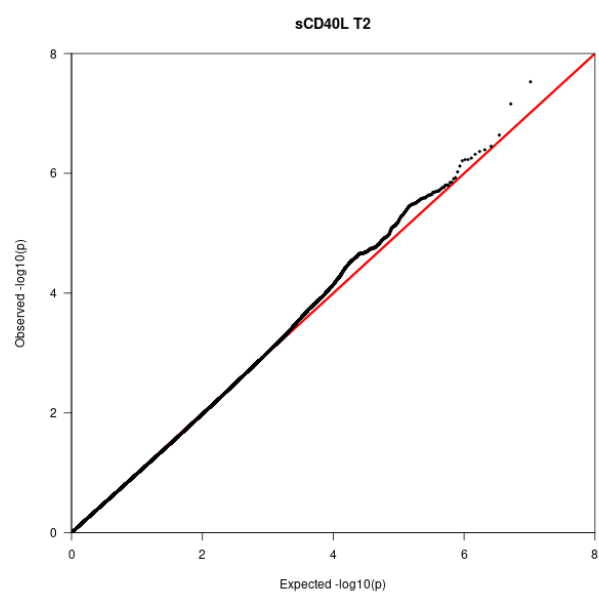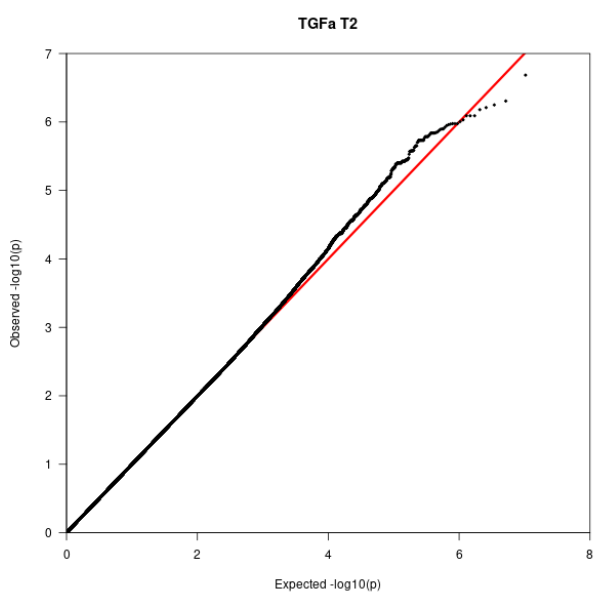

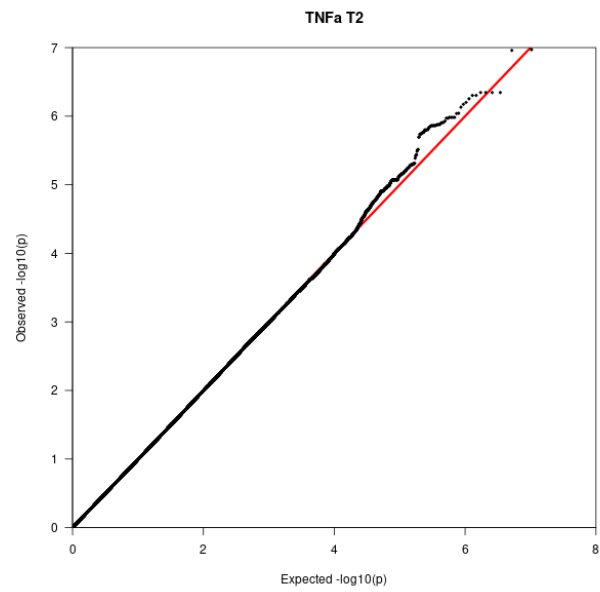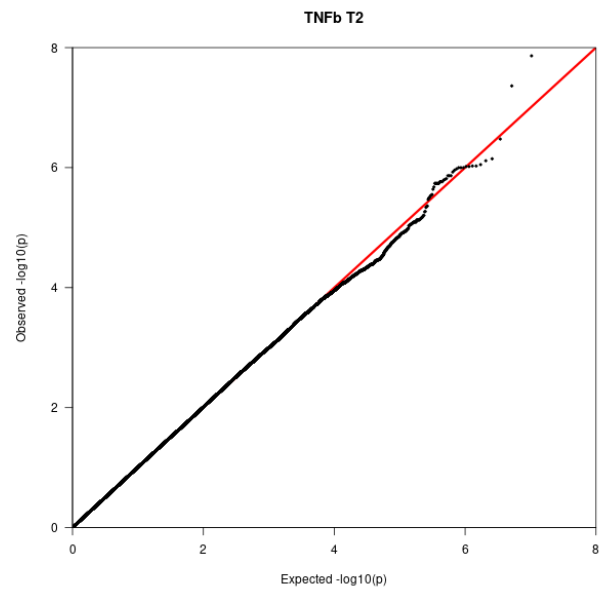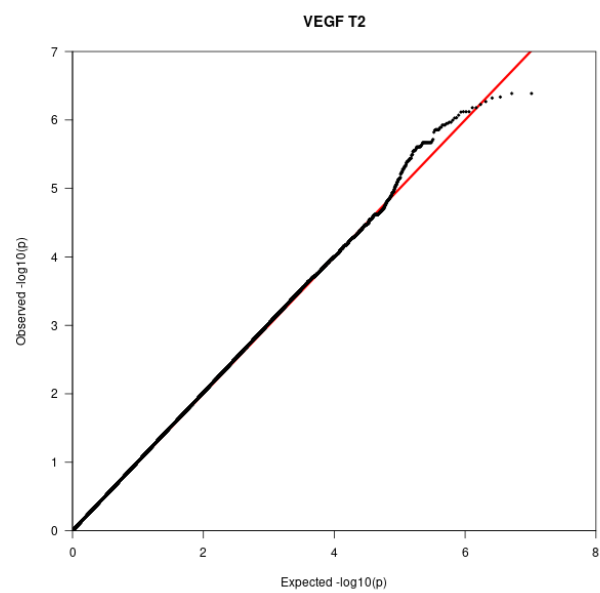

**C**

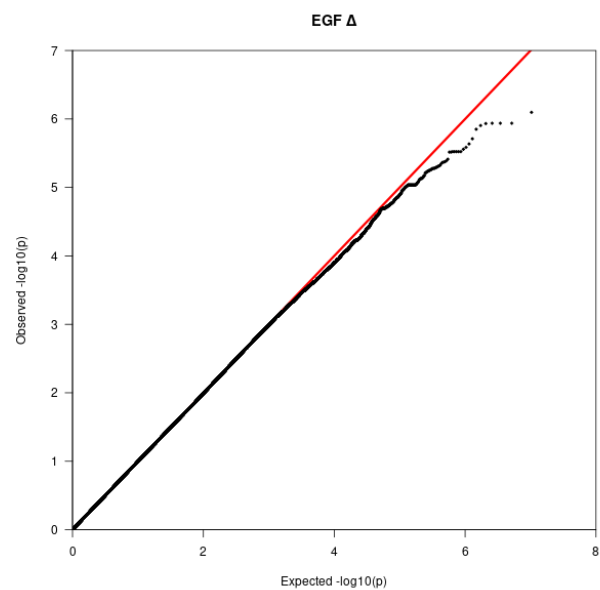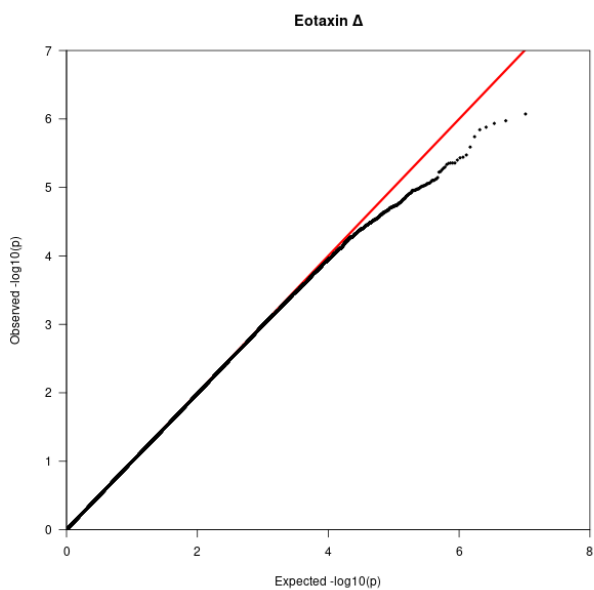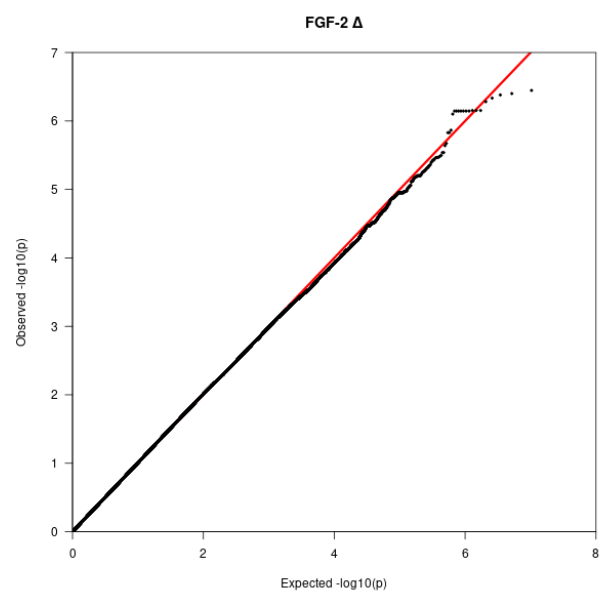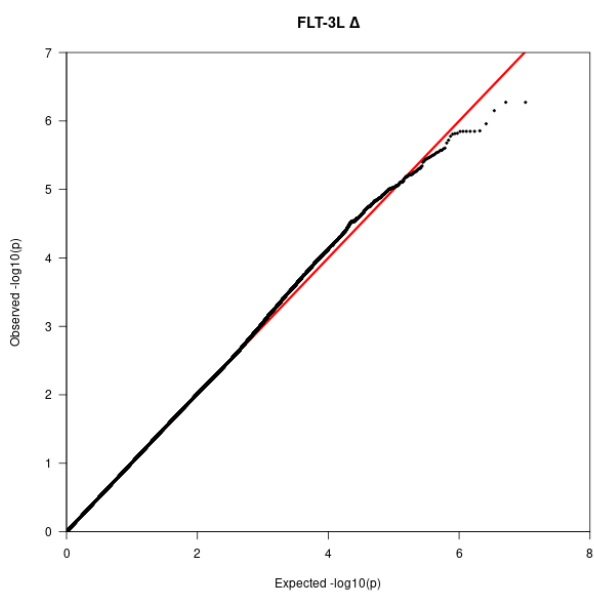

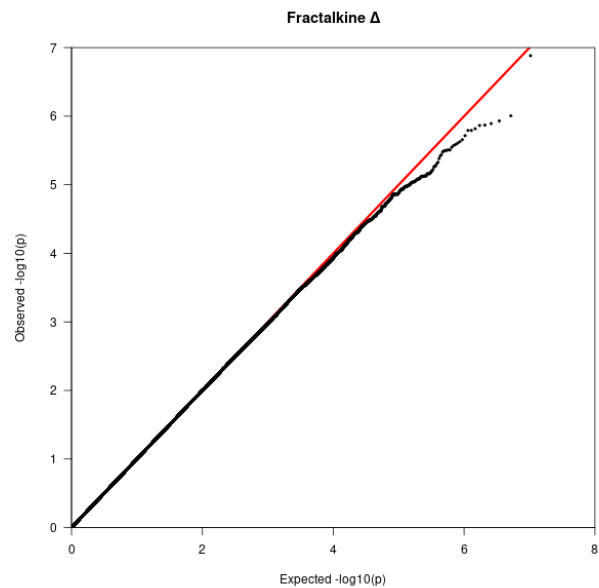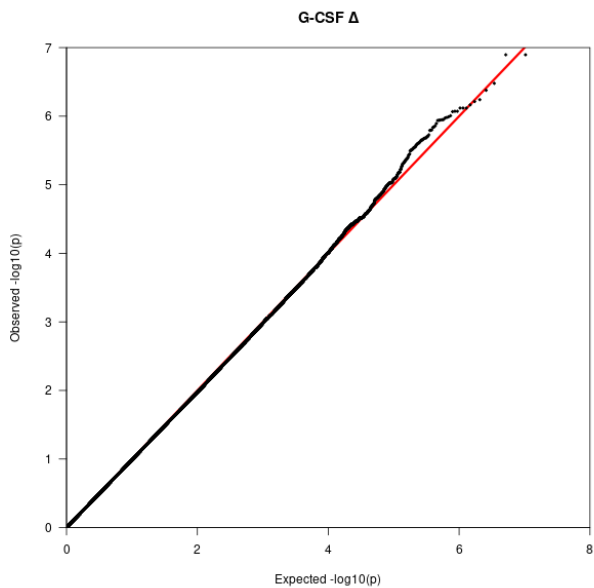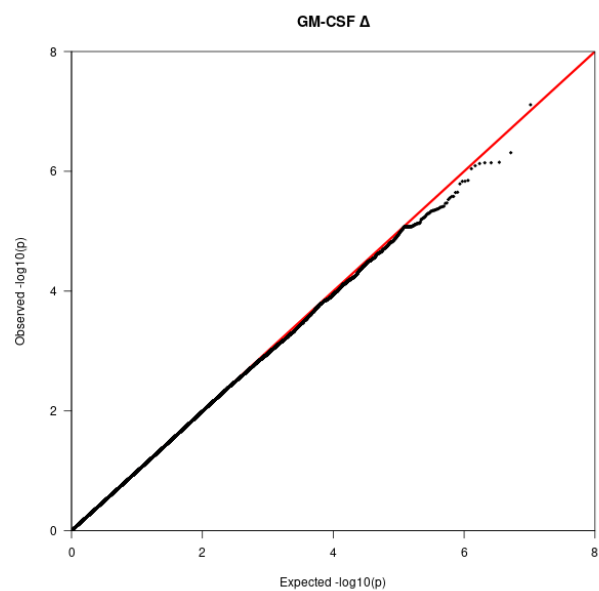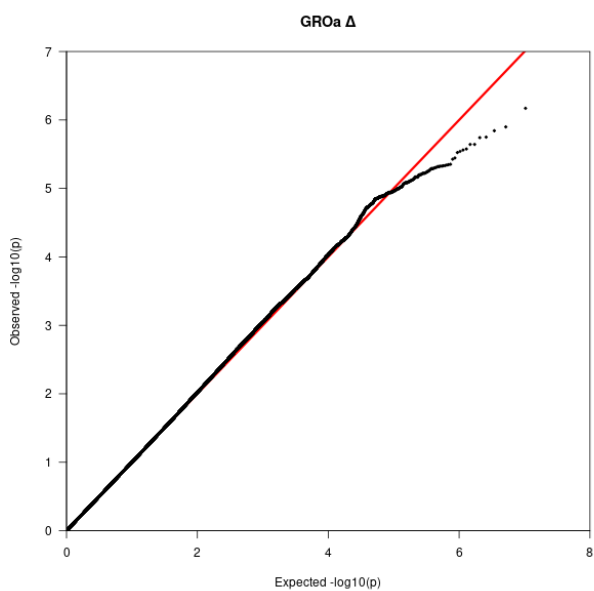

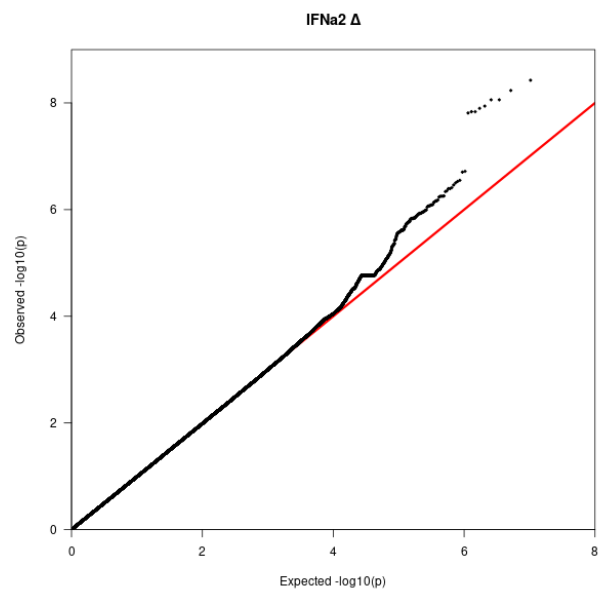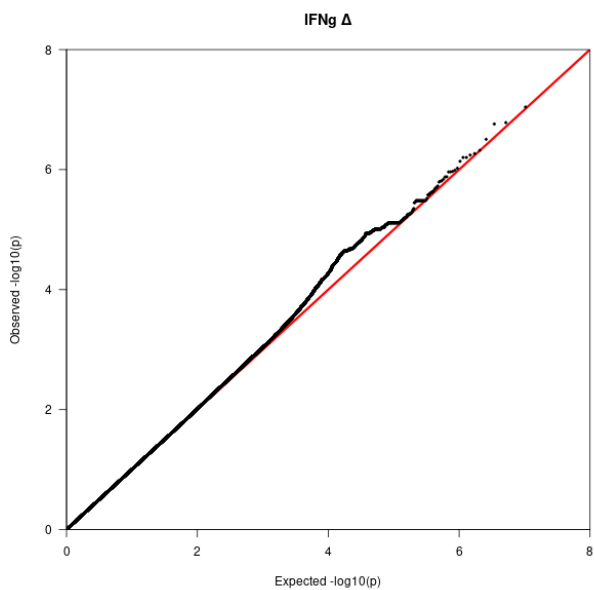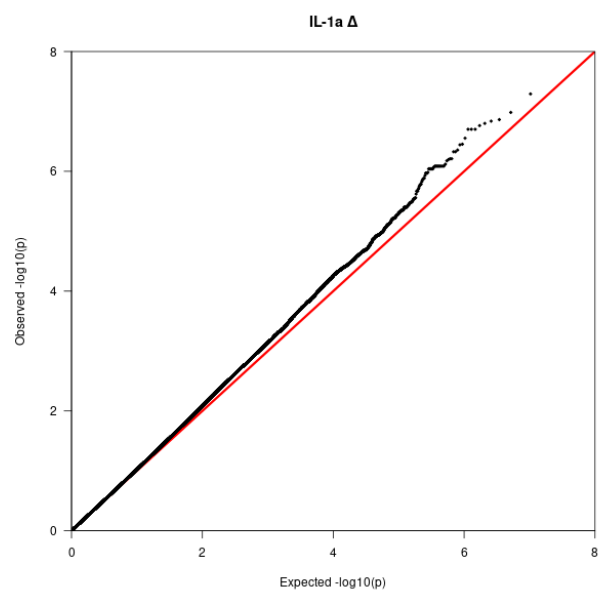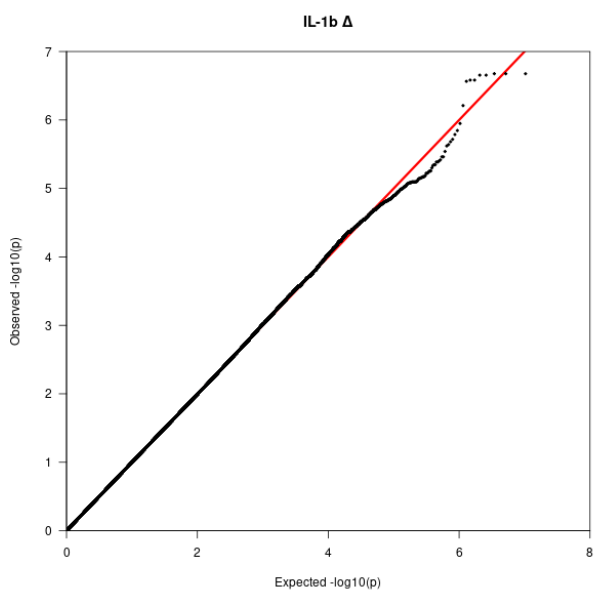

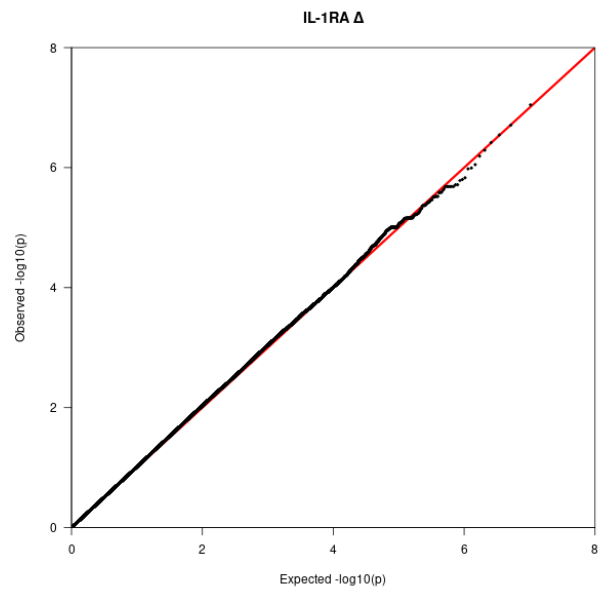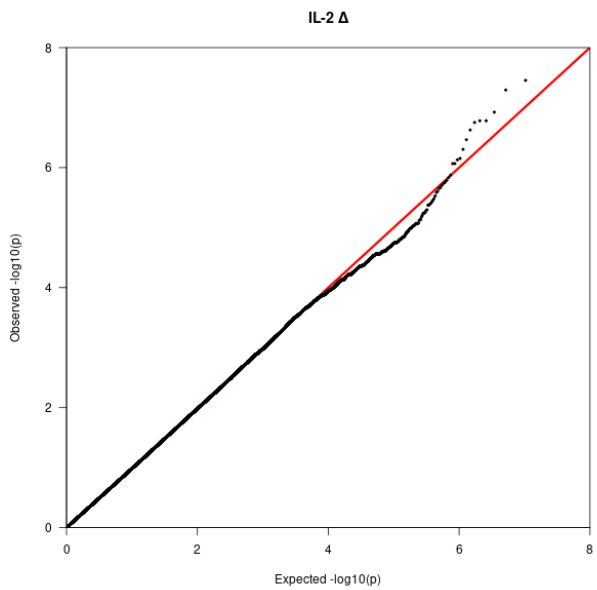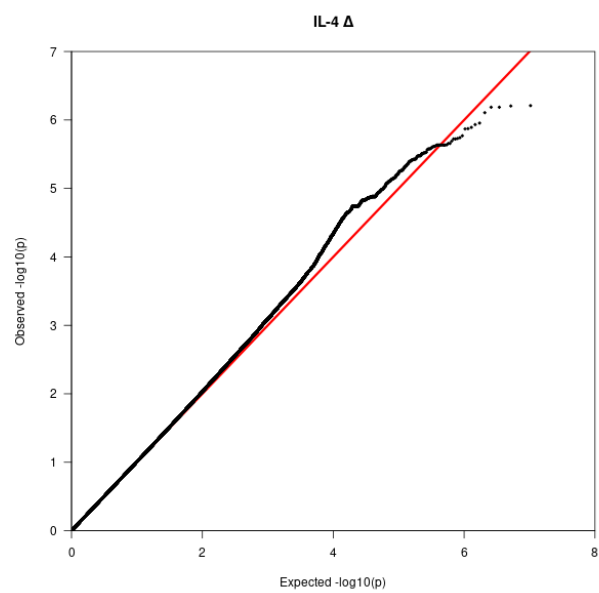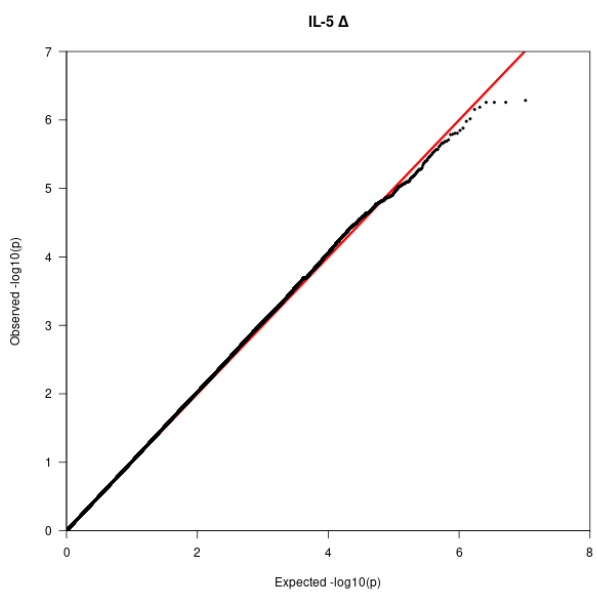

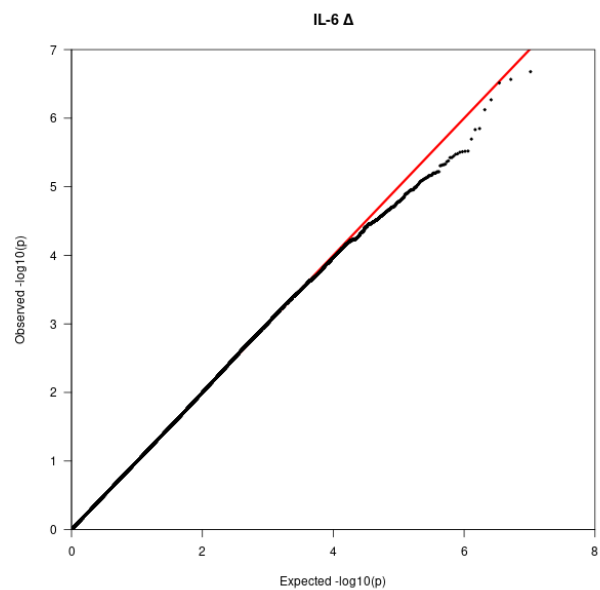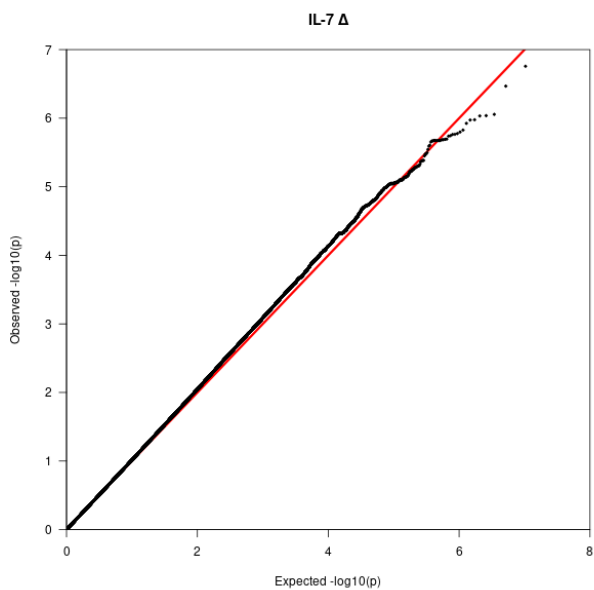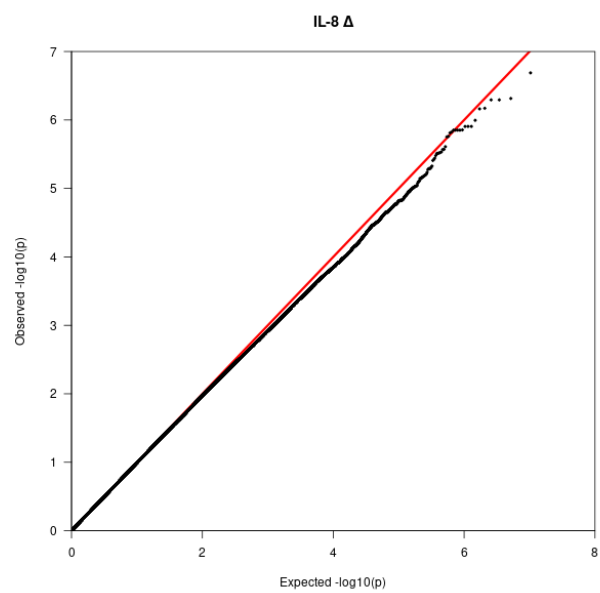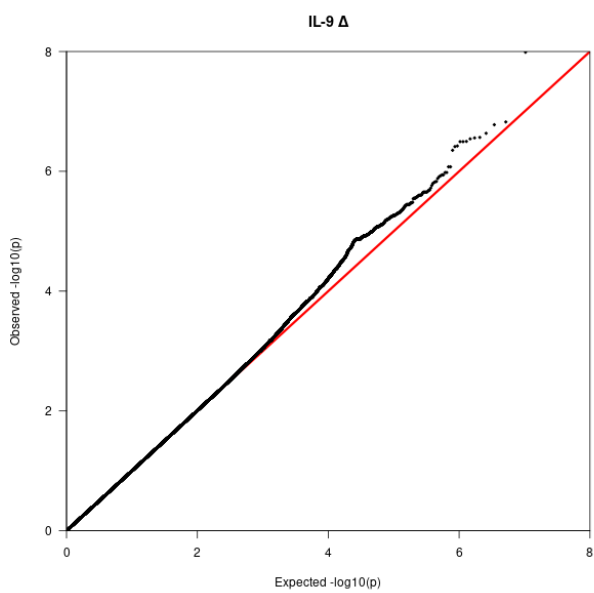

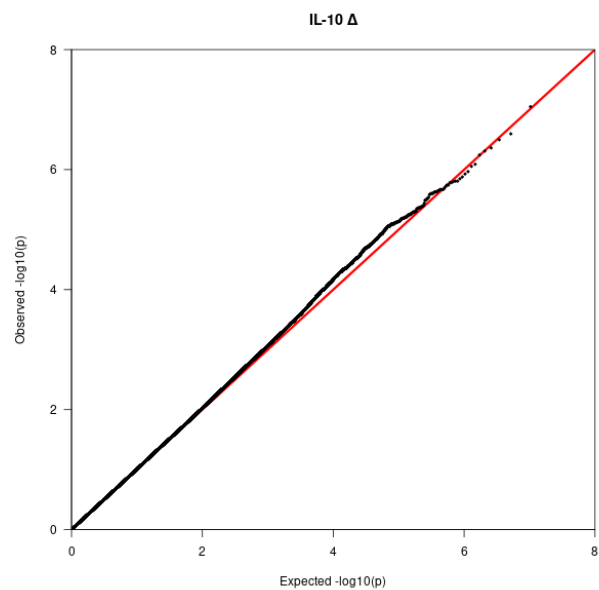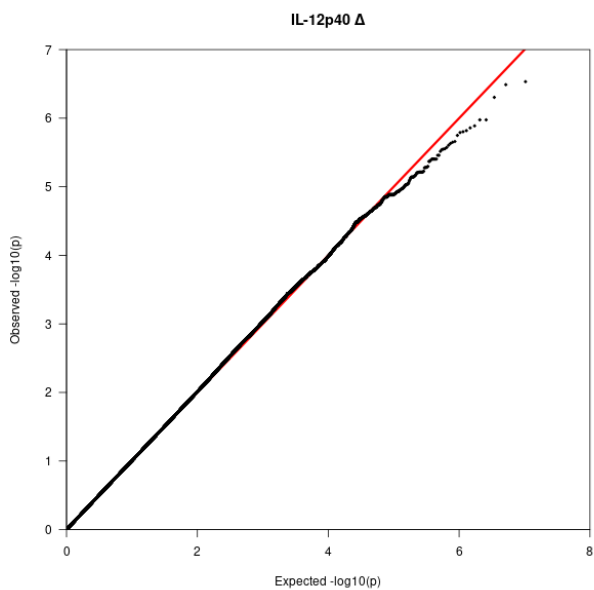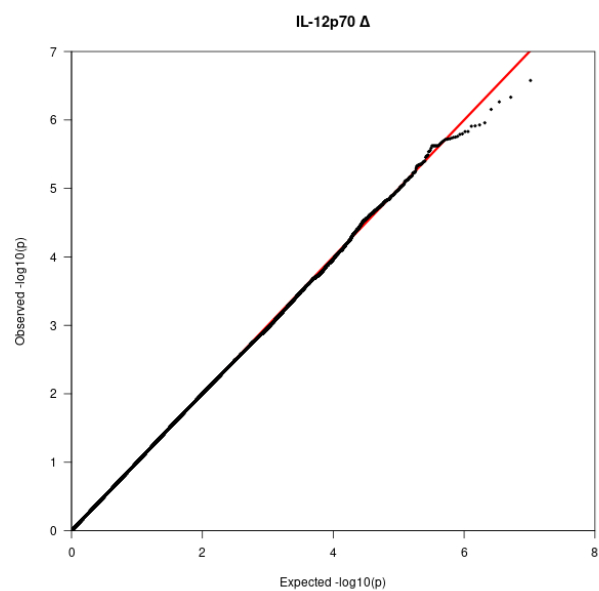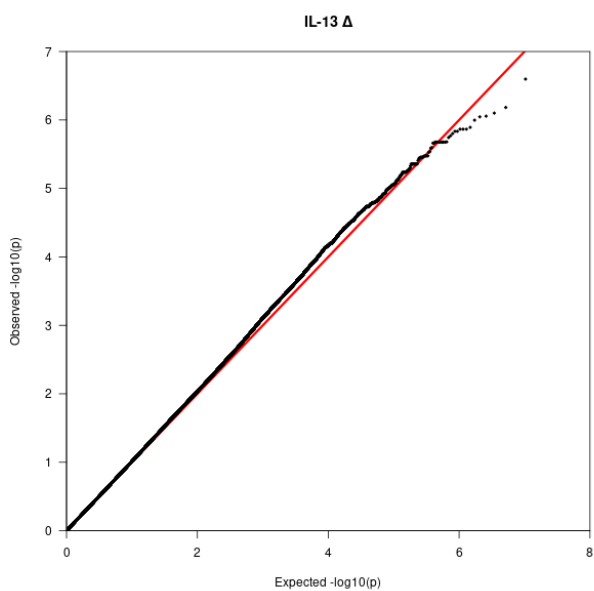

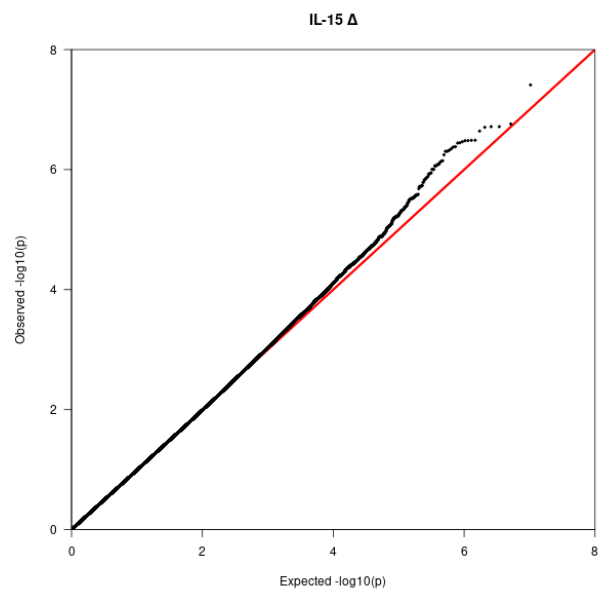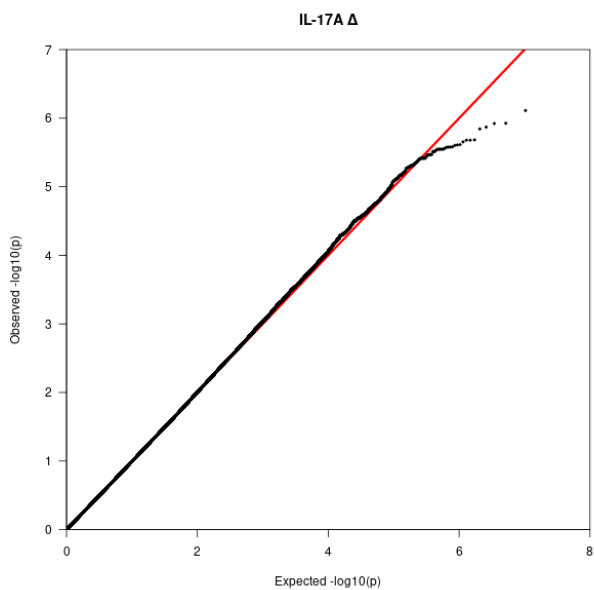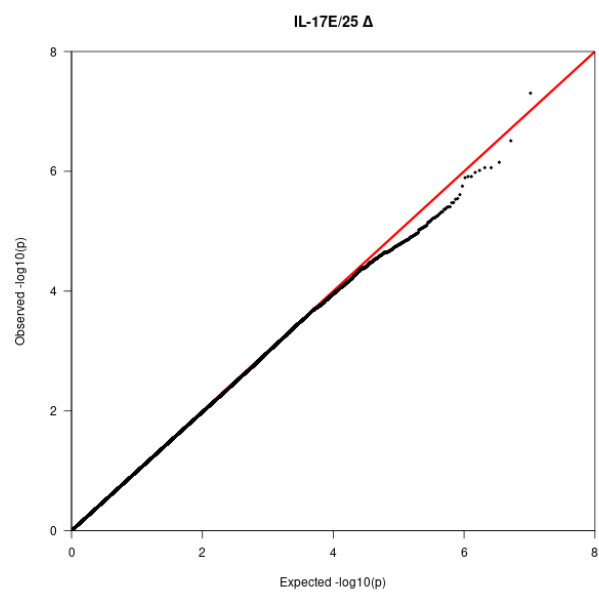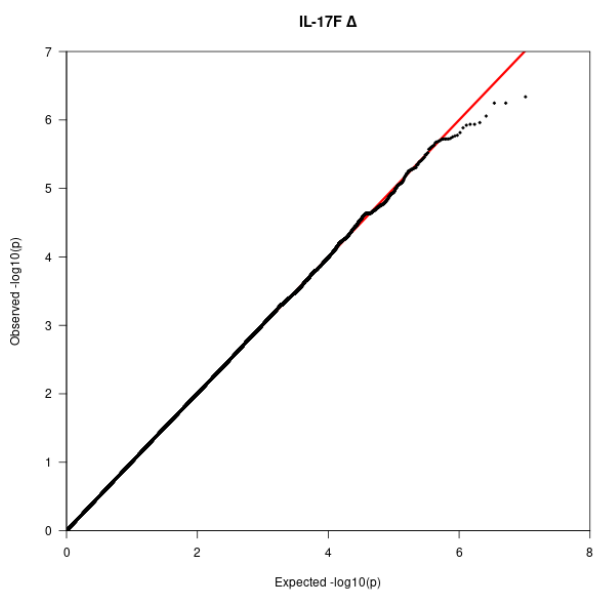

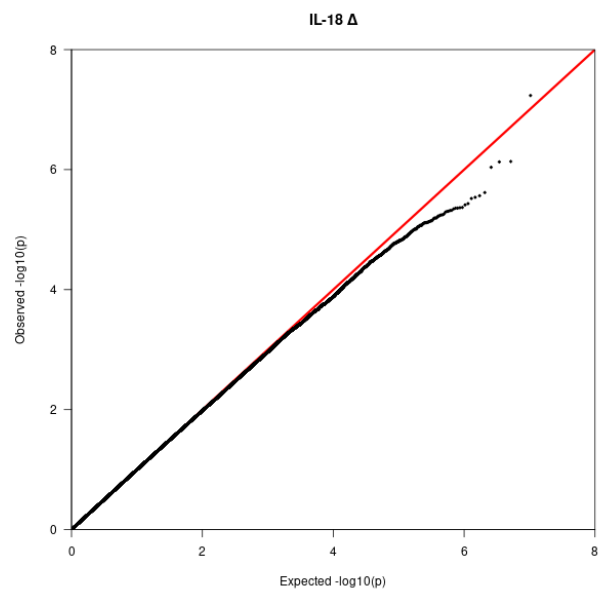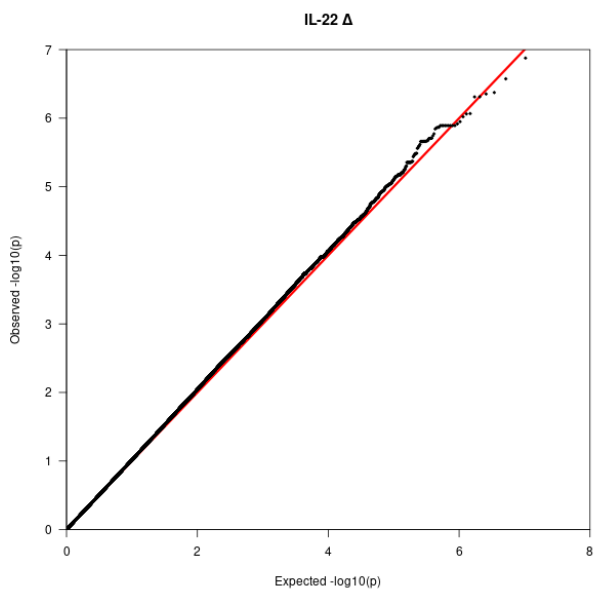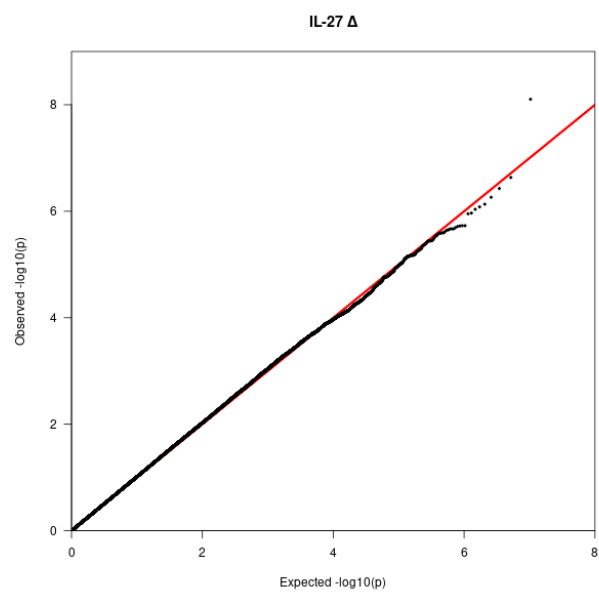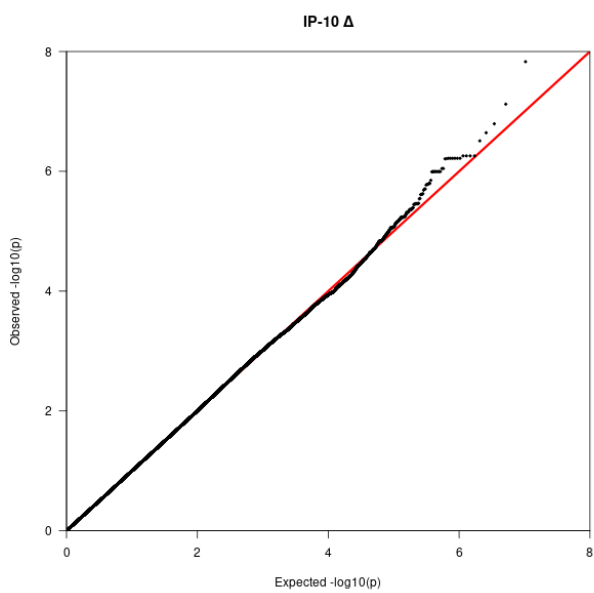

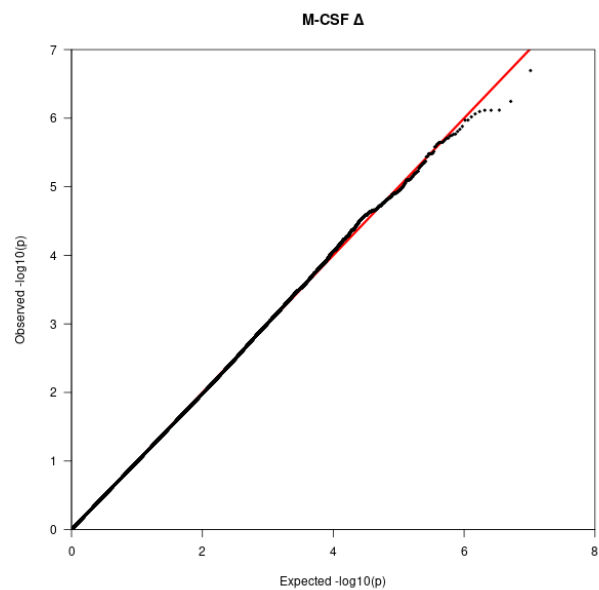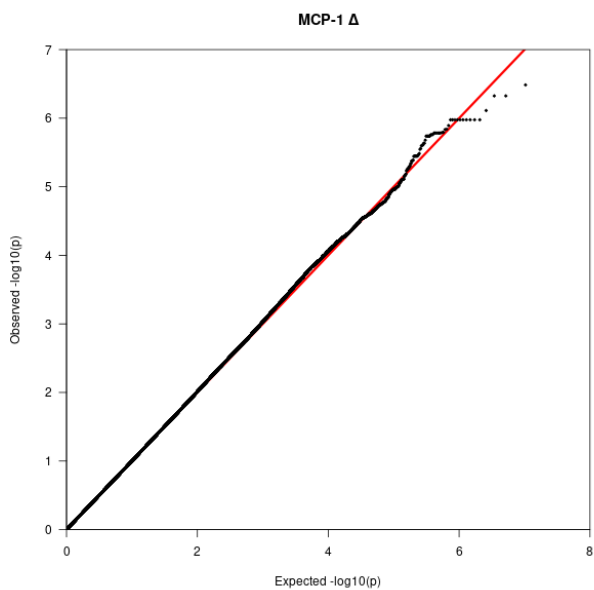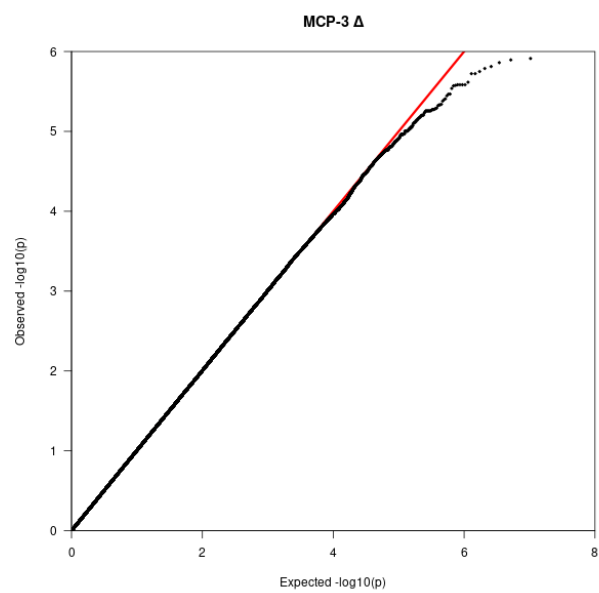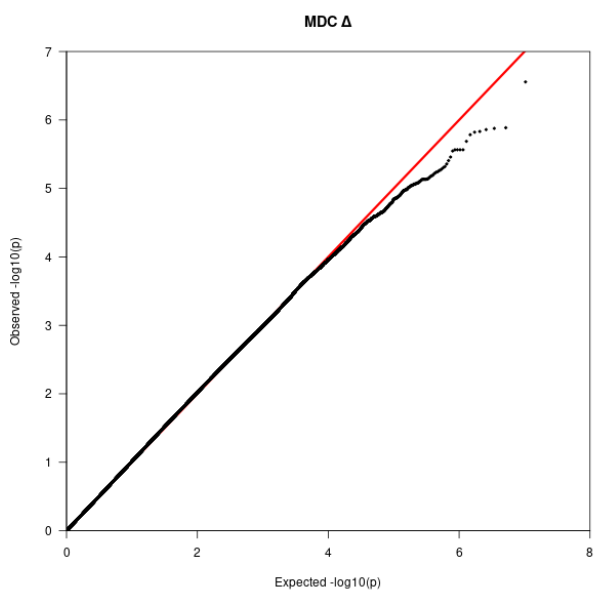

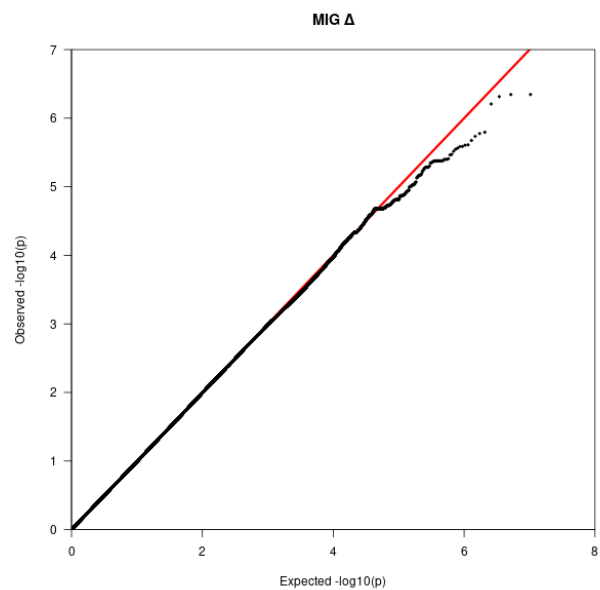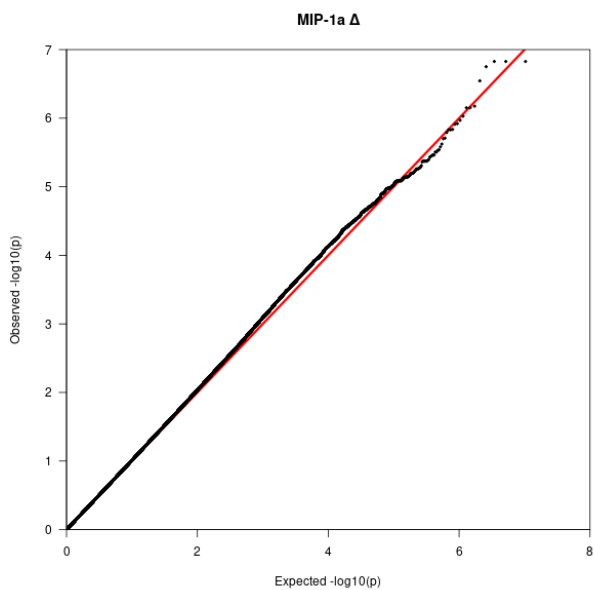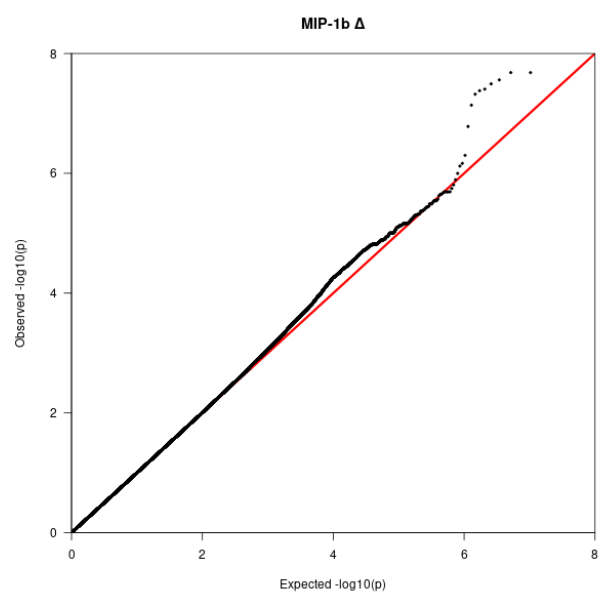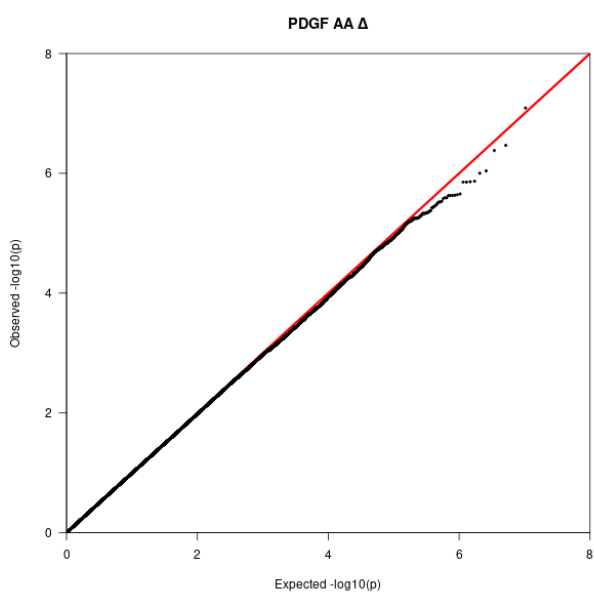

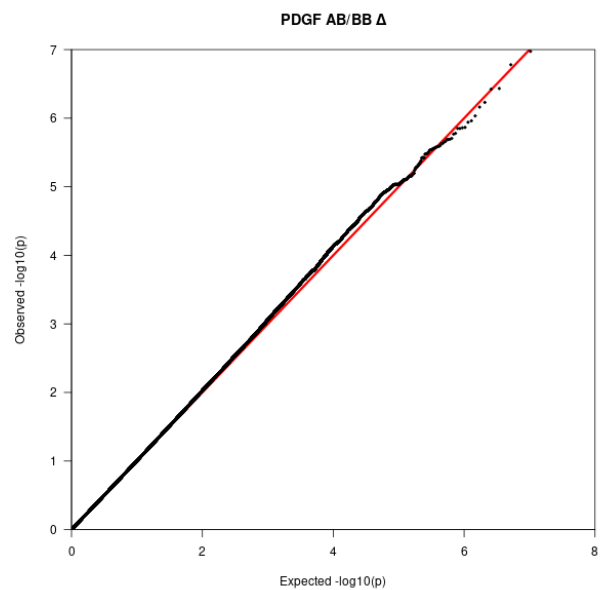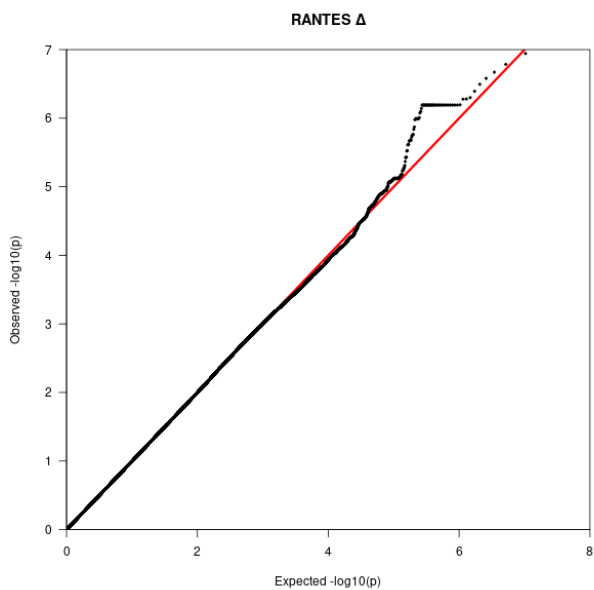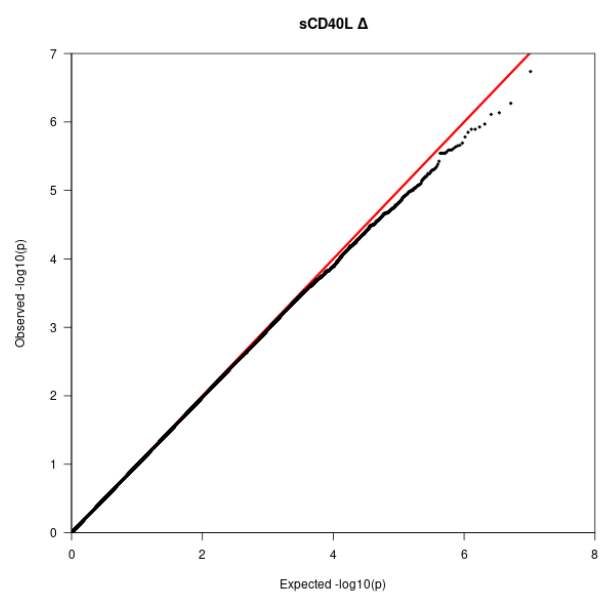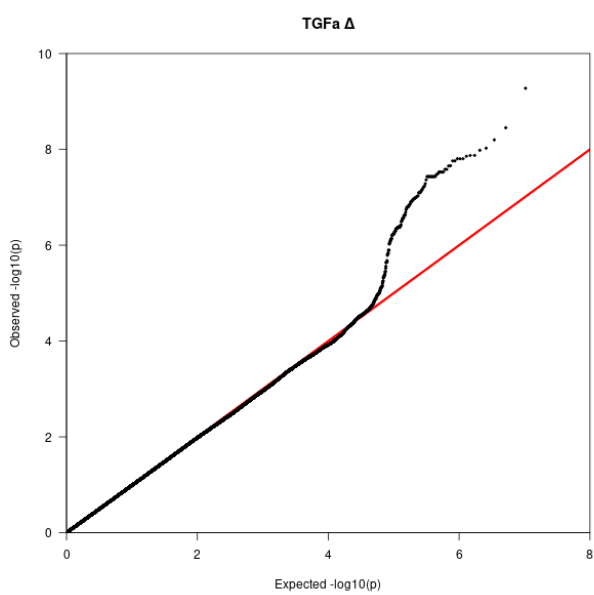

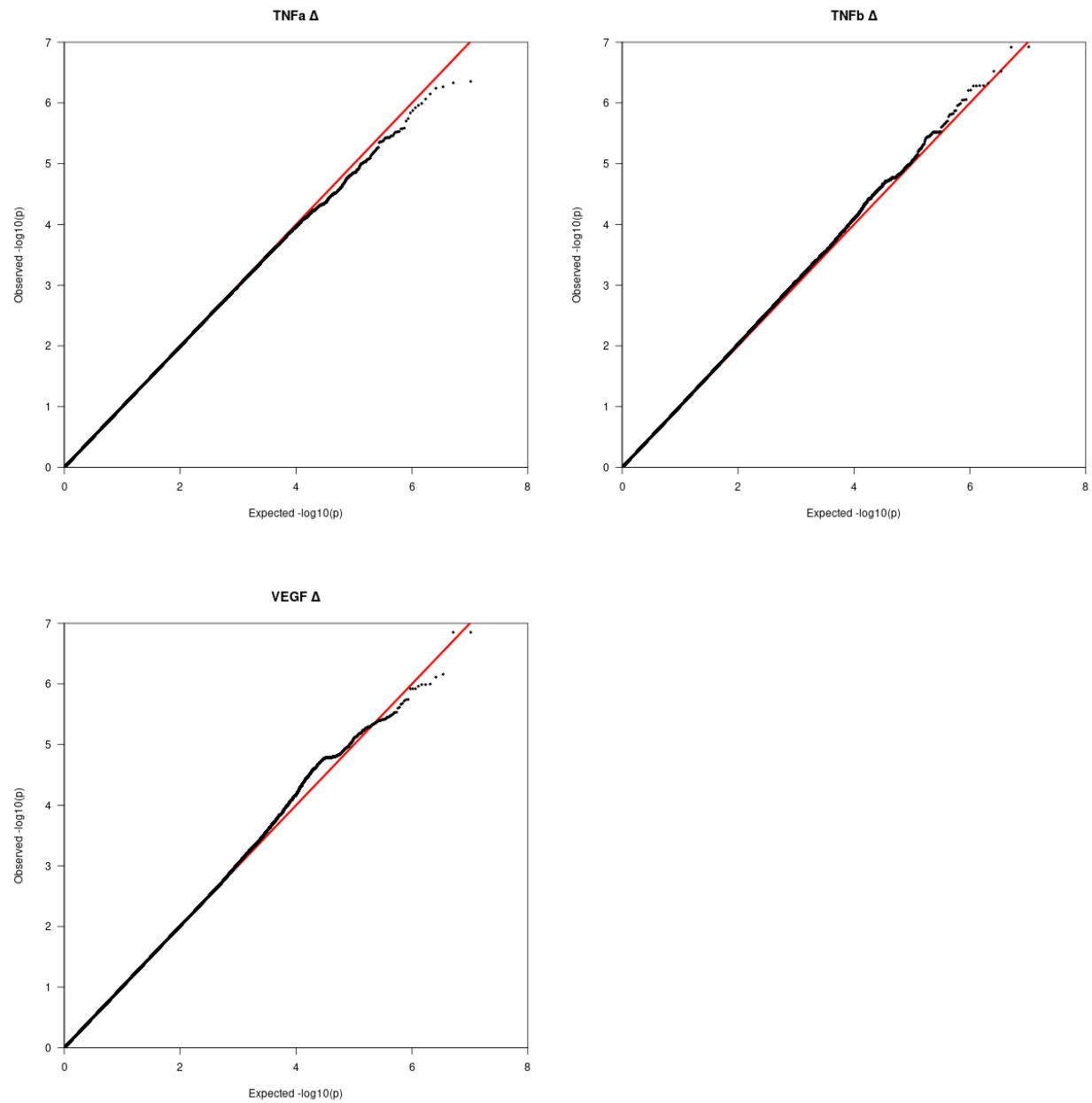

**S9 Fig. QQ-plots for all 141 GWAS.** QQ-plots for each T1 (a), T2 (b), and  $\Delta$  (c) GWAS.

Supplement: S9 Fig — QQ-plots for each T1 (a), T2 (b), and Δ (c) GWAS. (PDF) [file pgen.1012204.s011.pdf]

**a** EGF T1

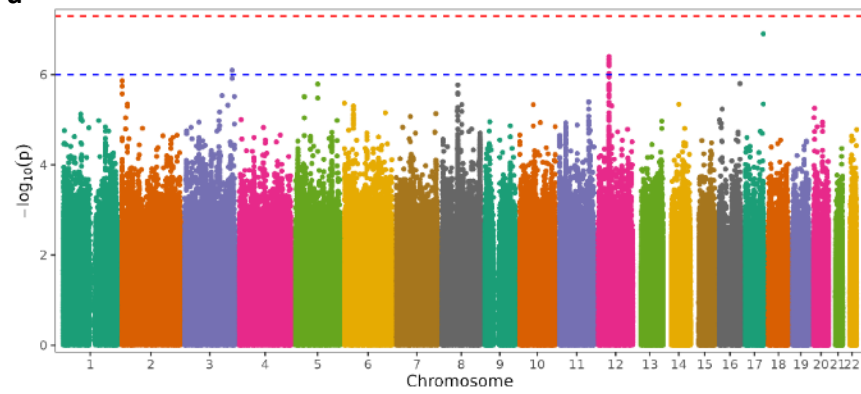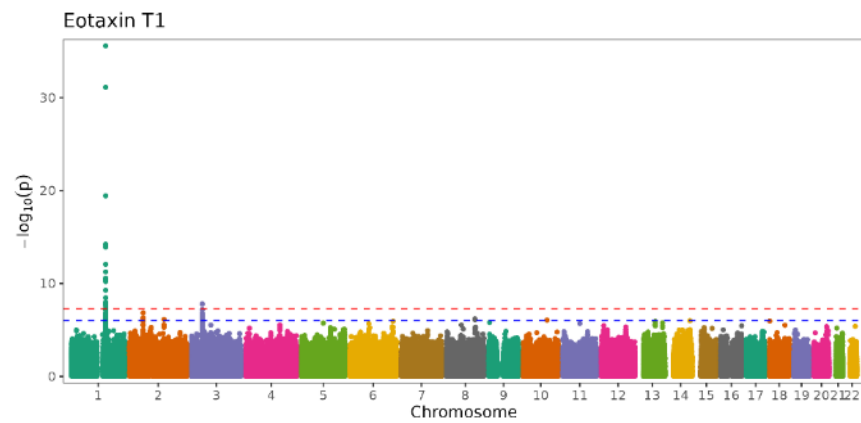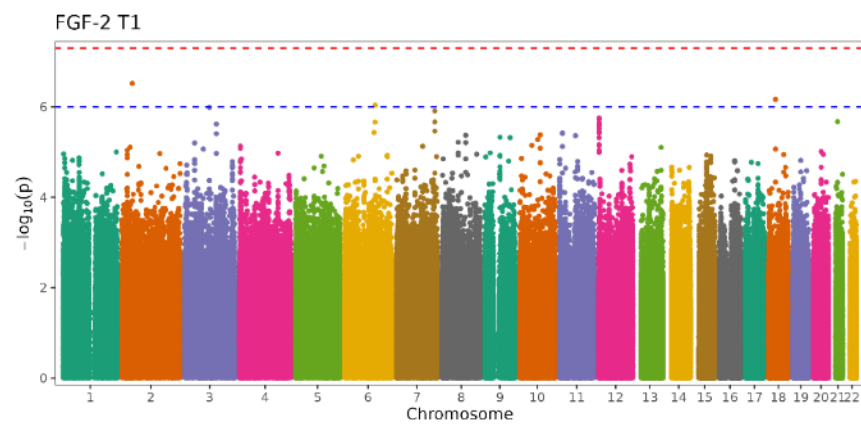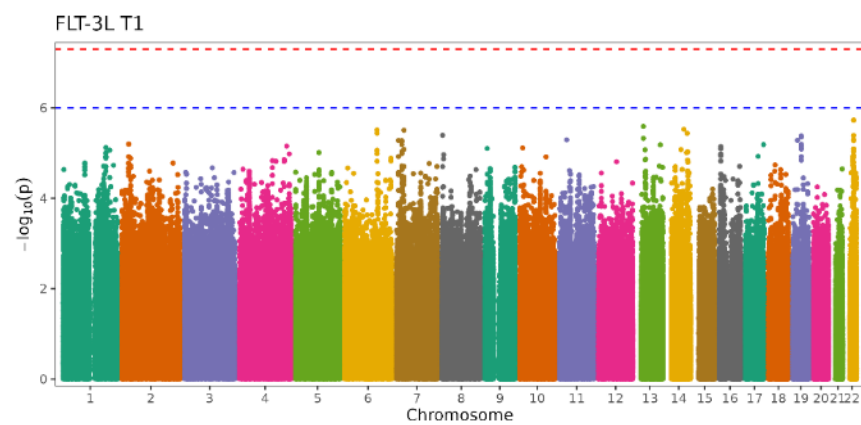

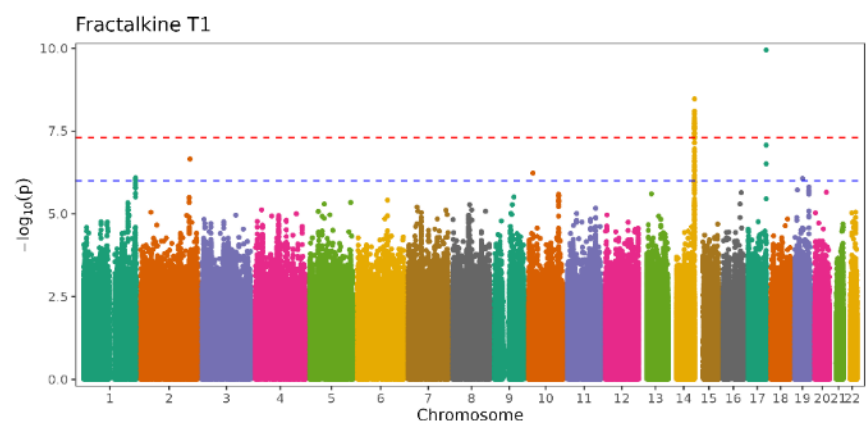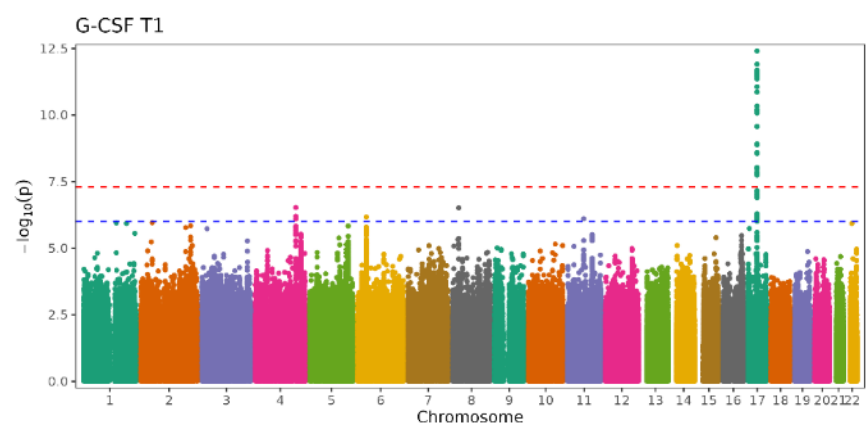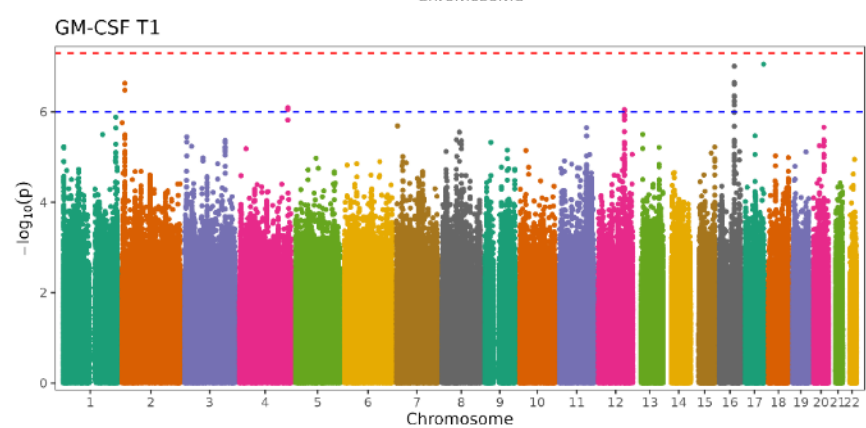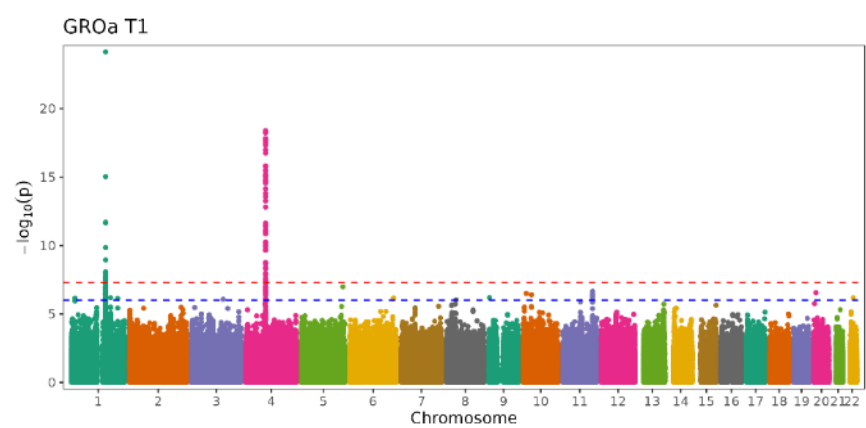

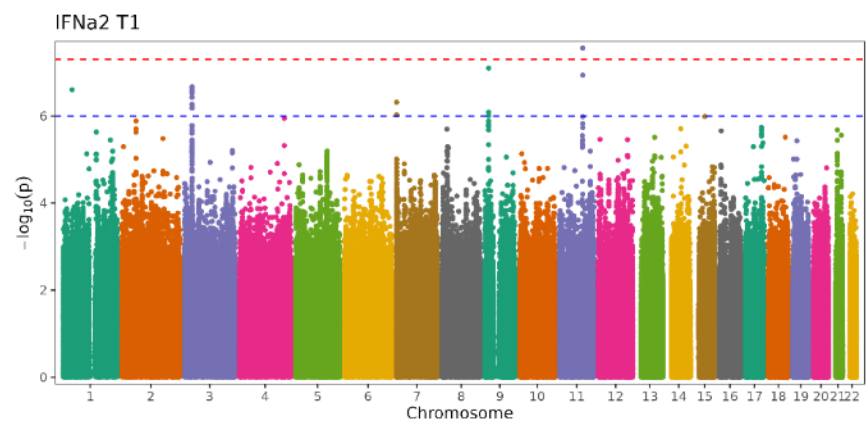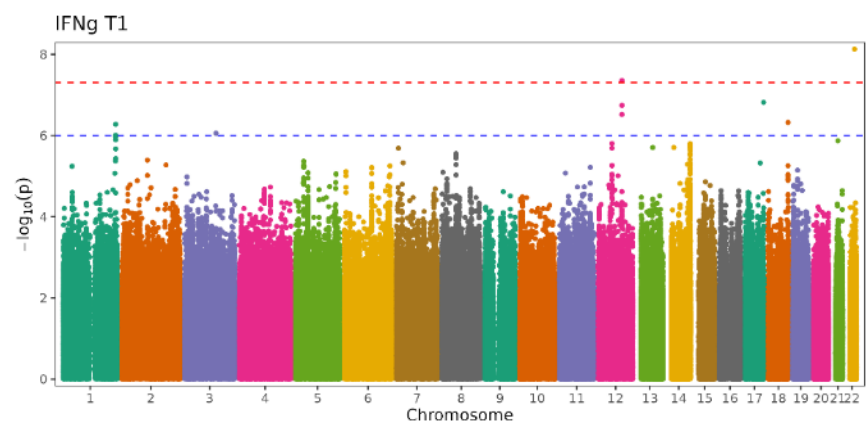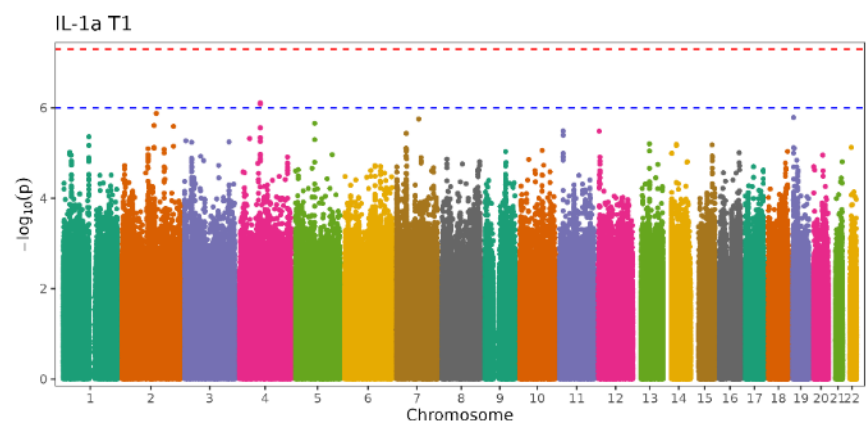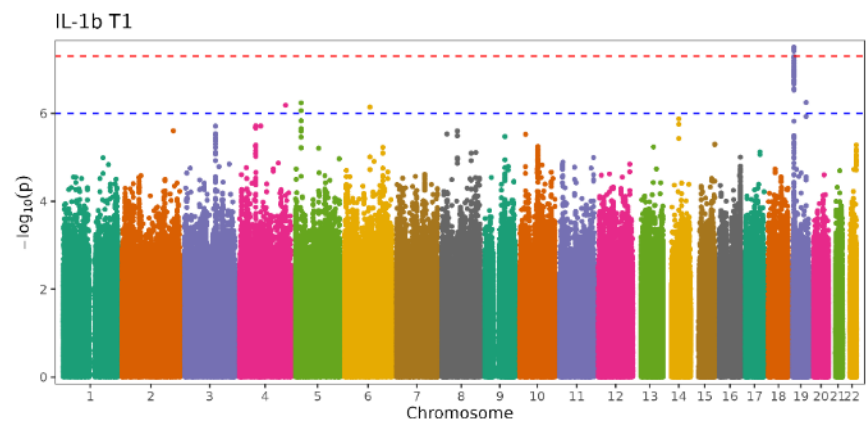

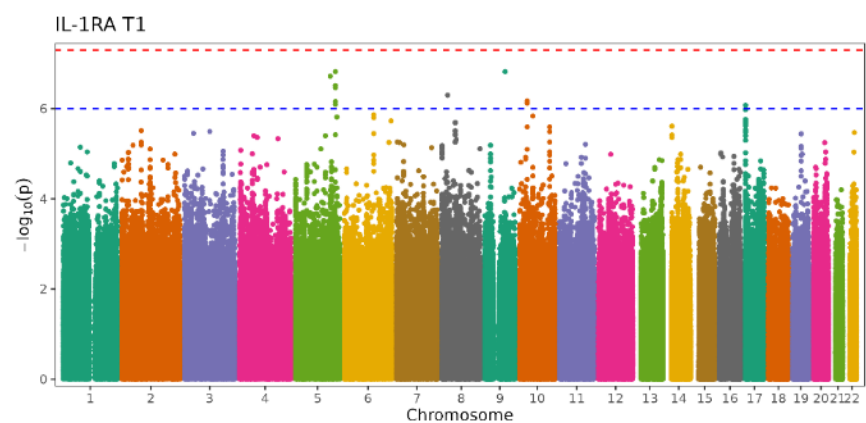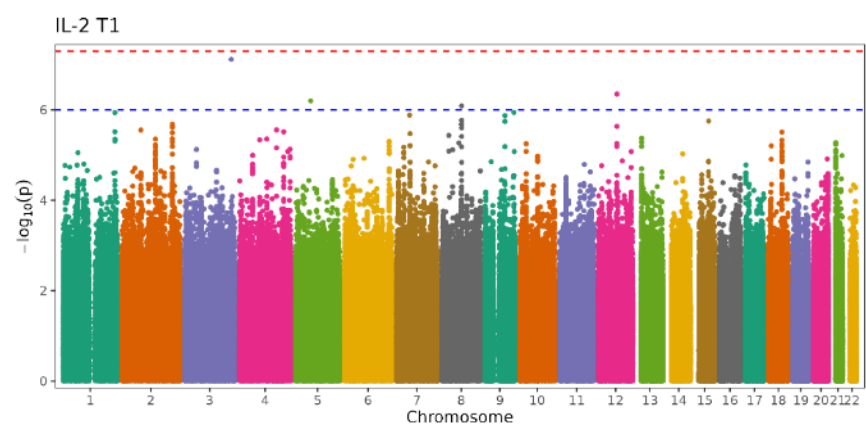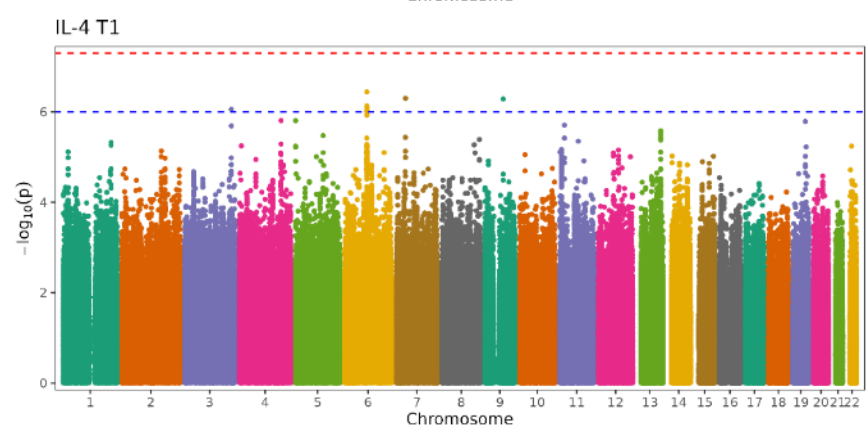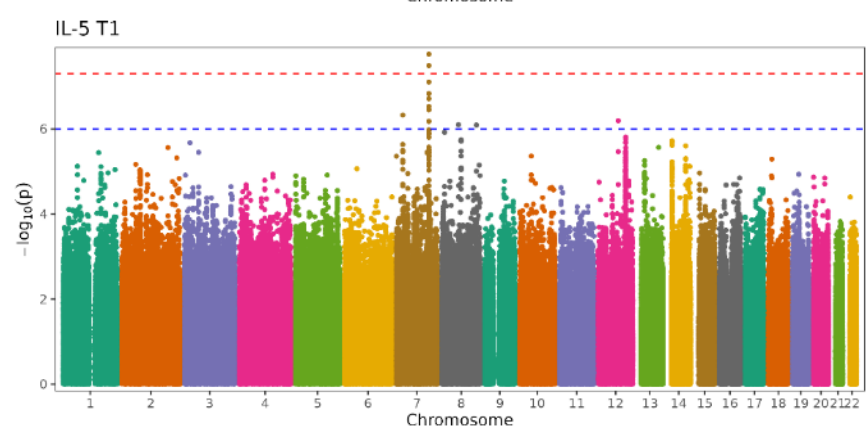

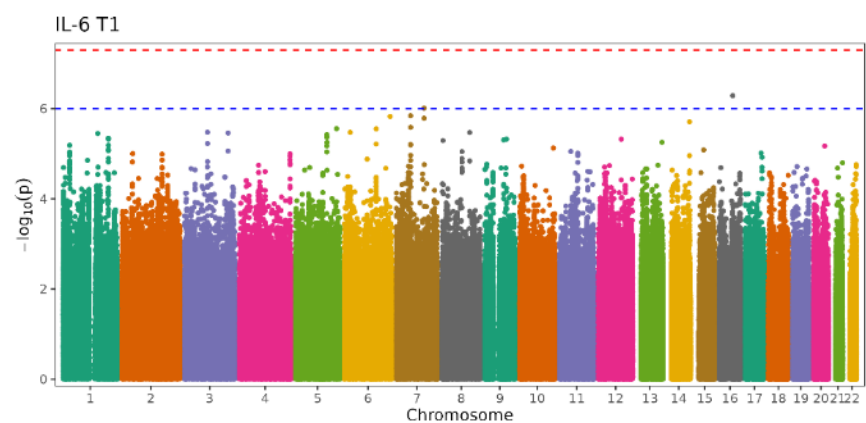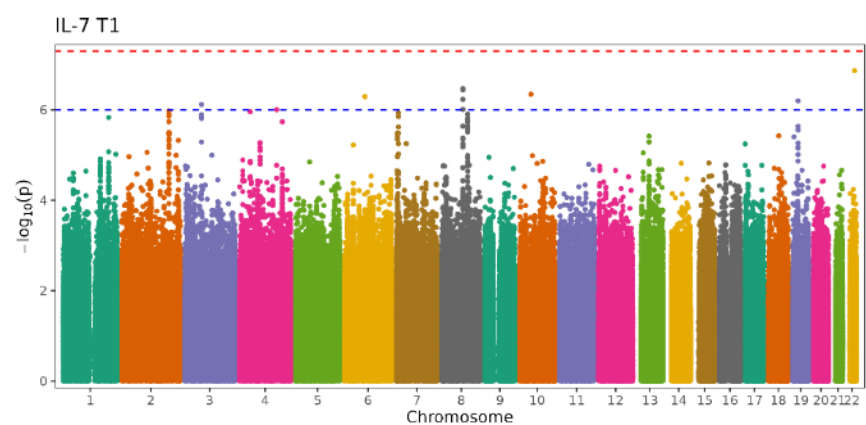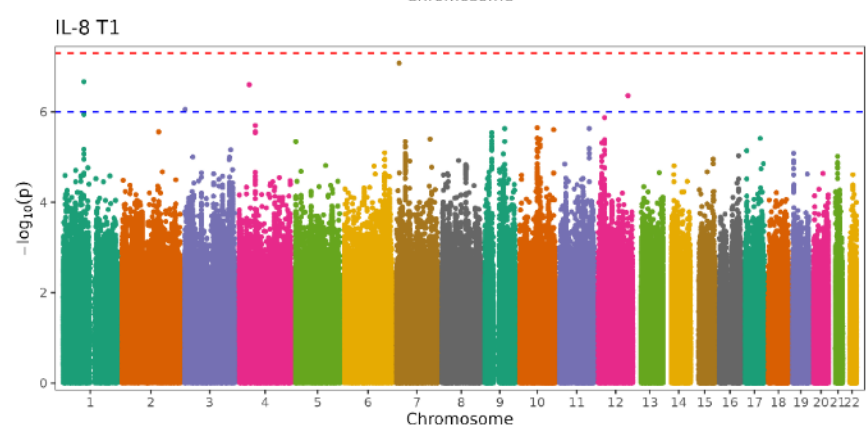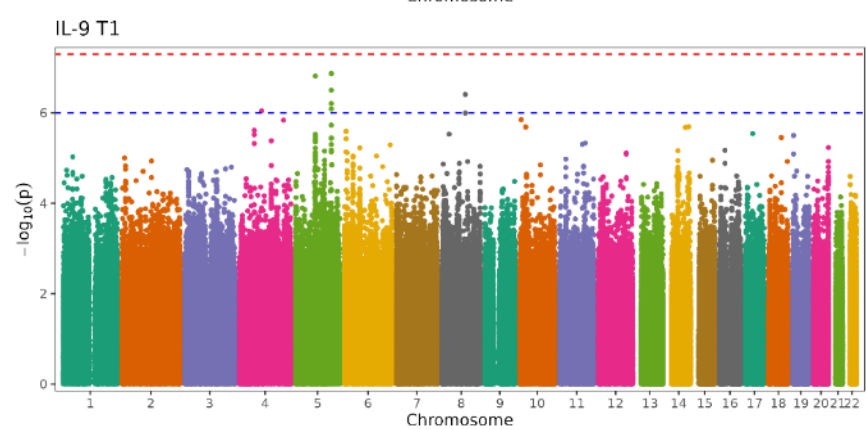

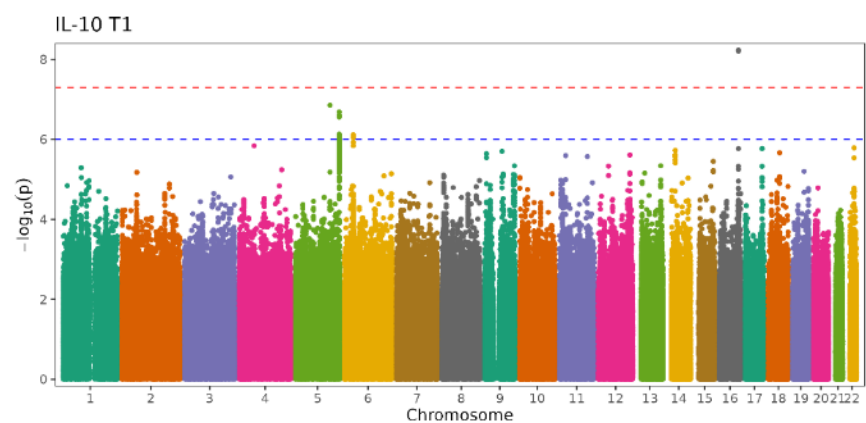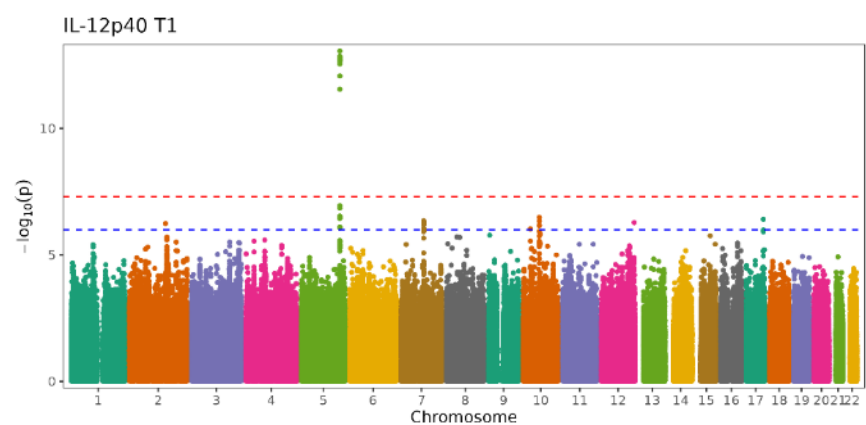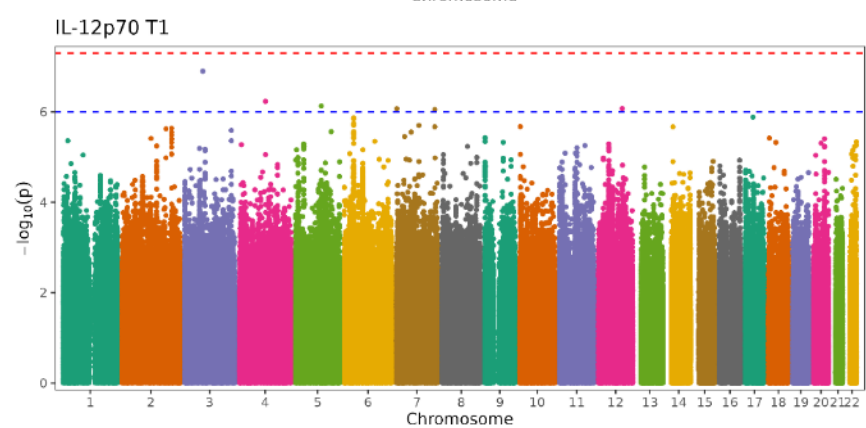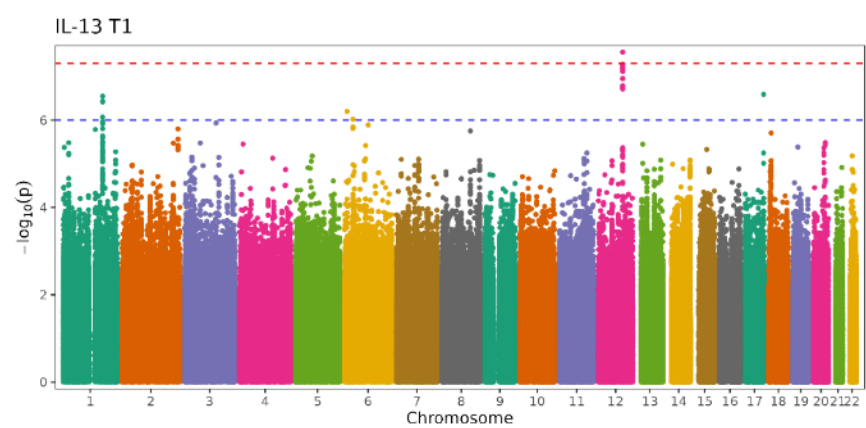

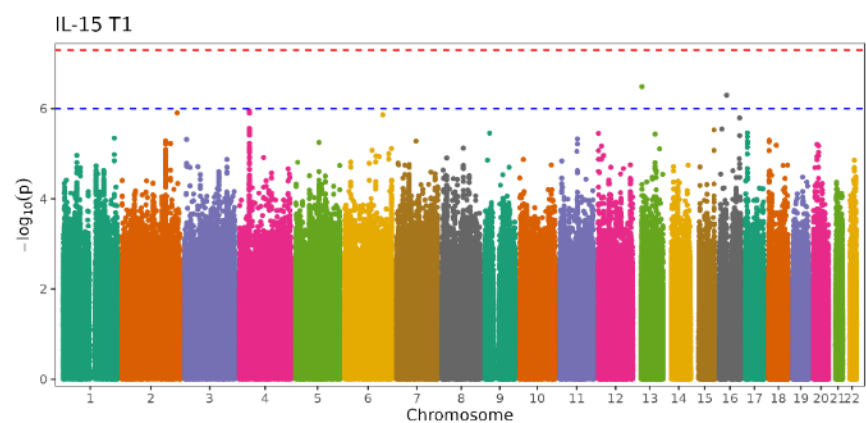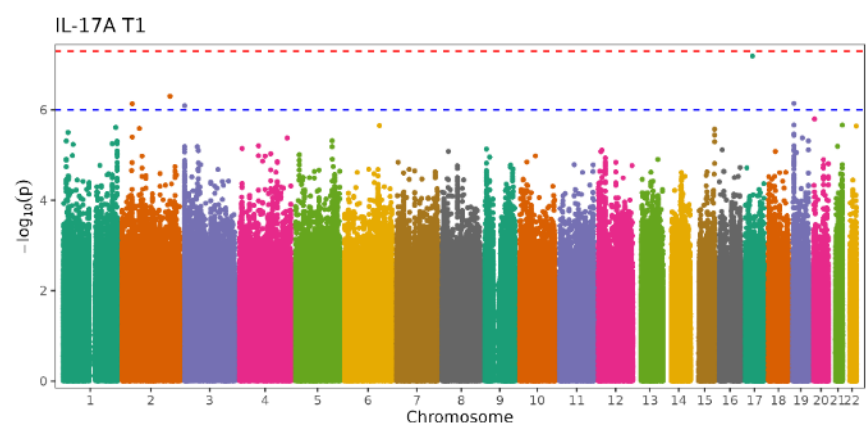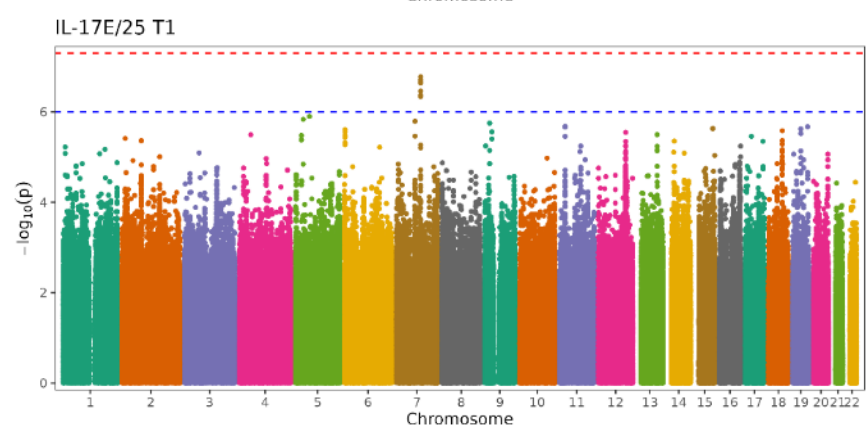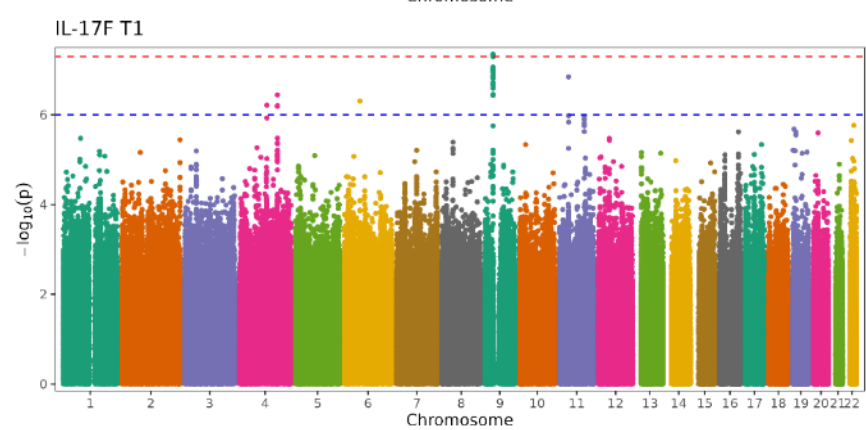

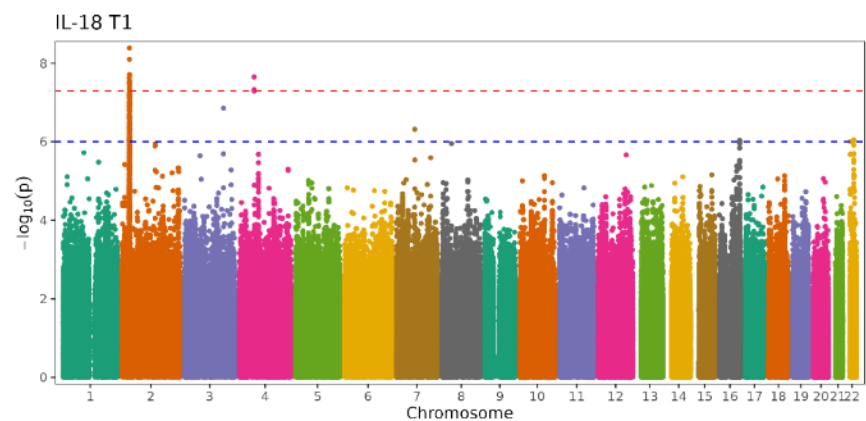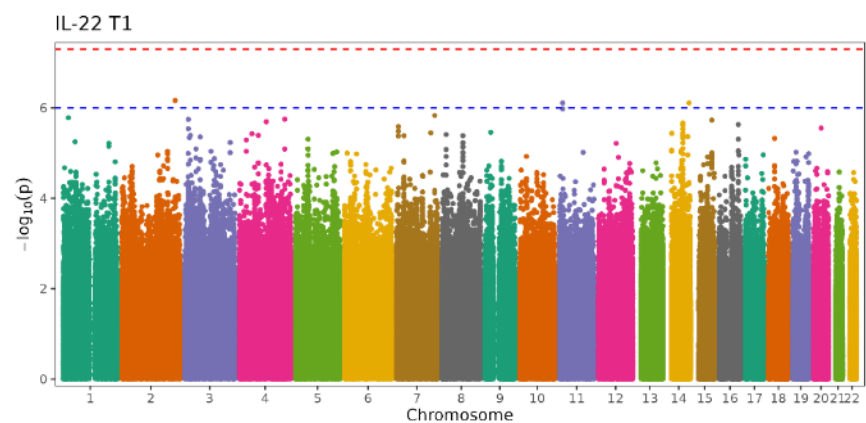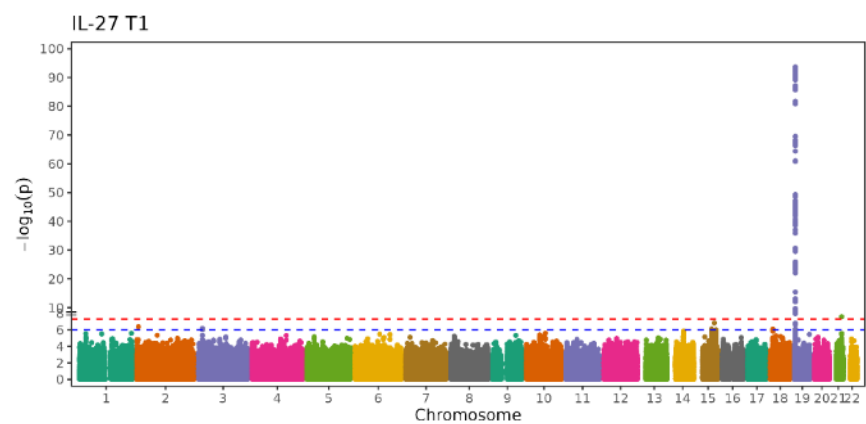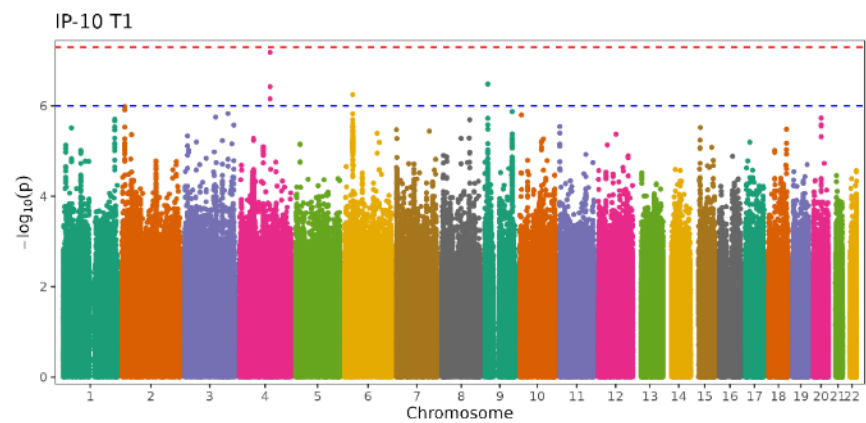

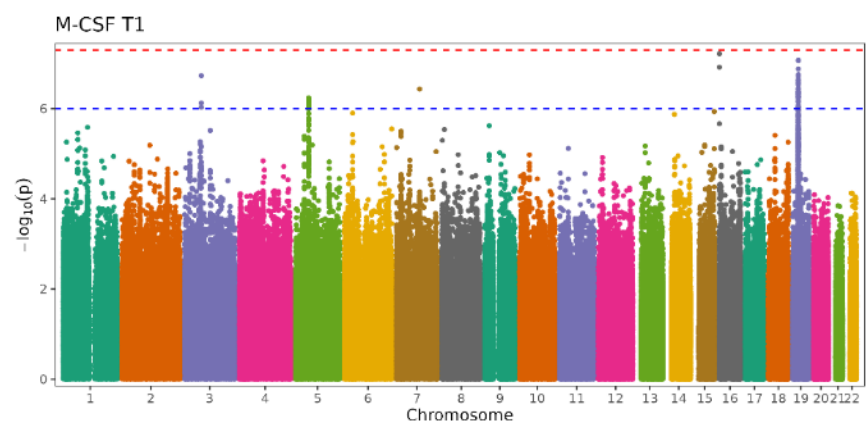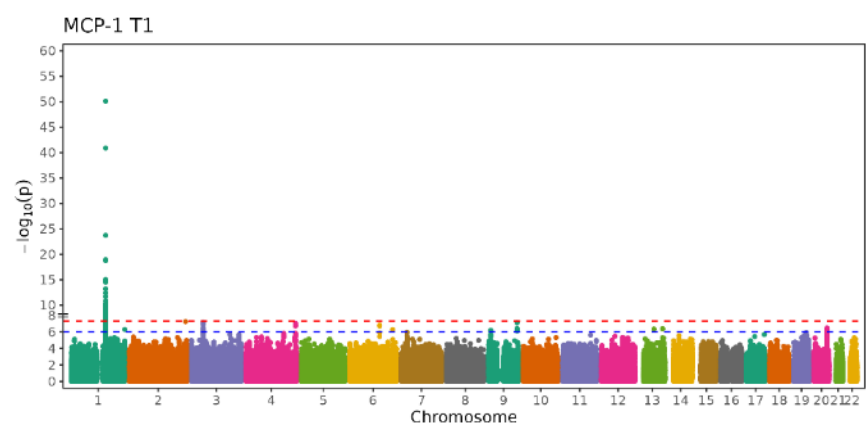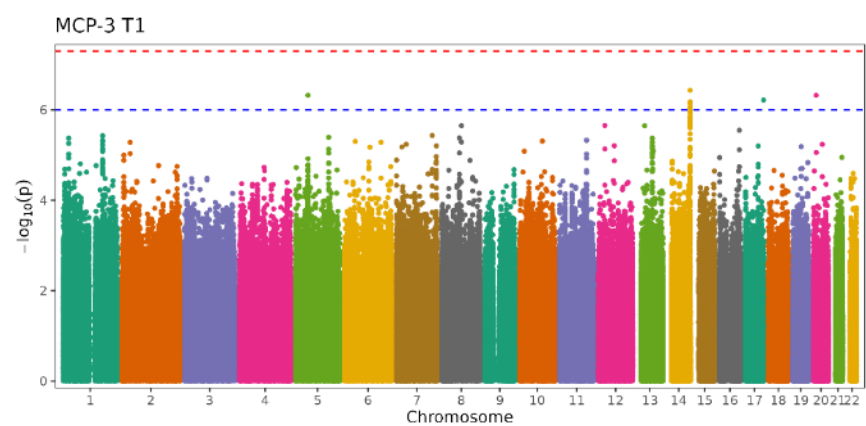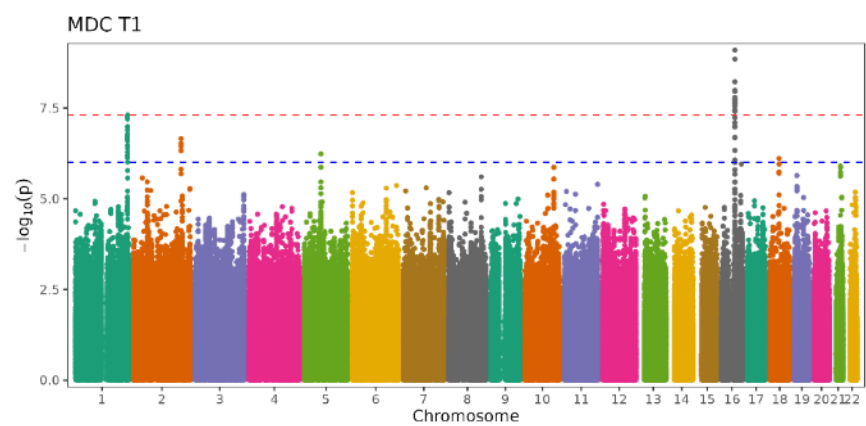

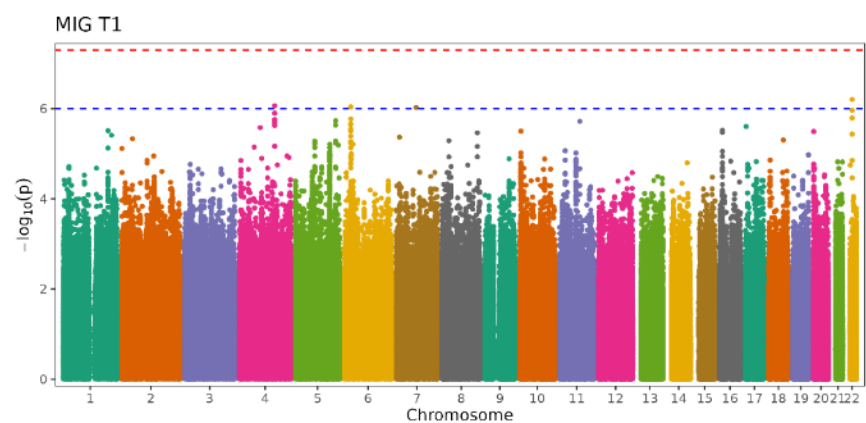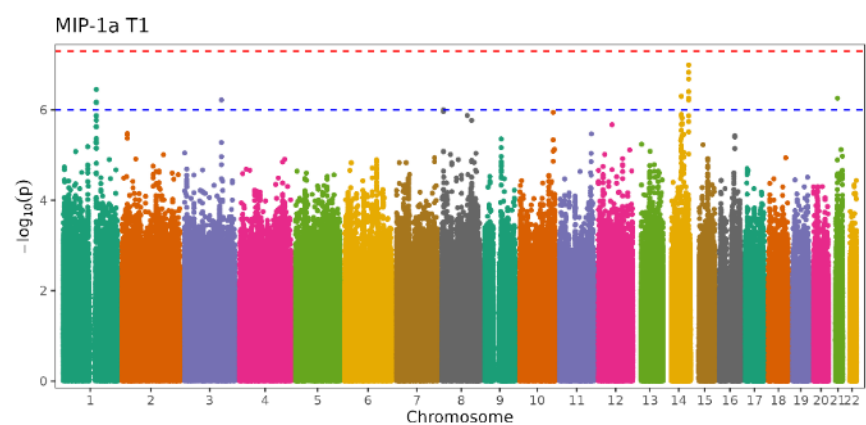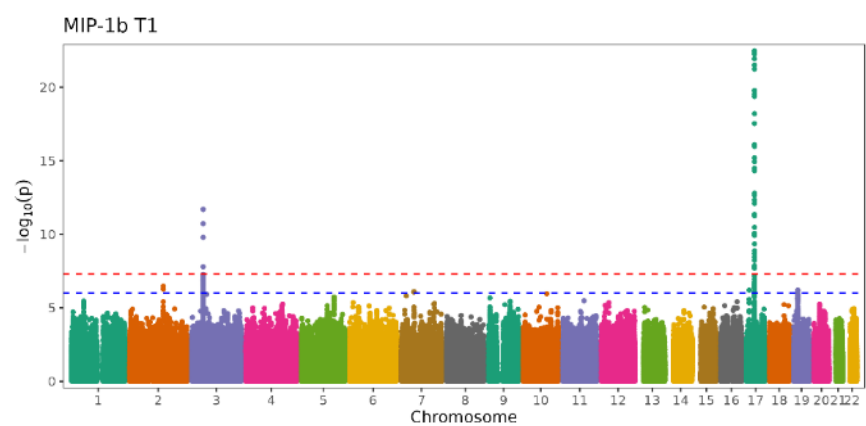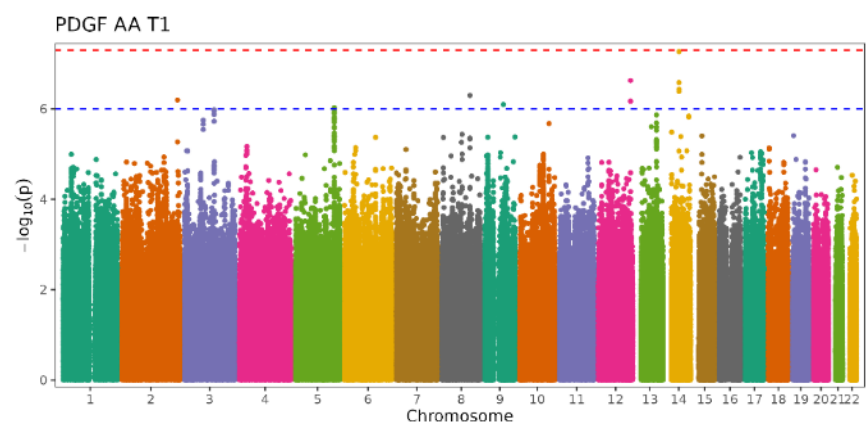

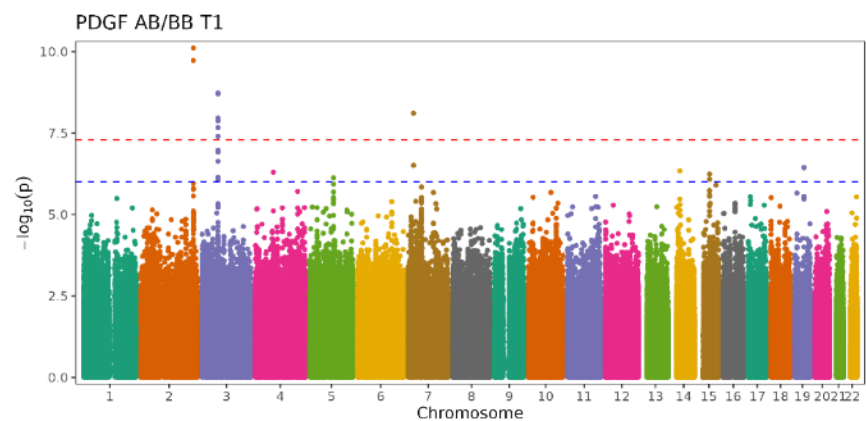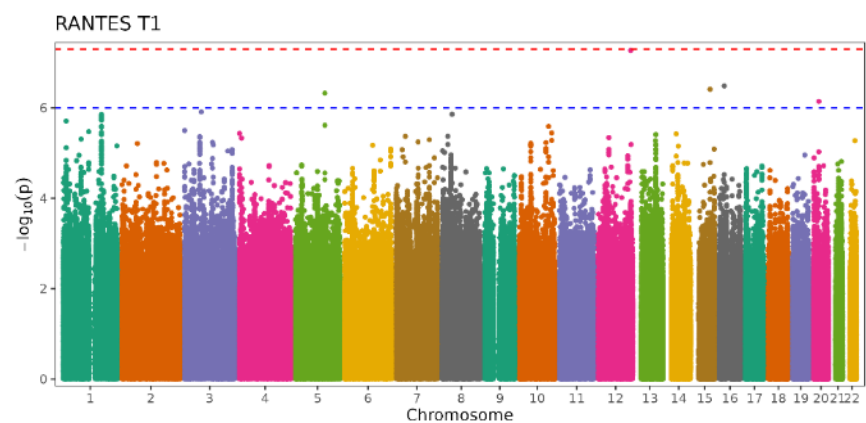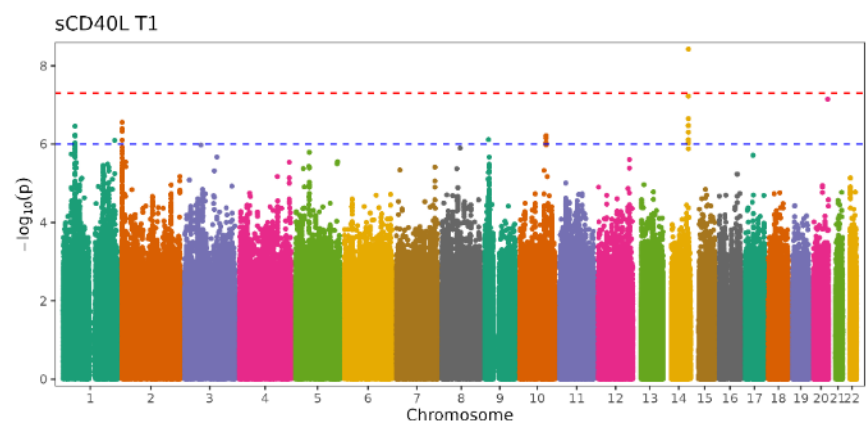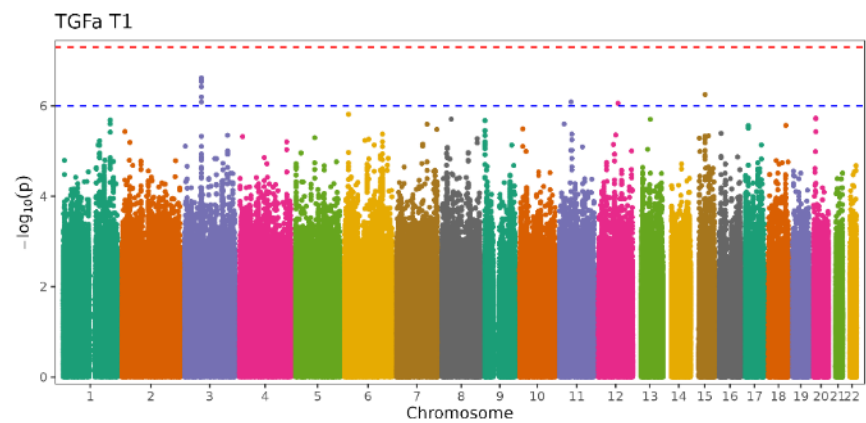

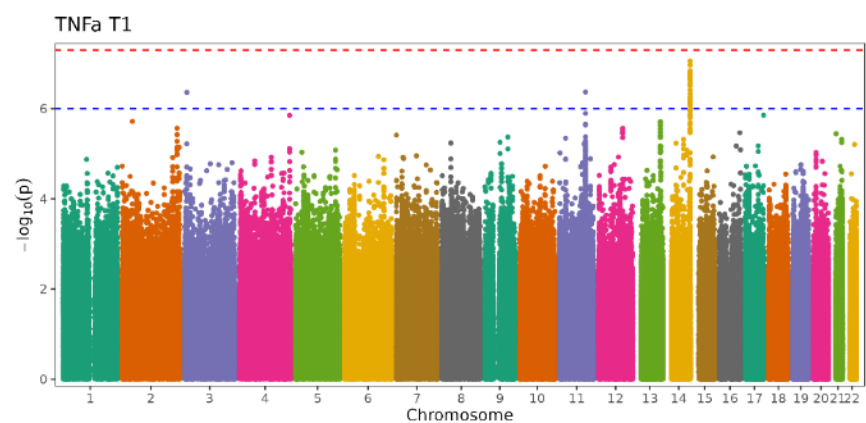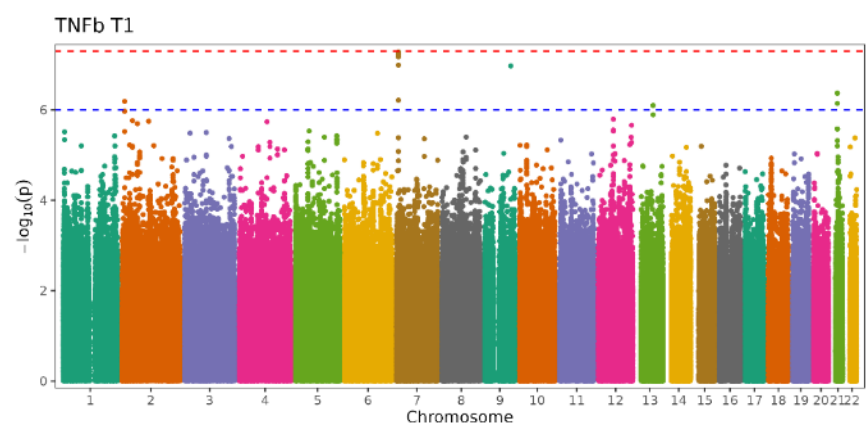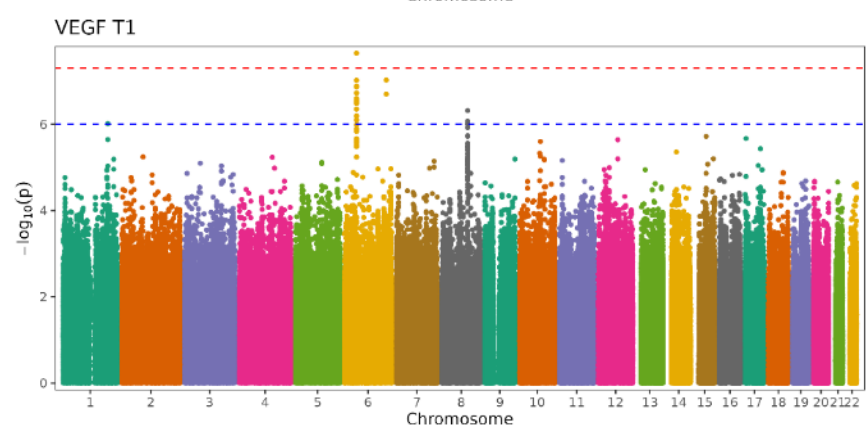

**b** EGF T2

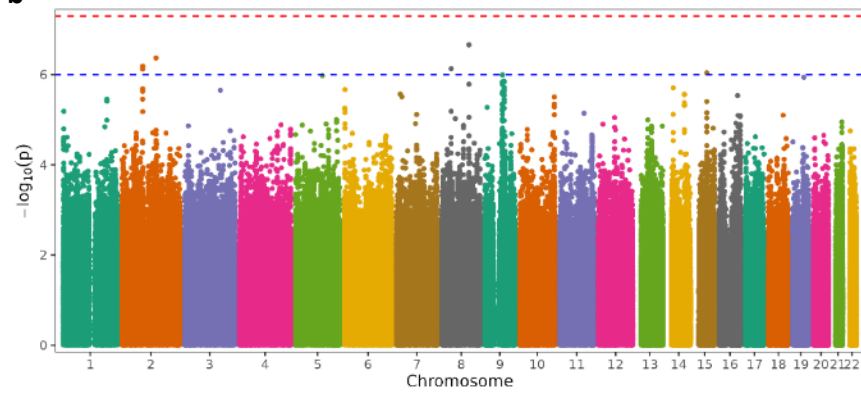

Eotaxin T2

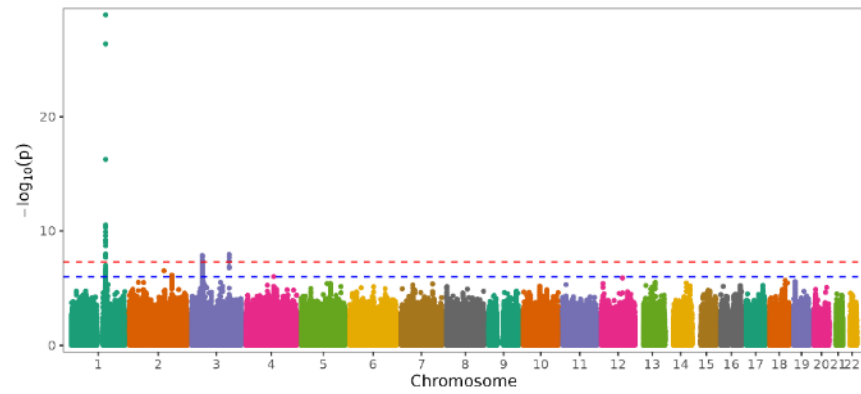

FGF-2 T2

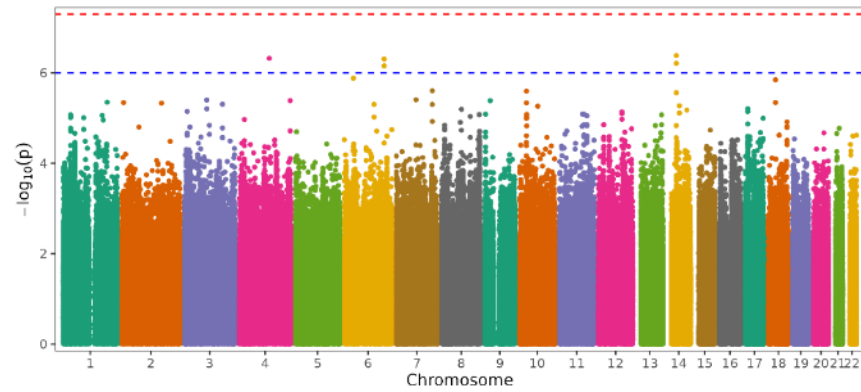

FLT-3L T2

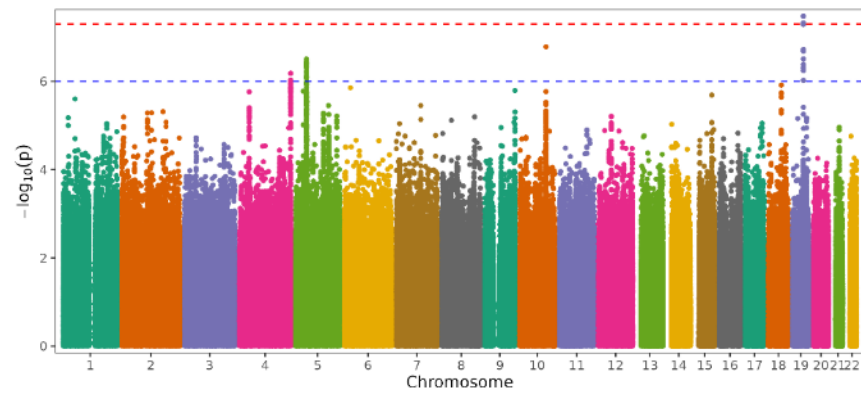

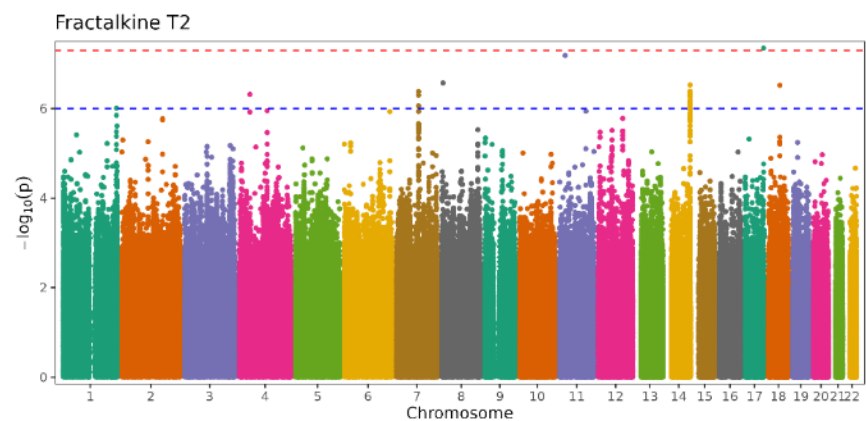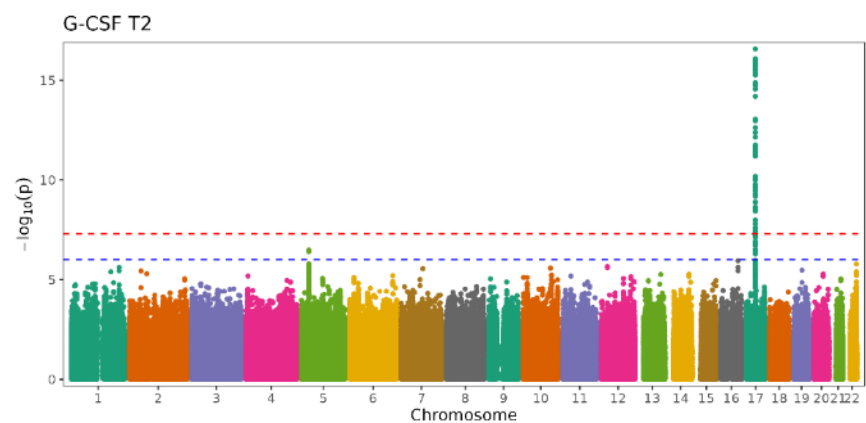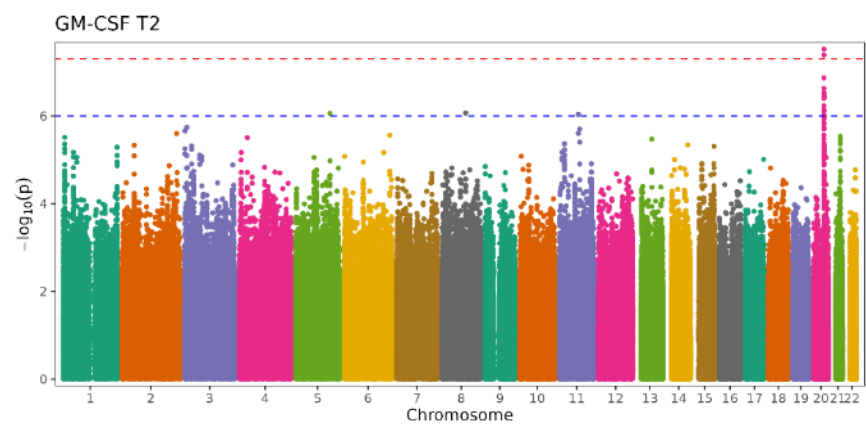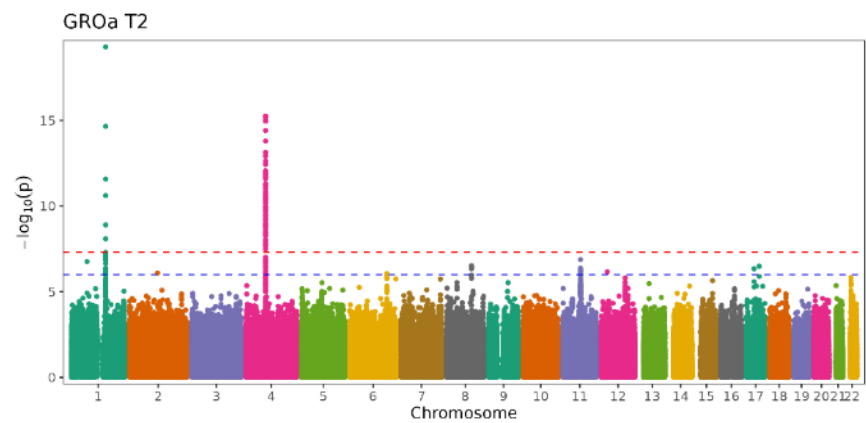

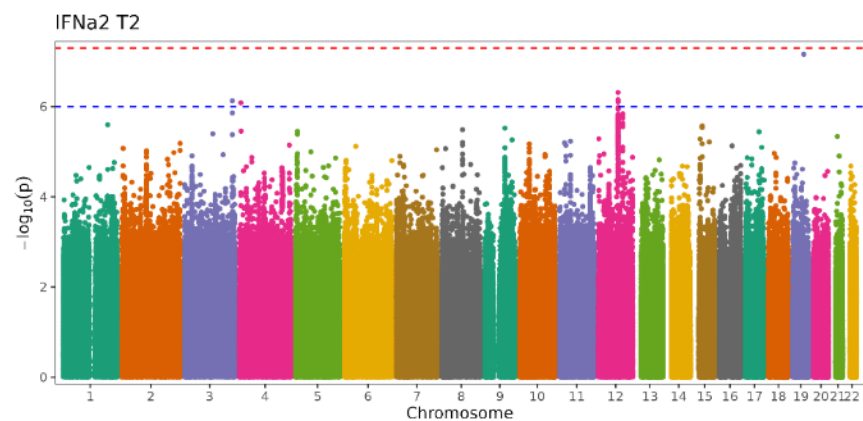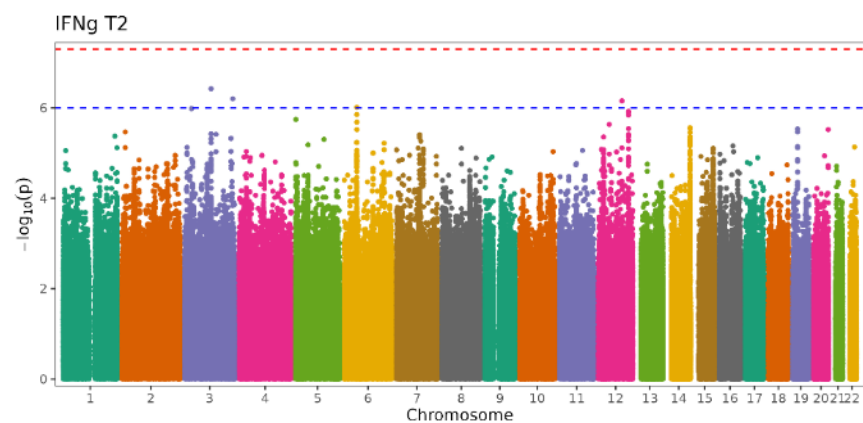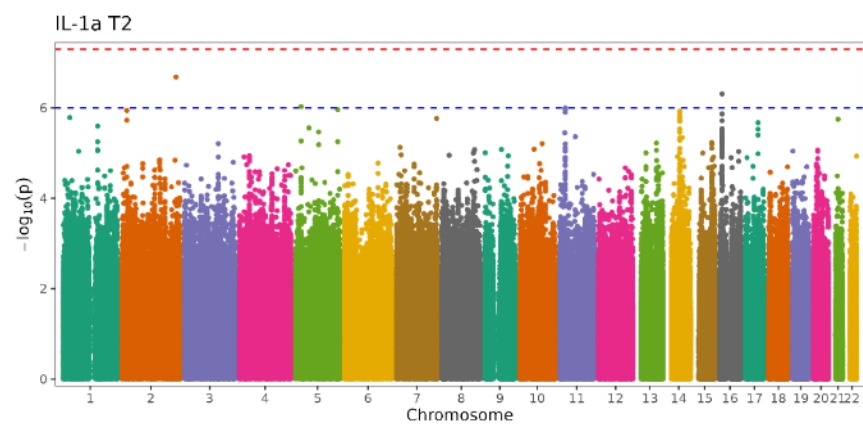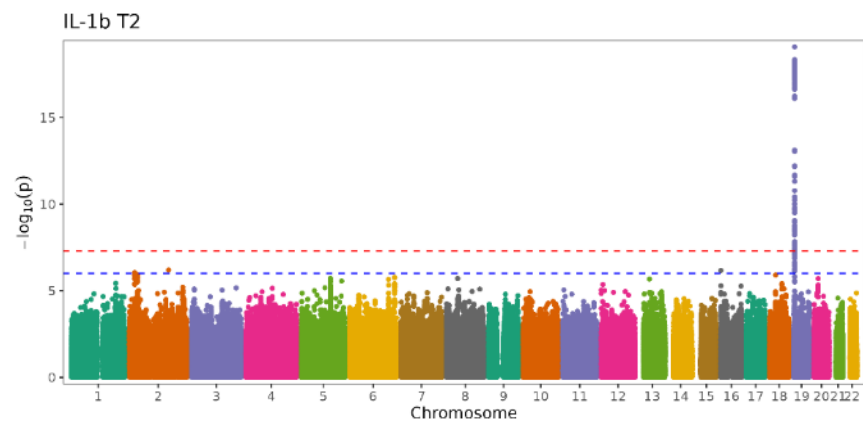

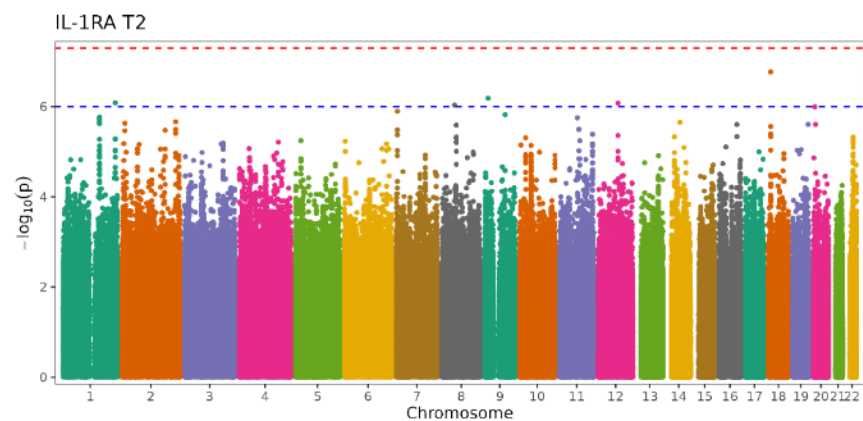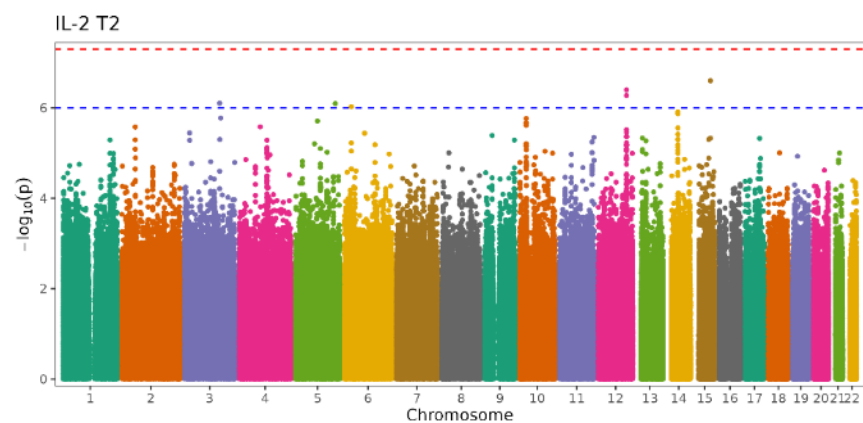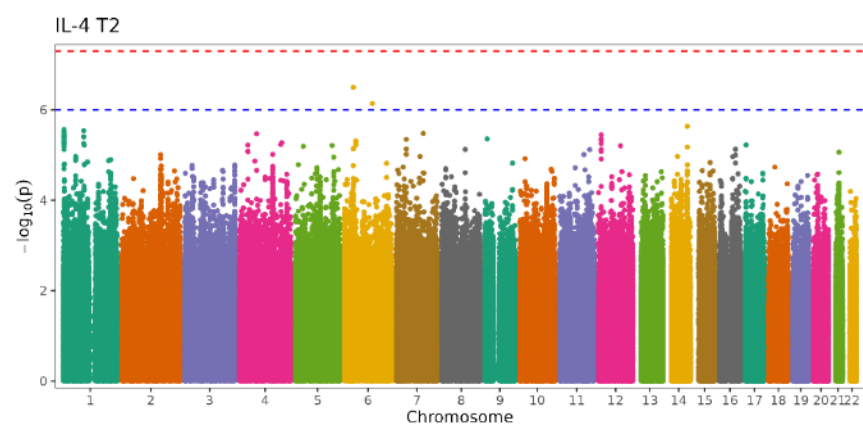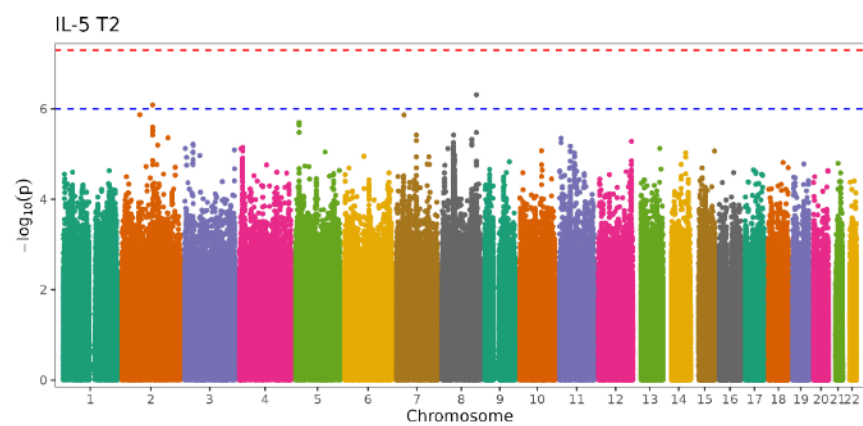

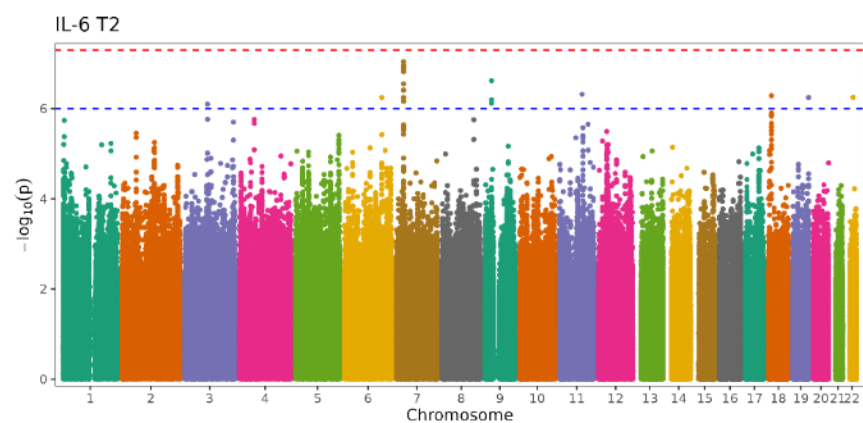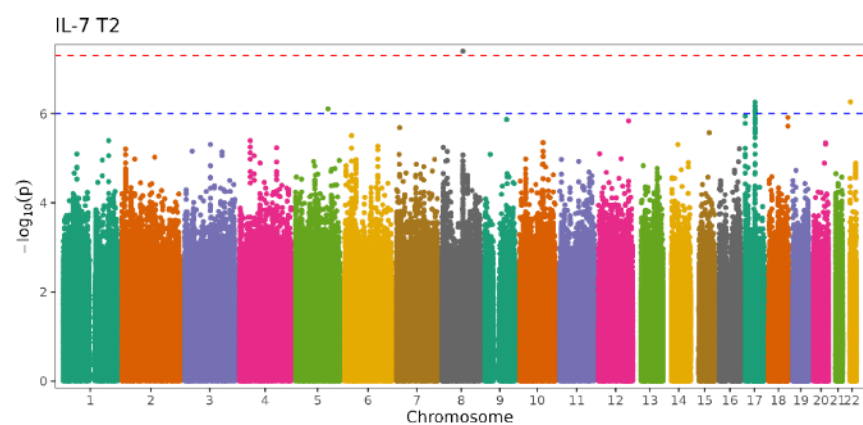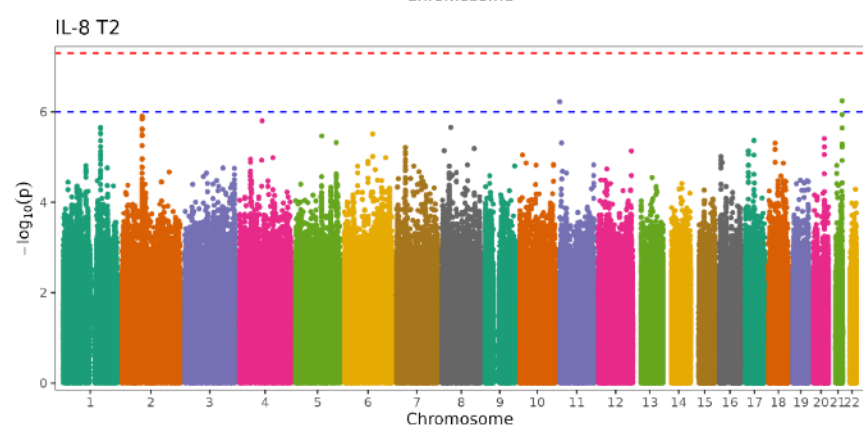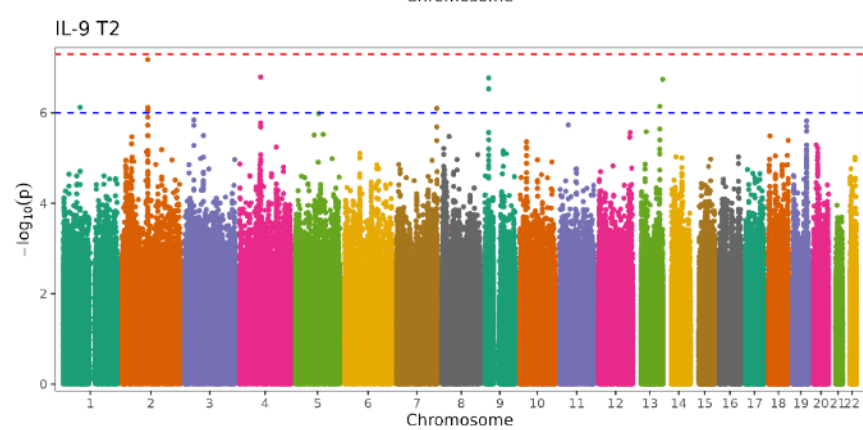

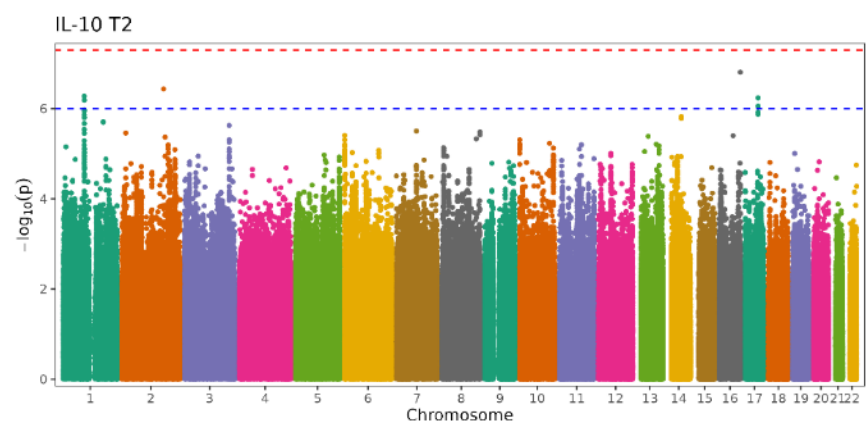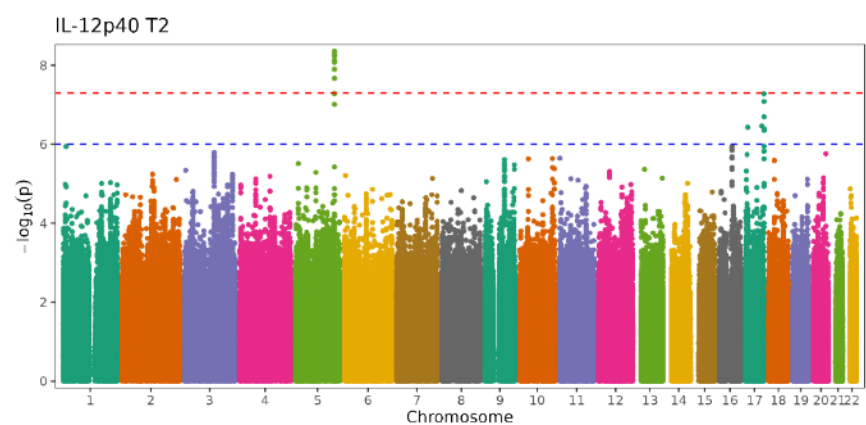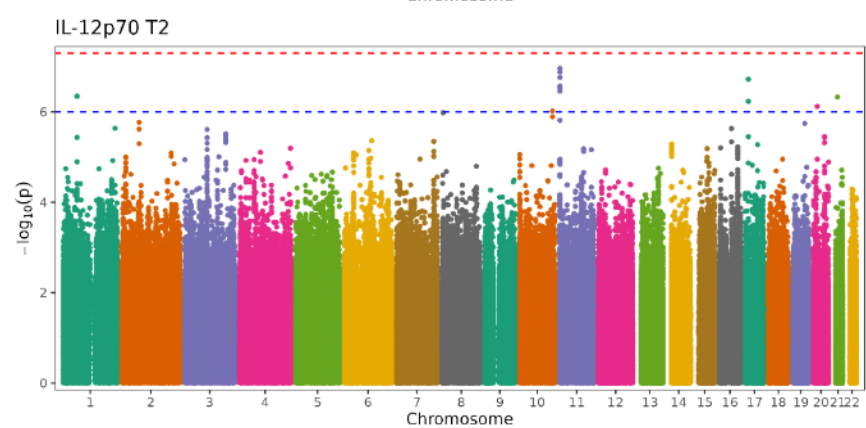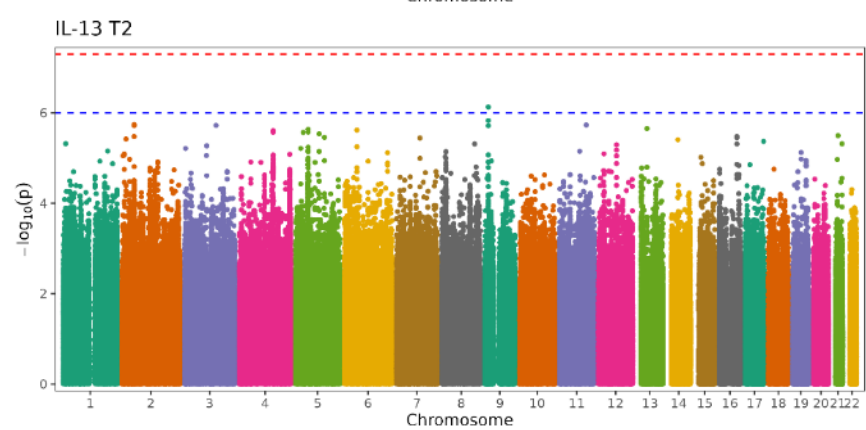

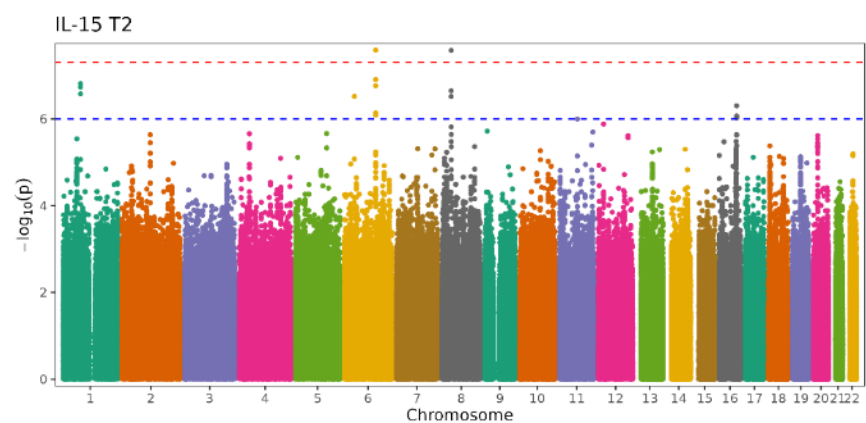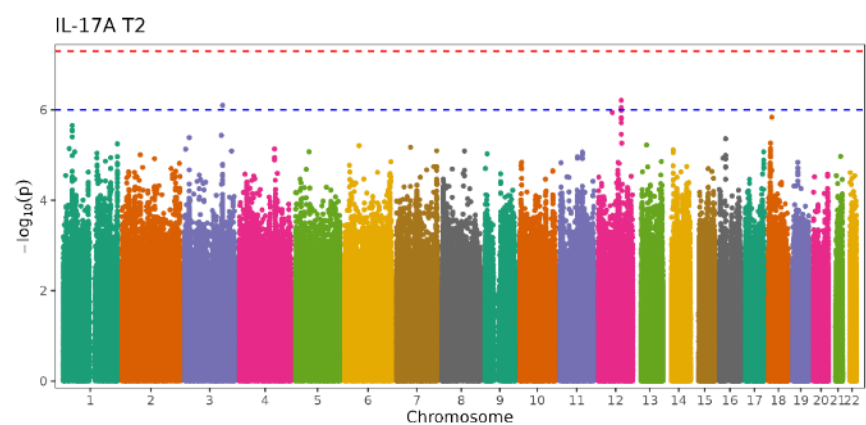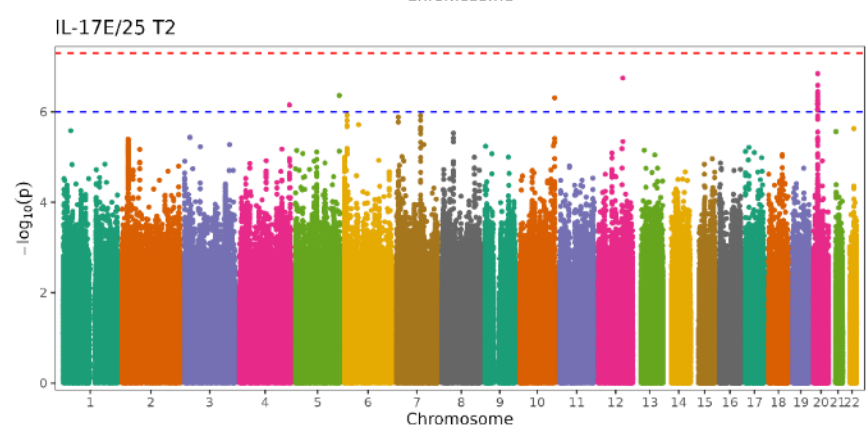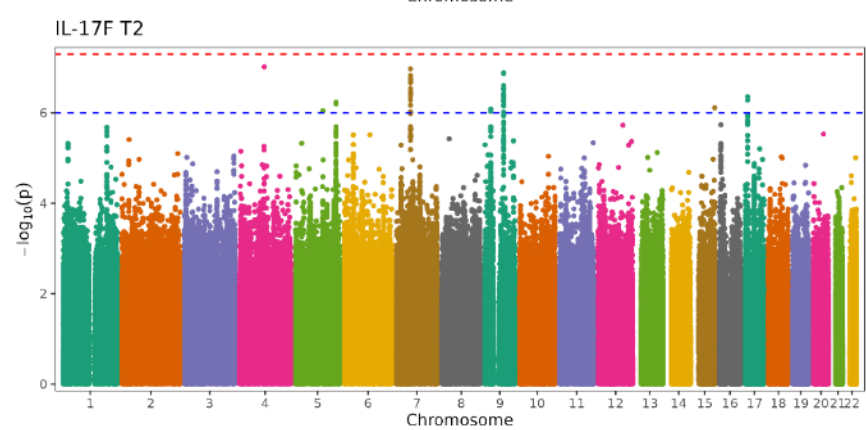

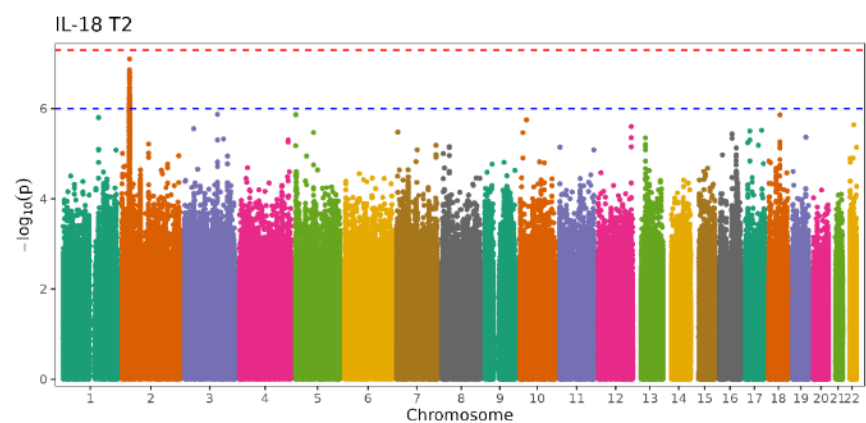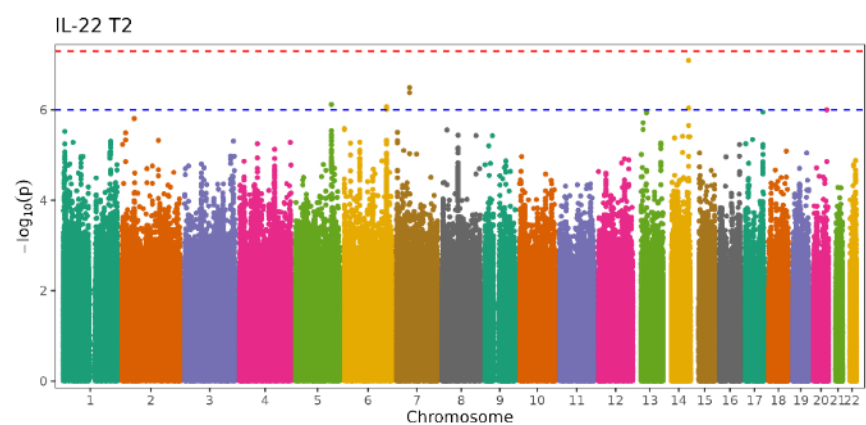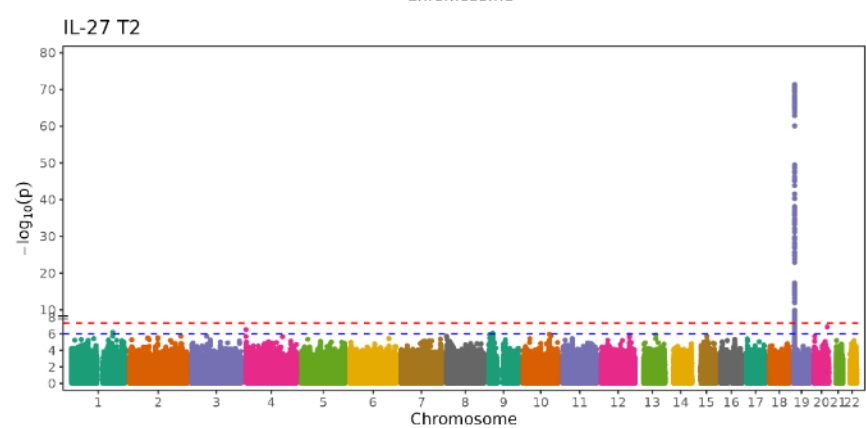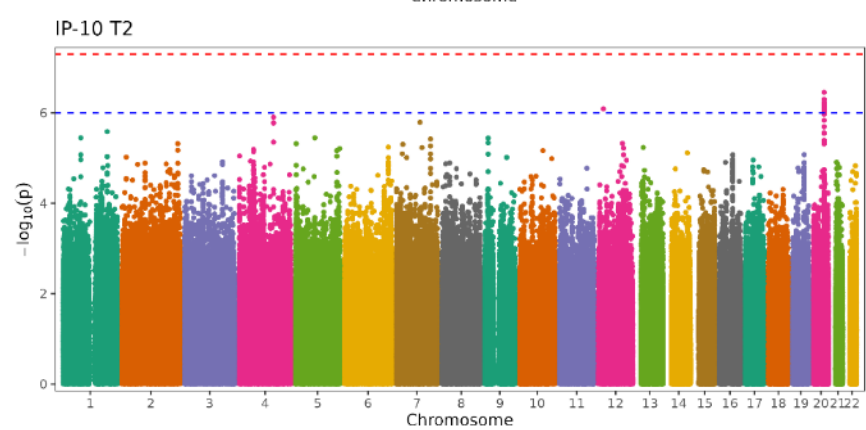

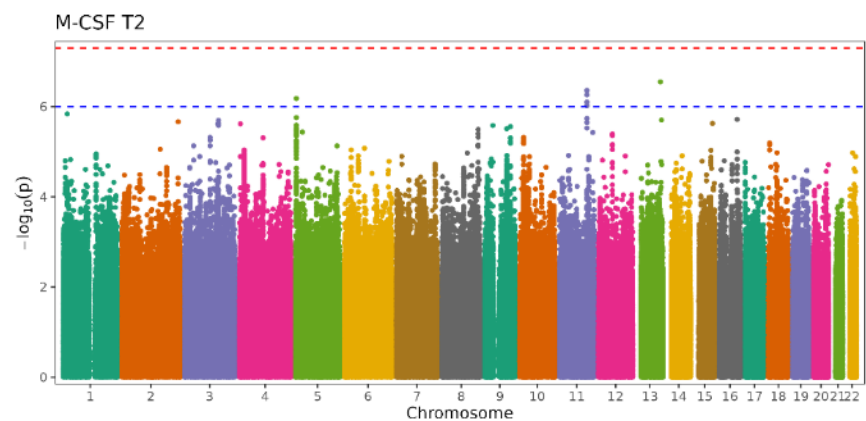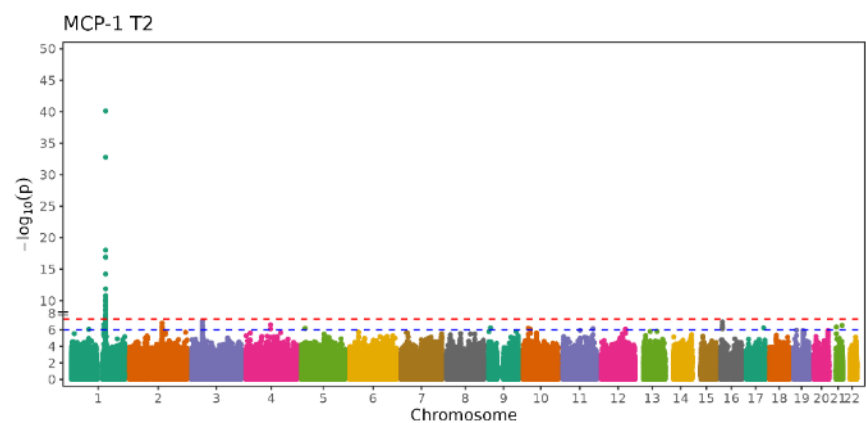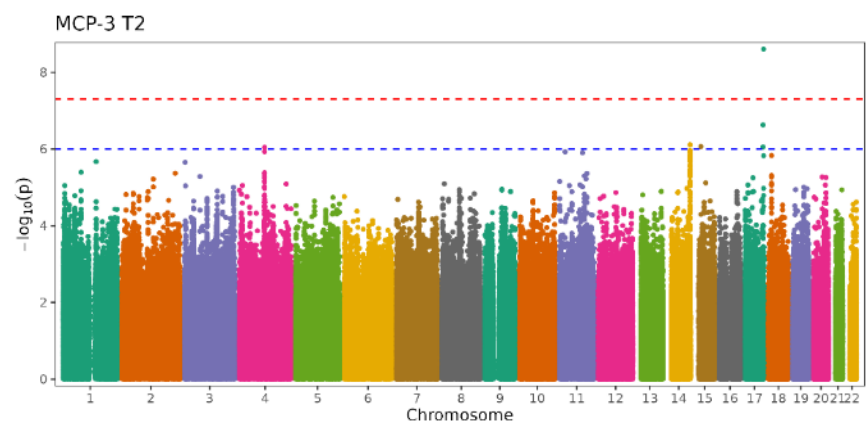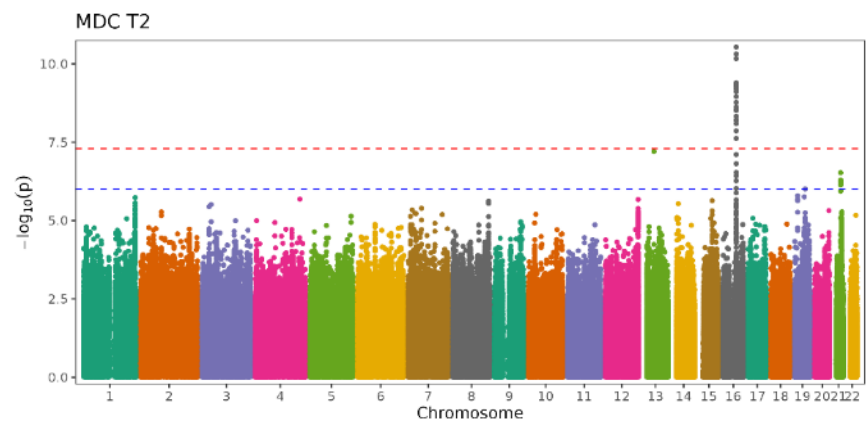

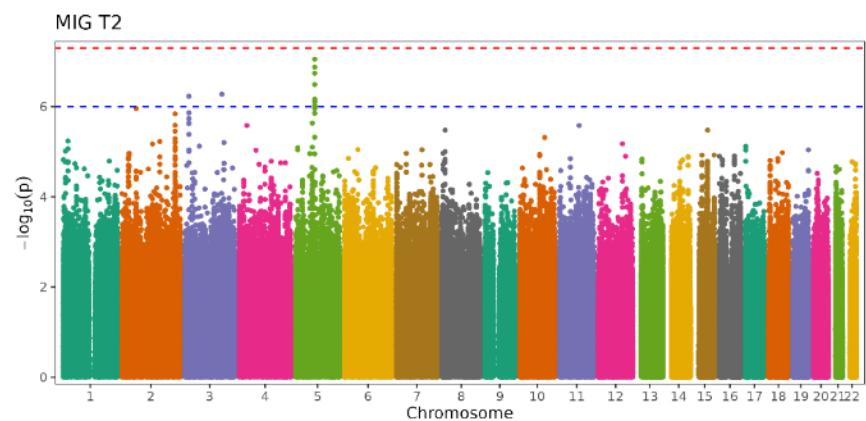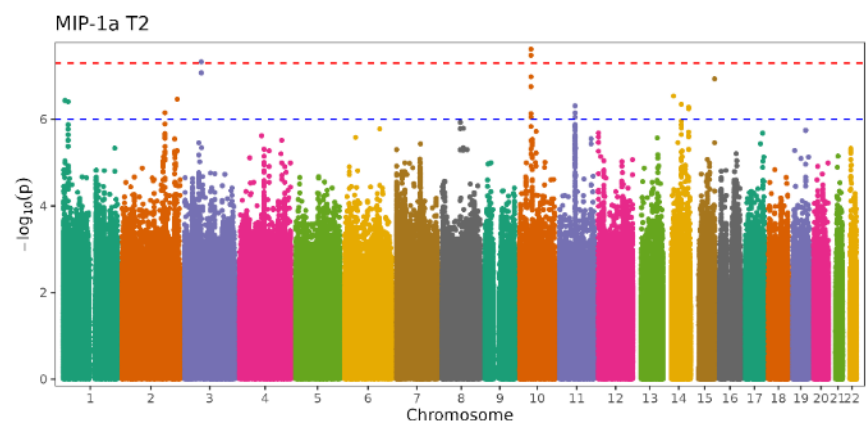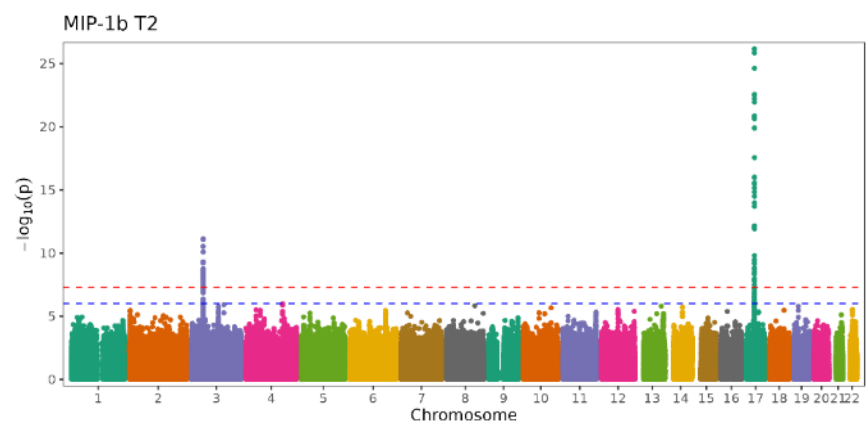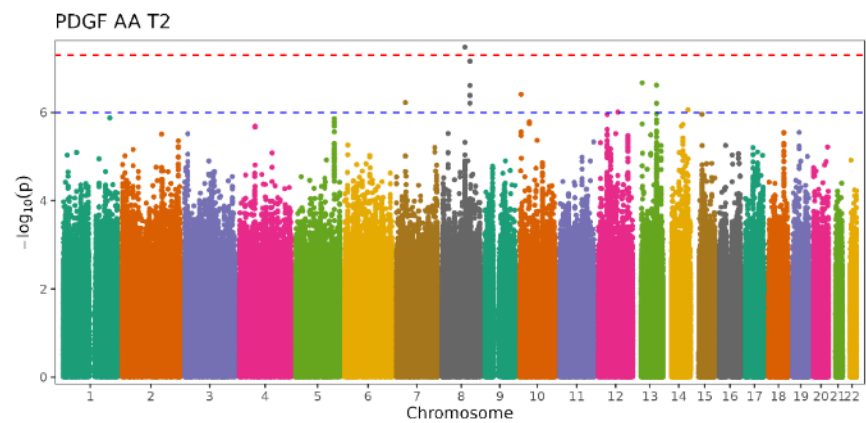

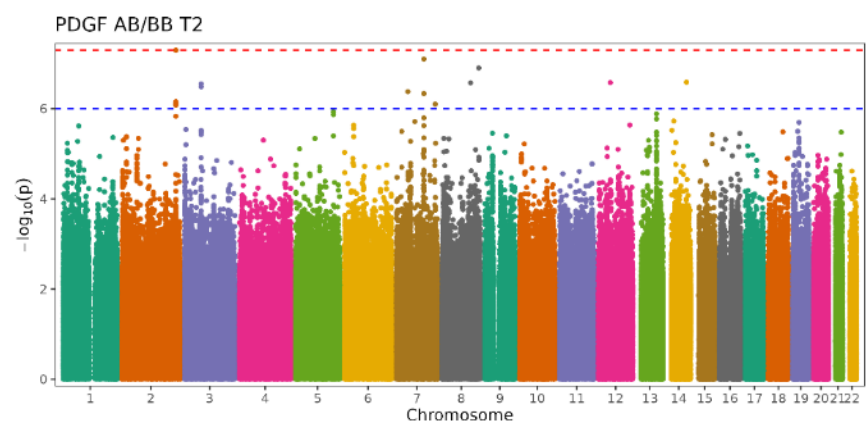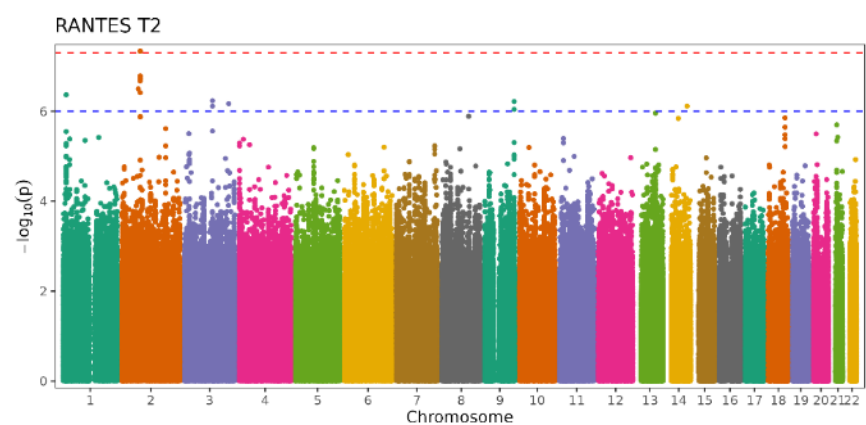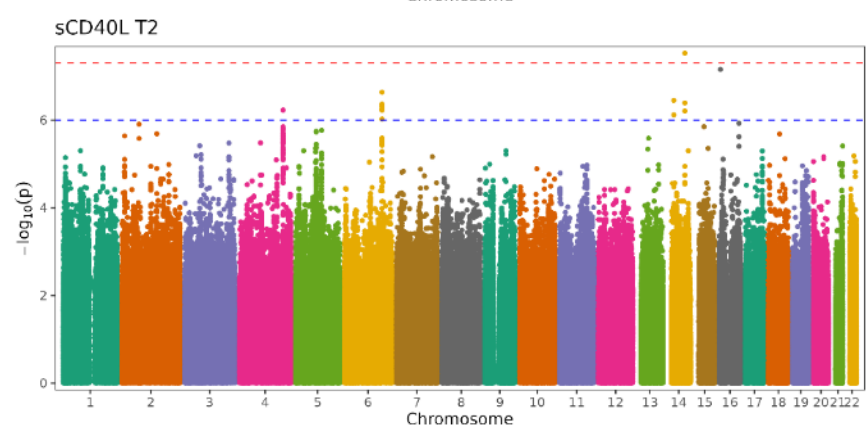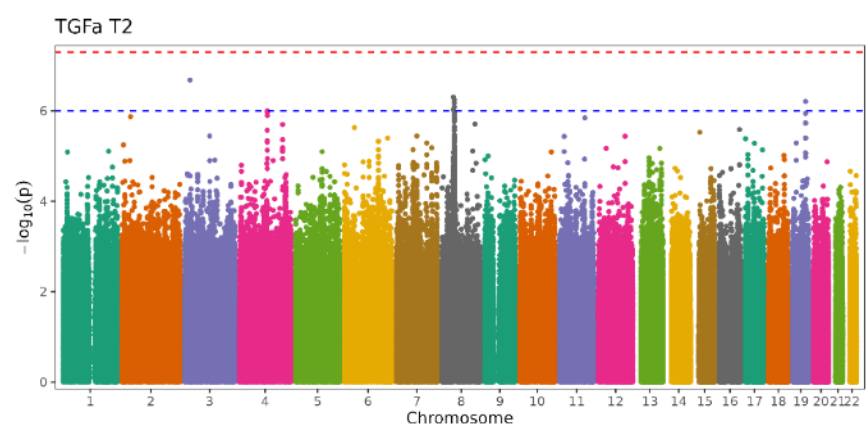

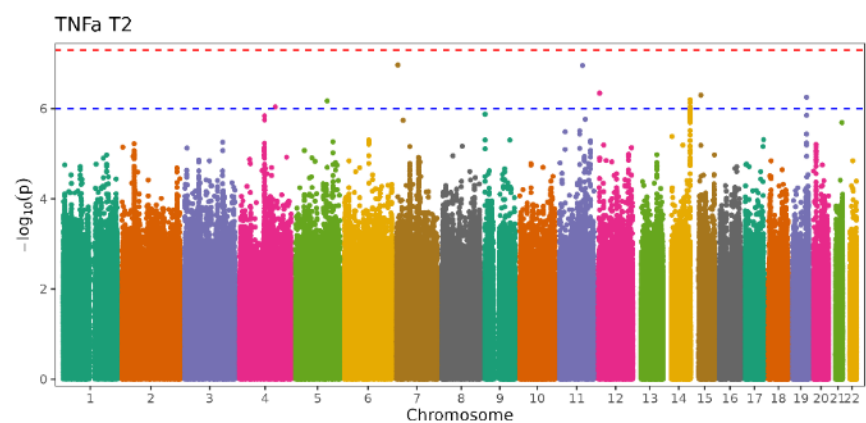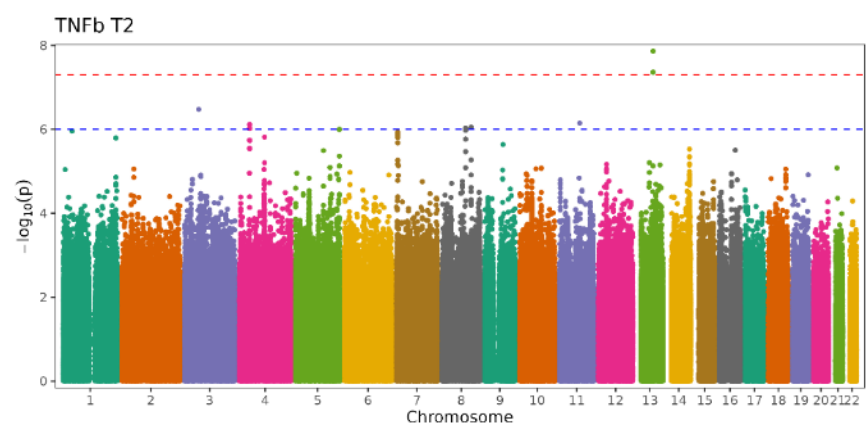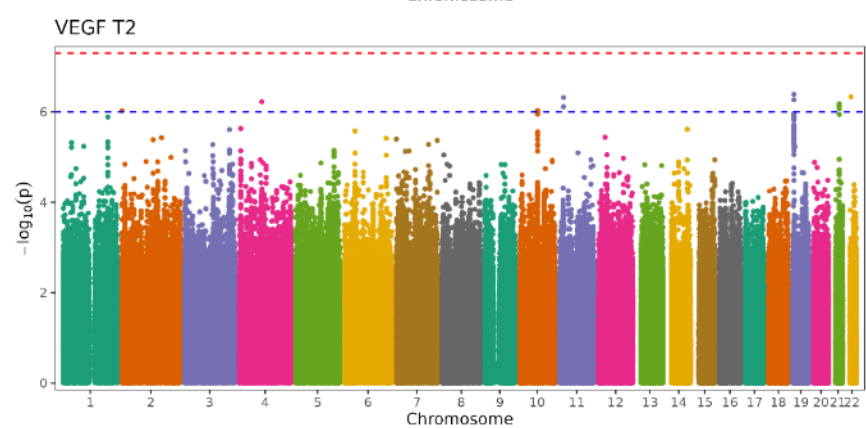

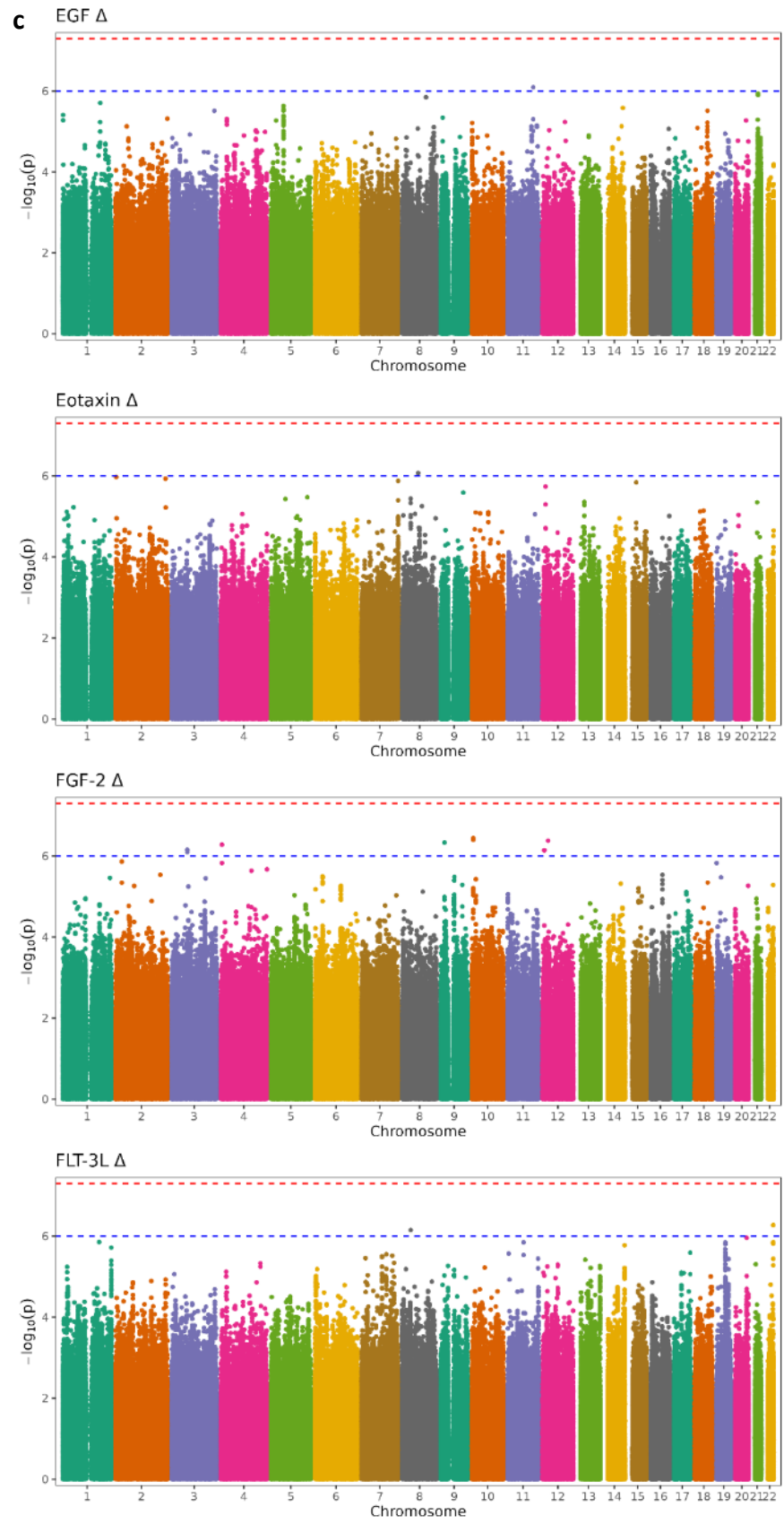

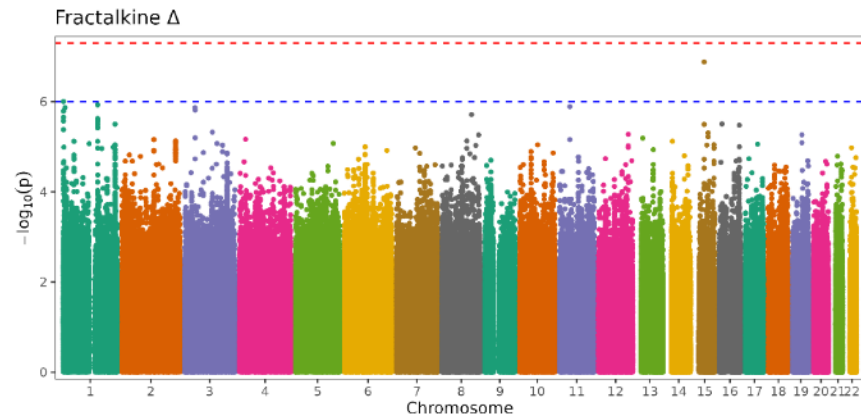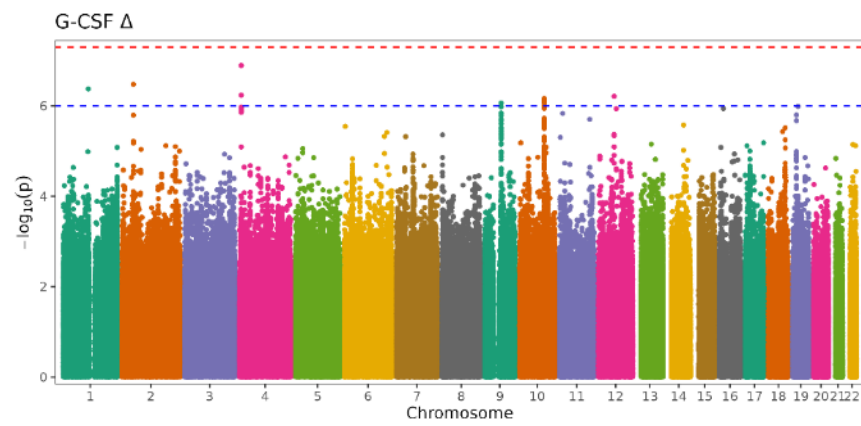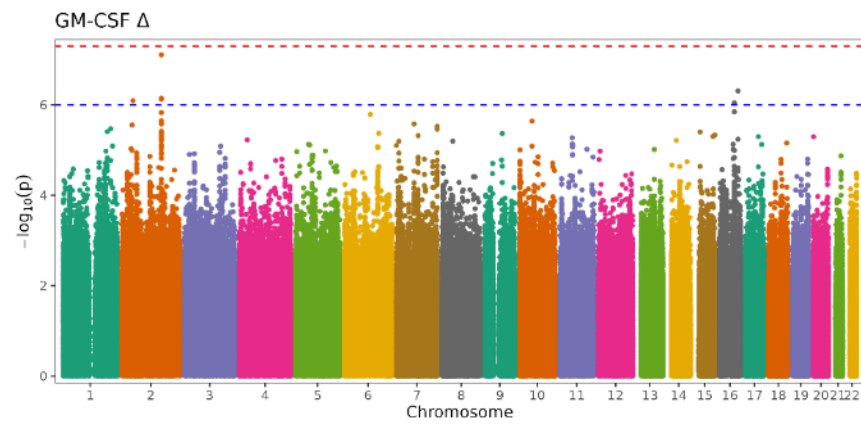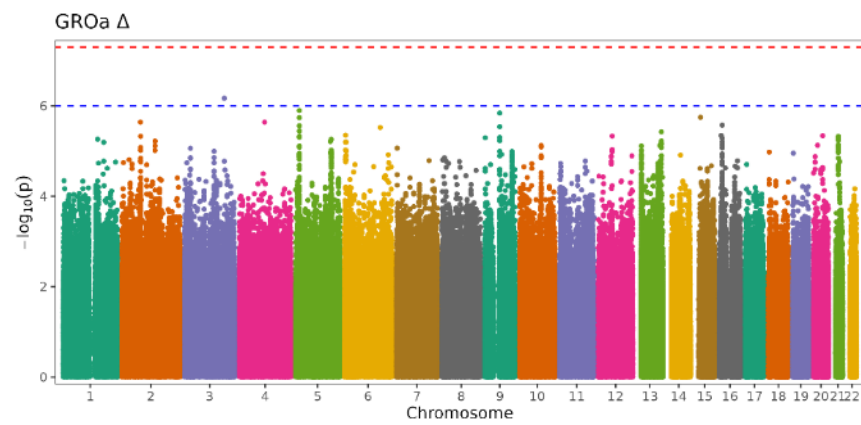

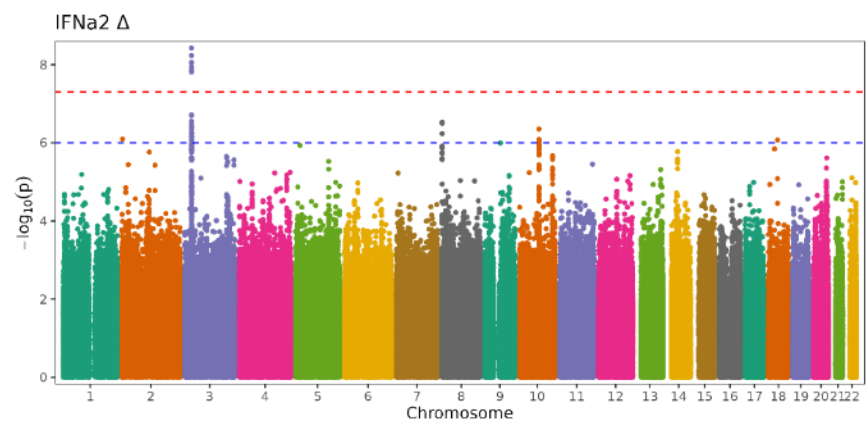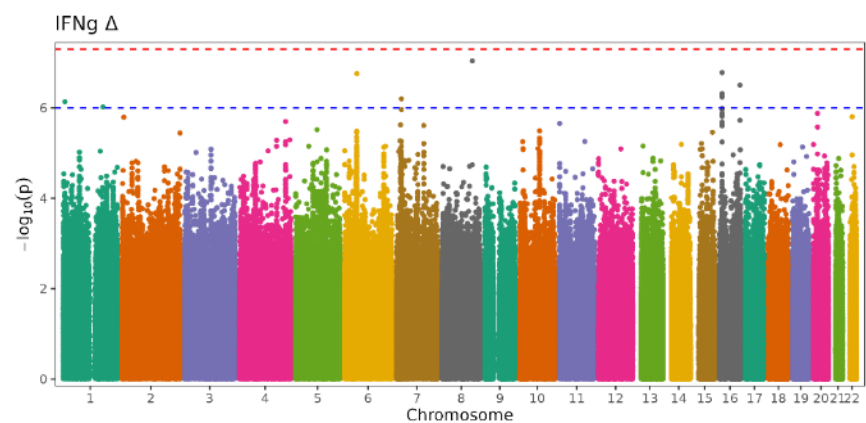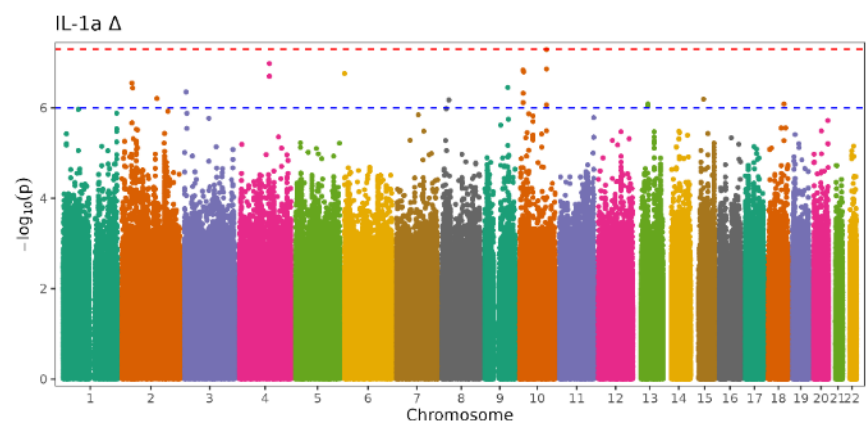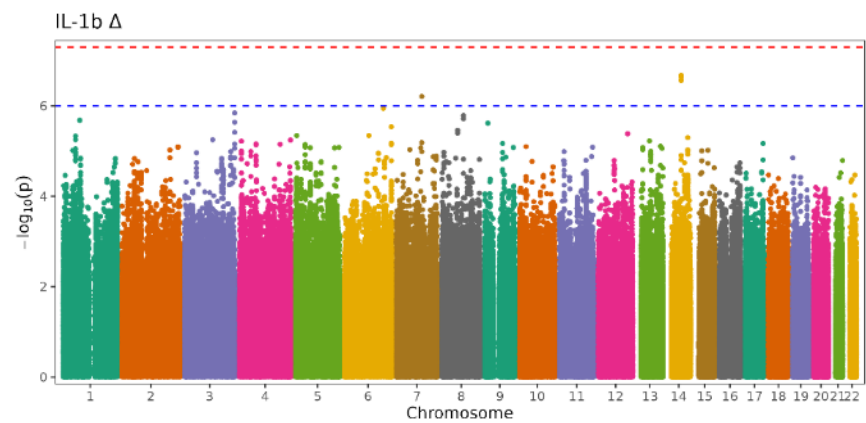

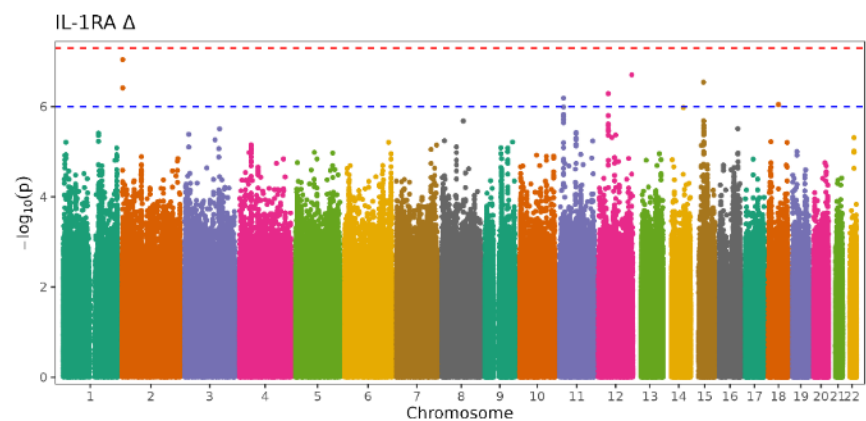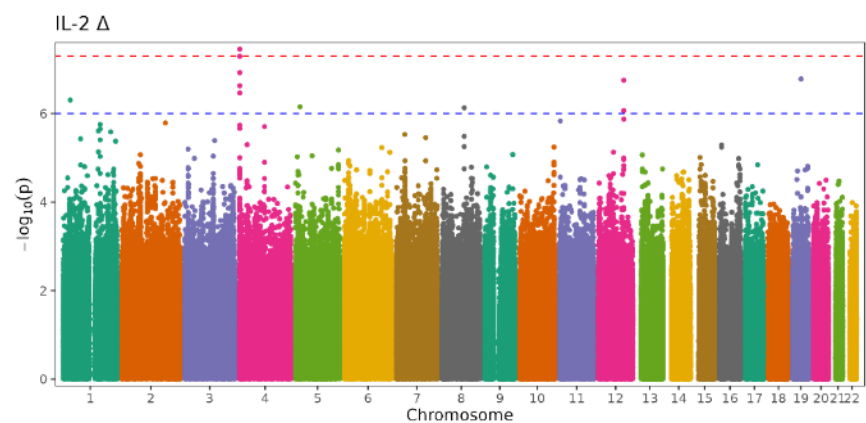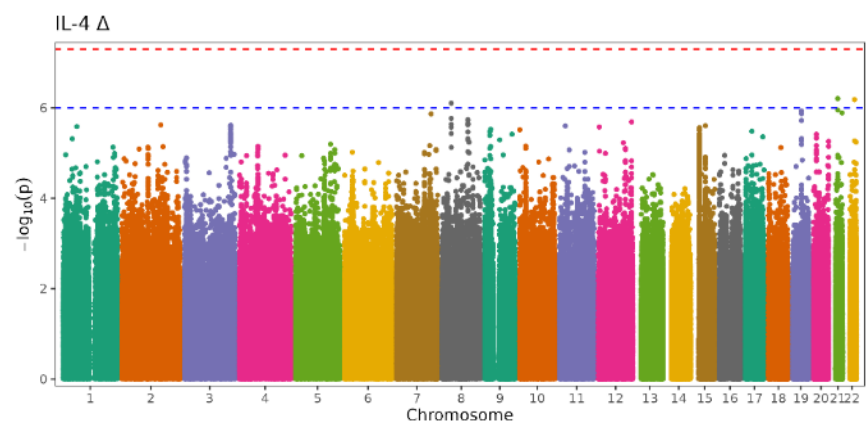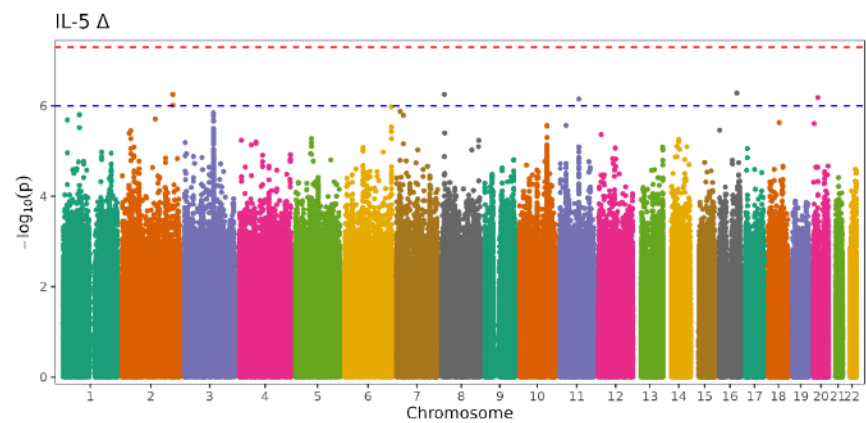

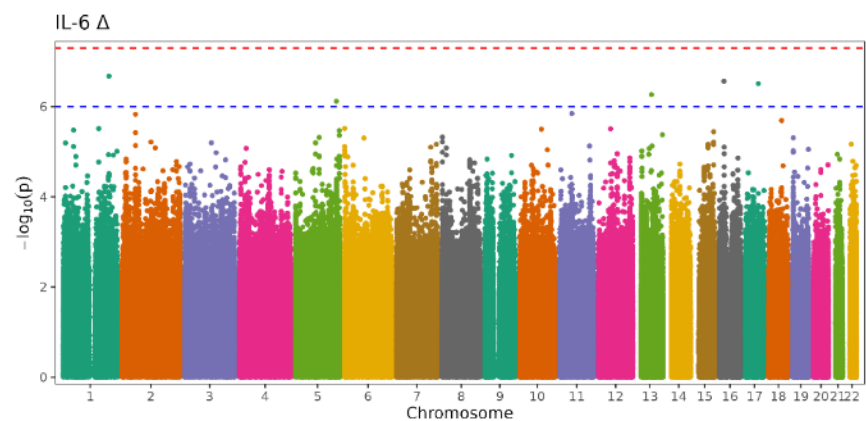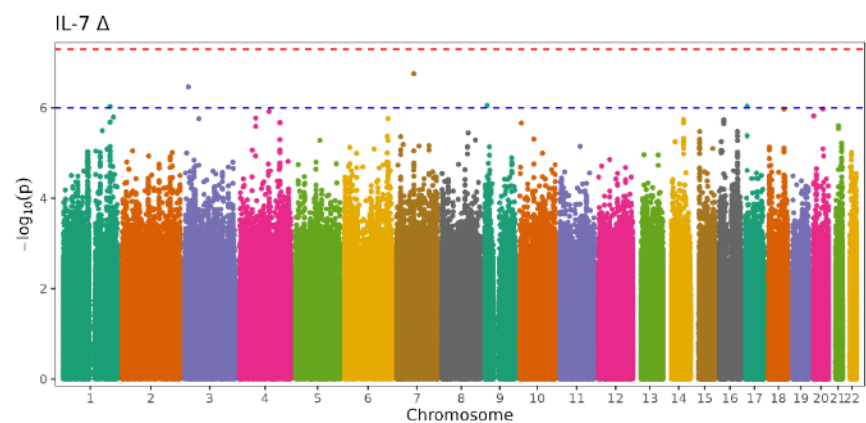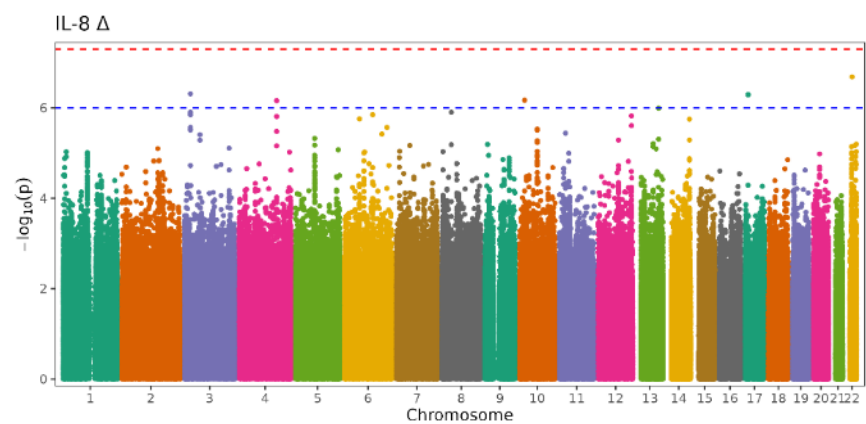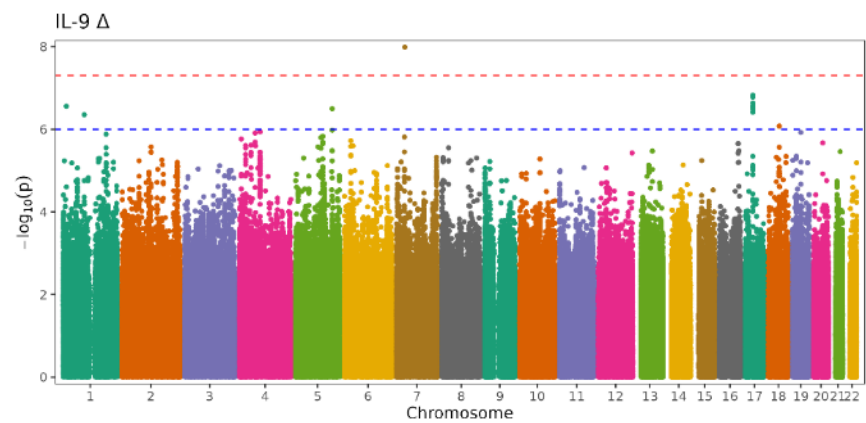

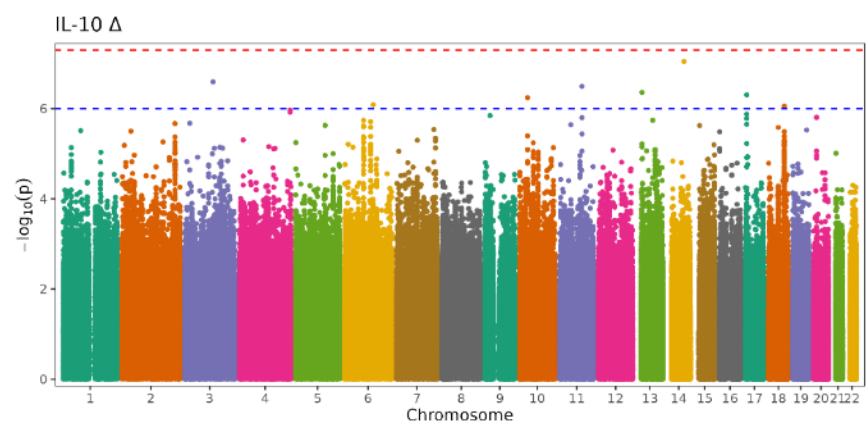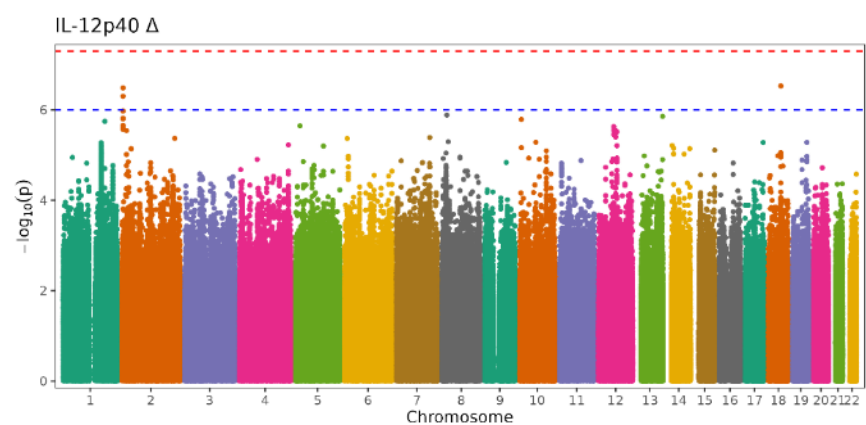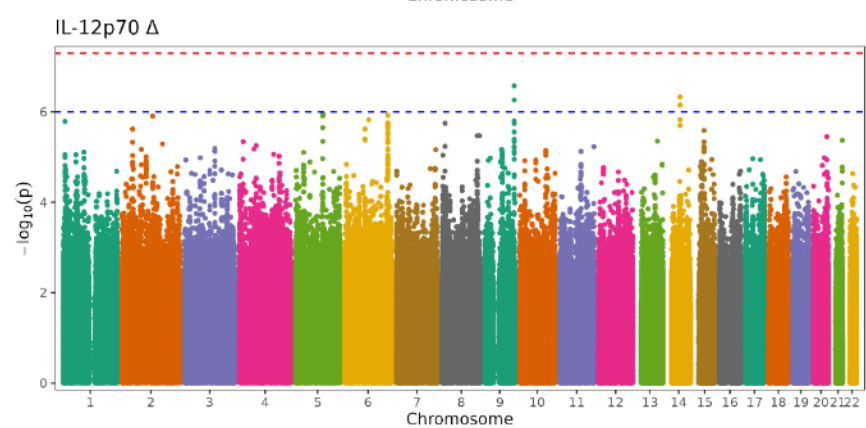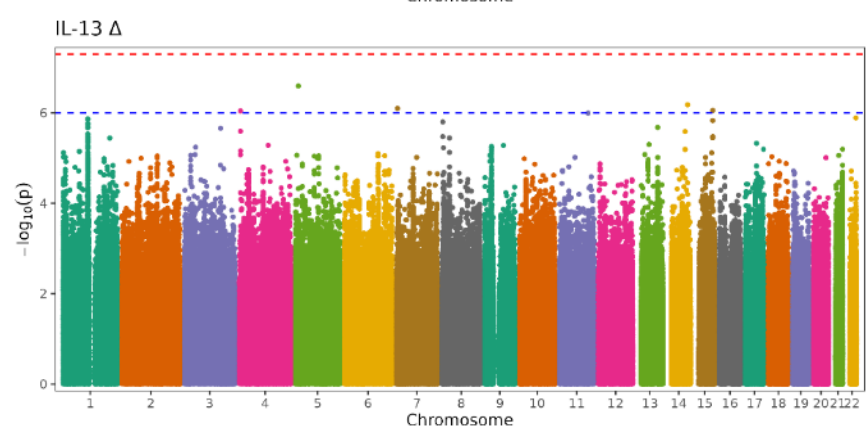

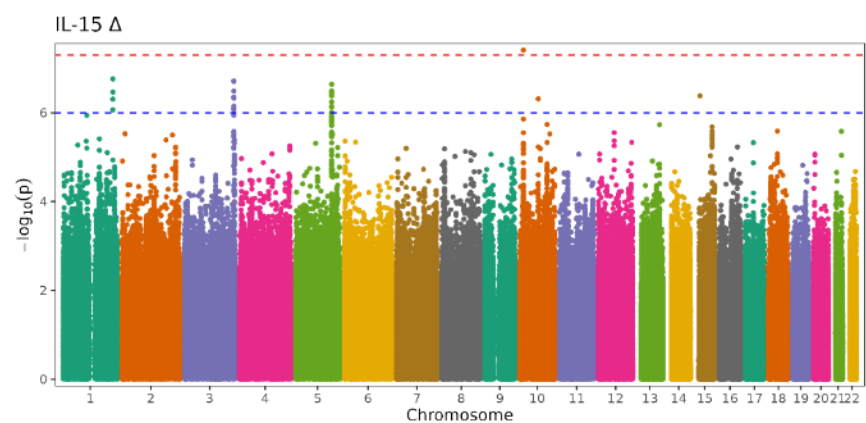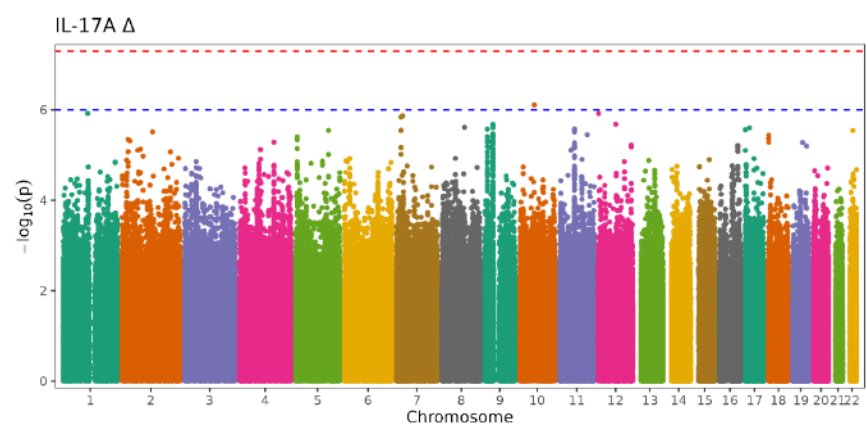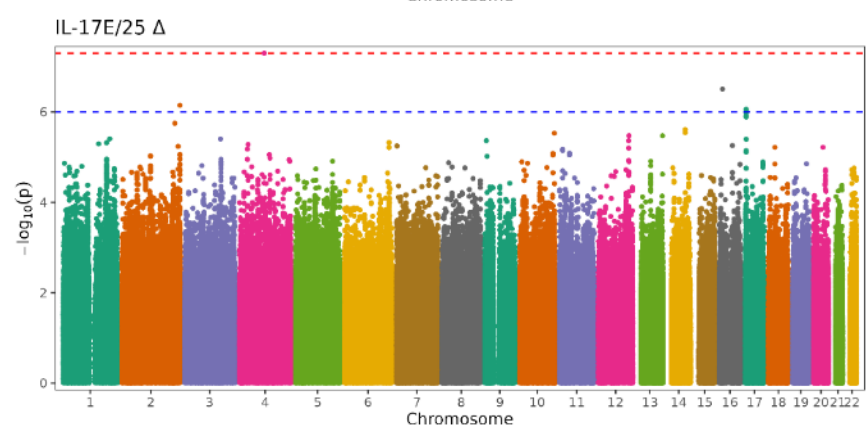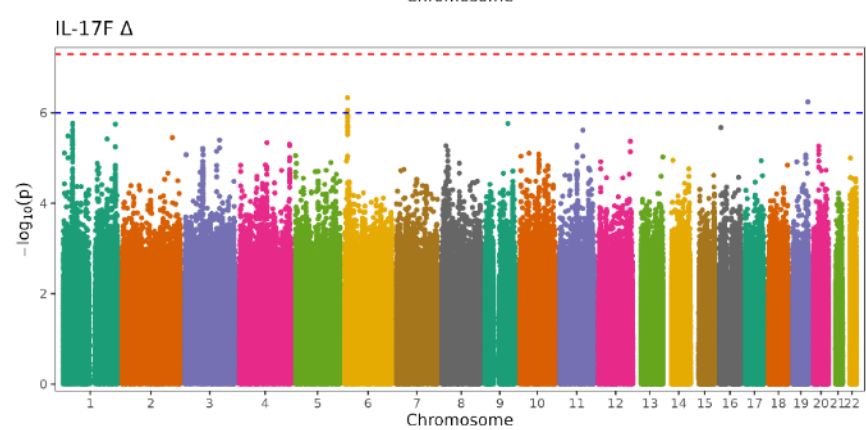

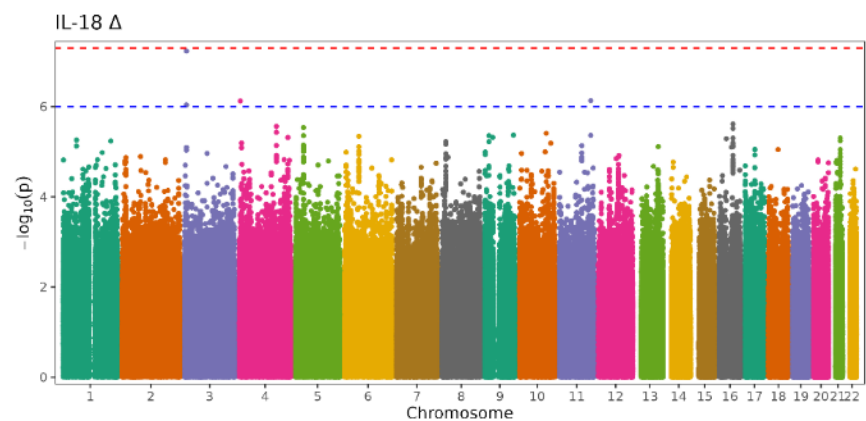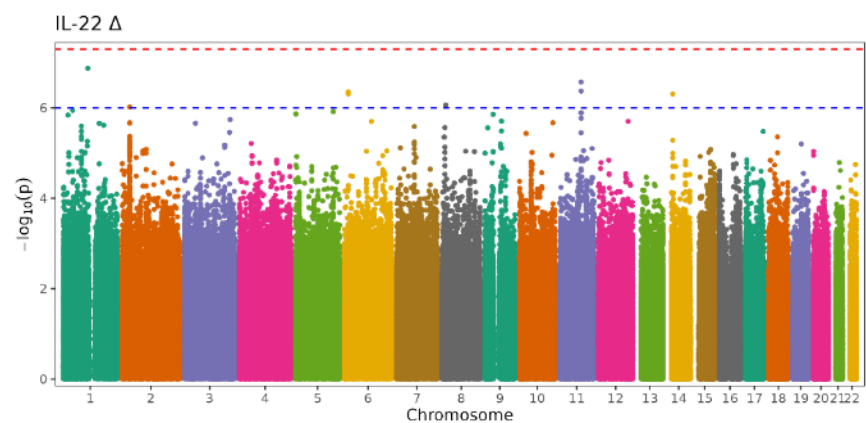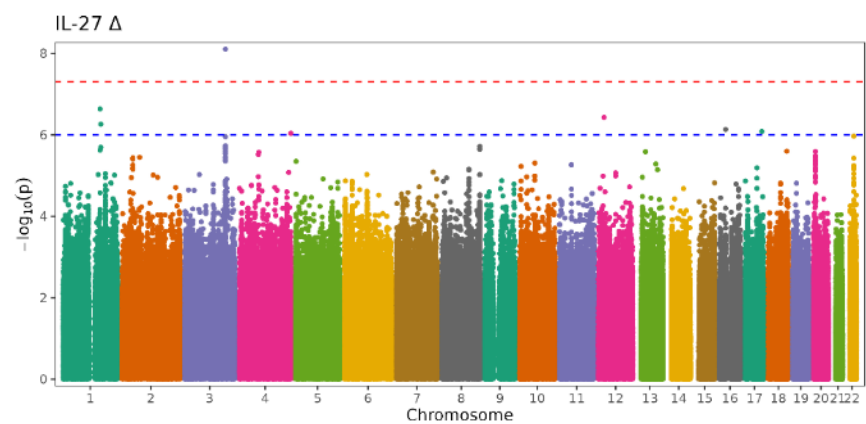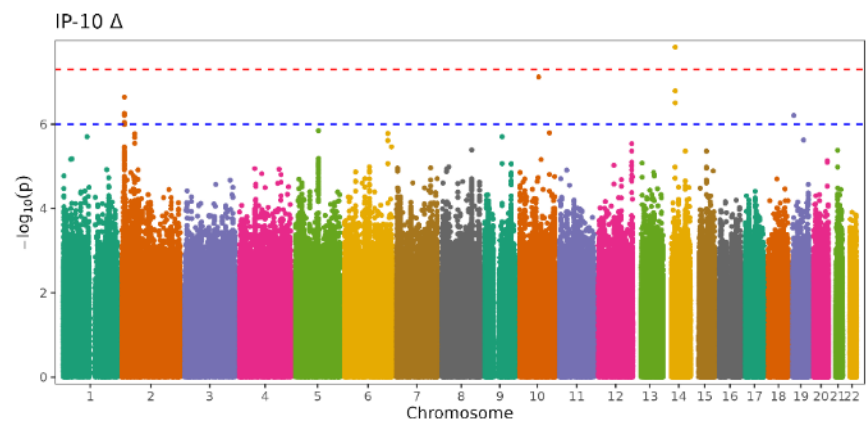

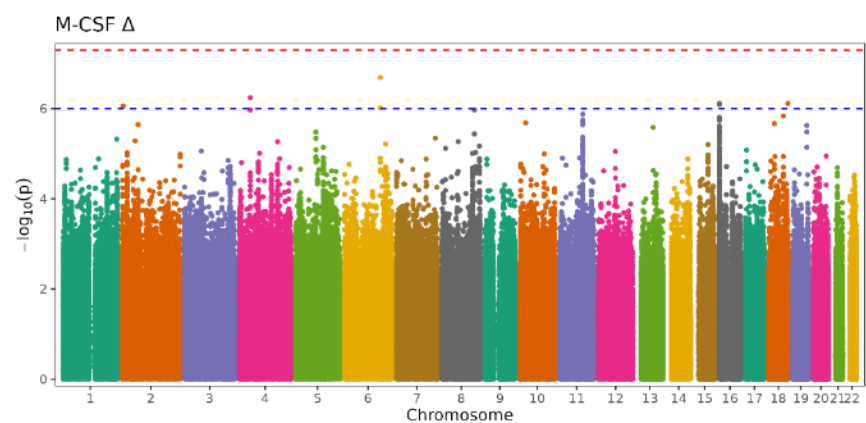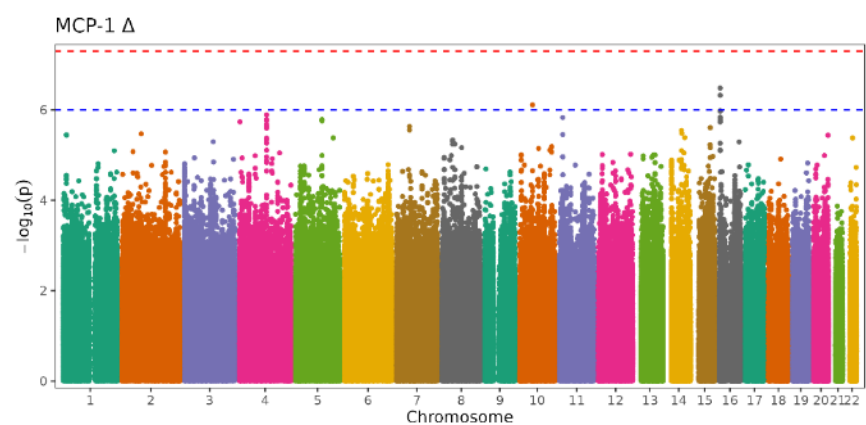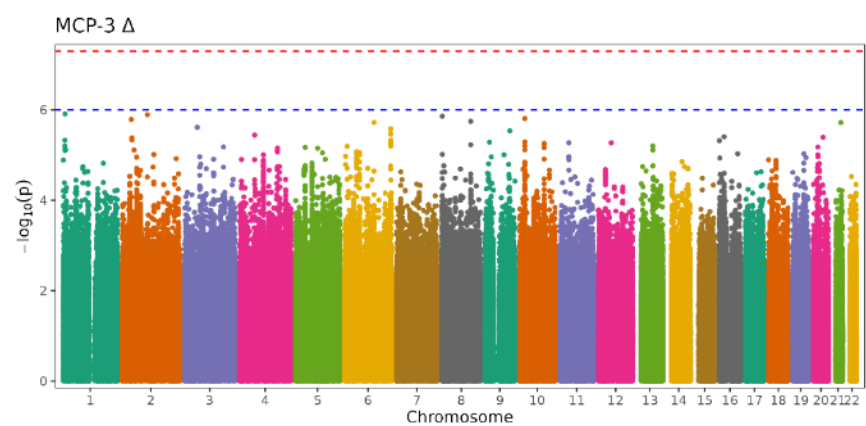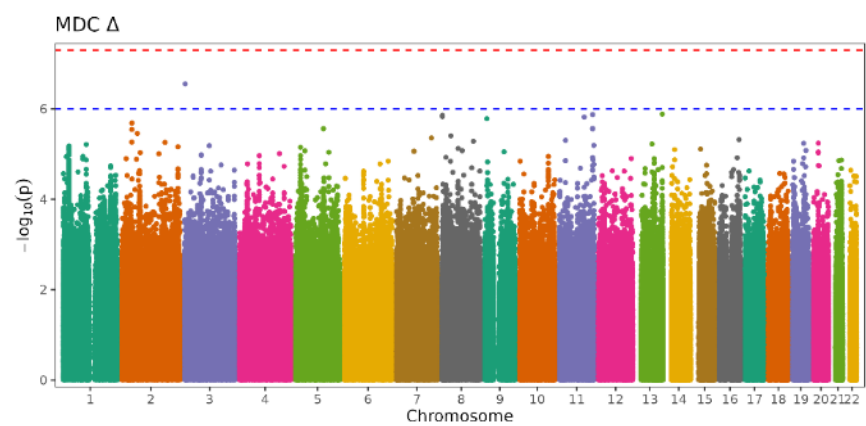

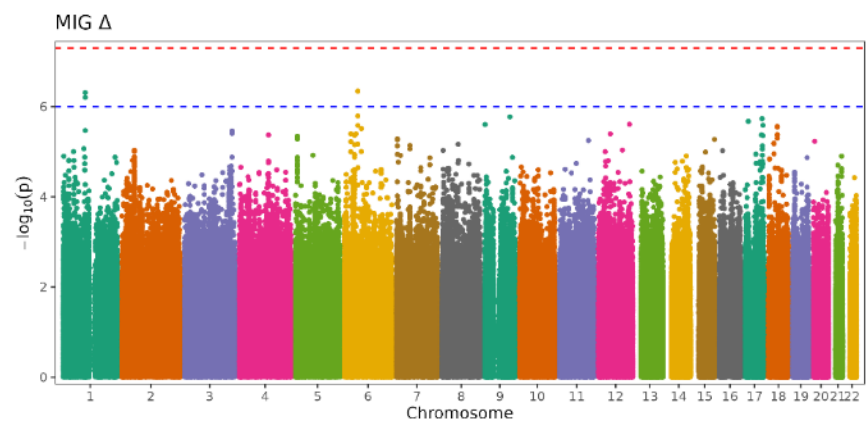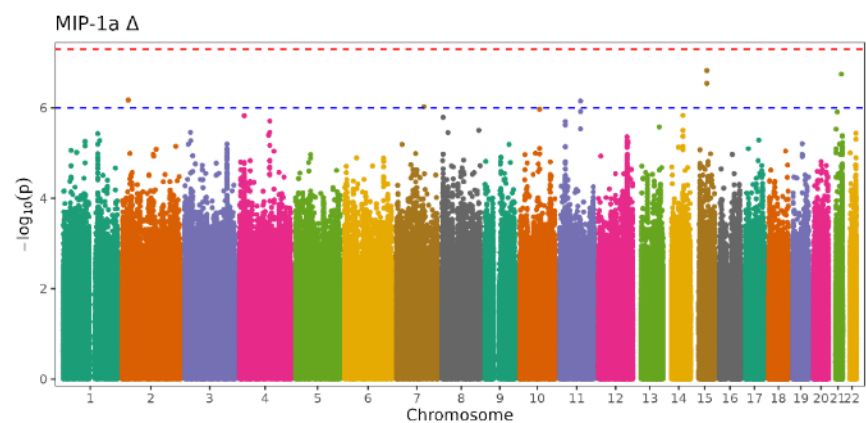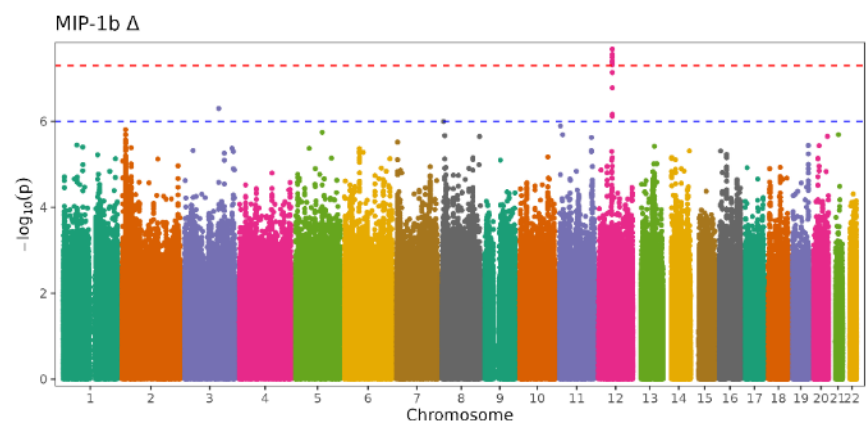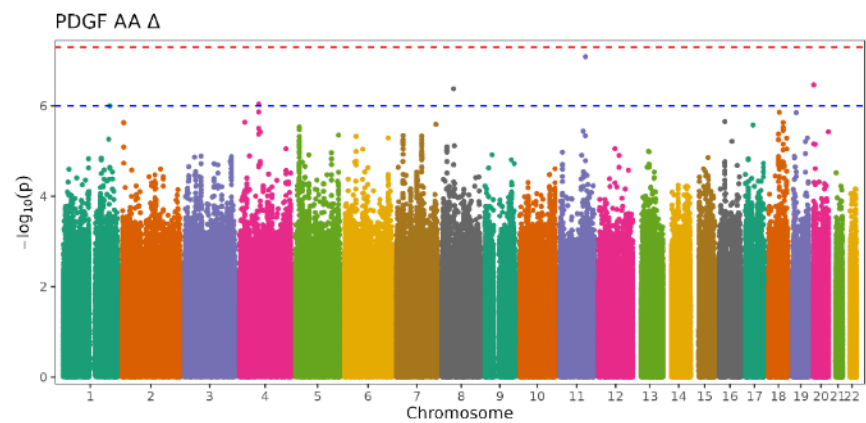

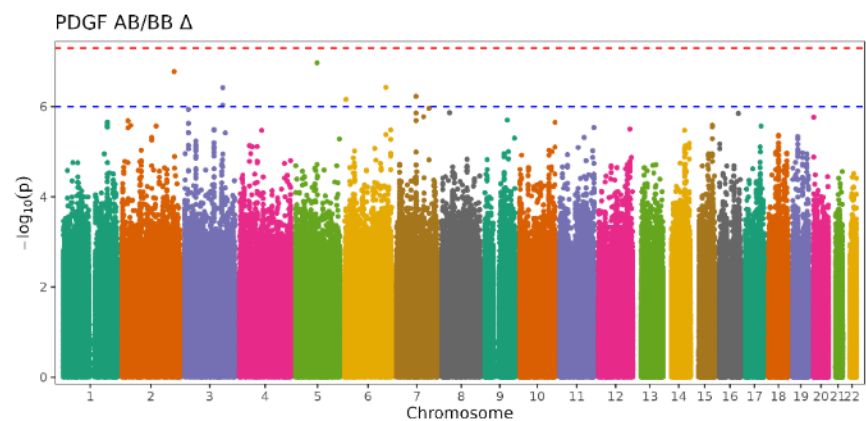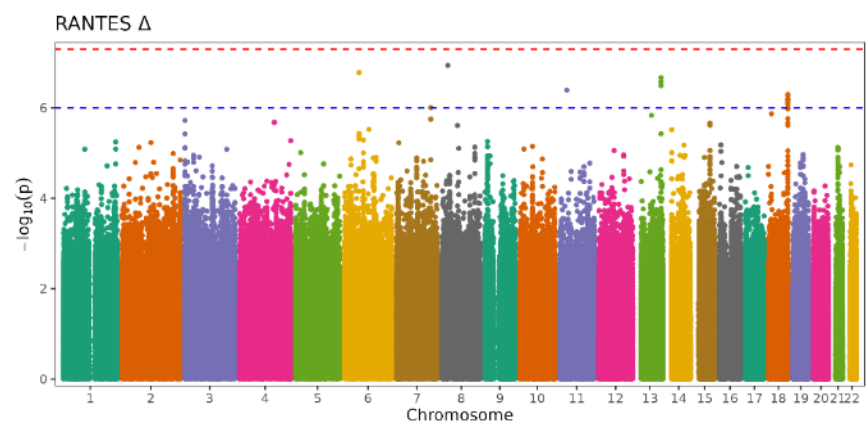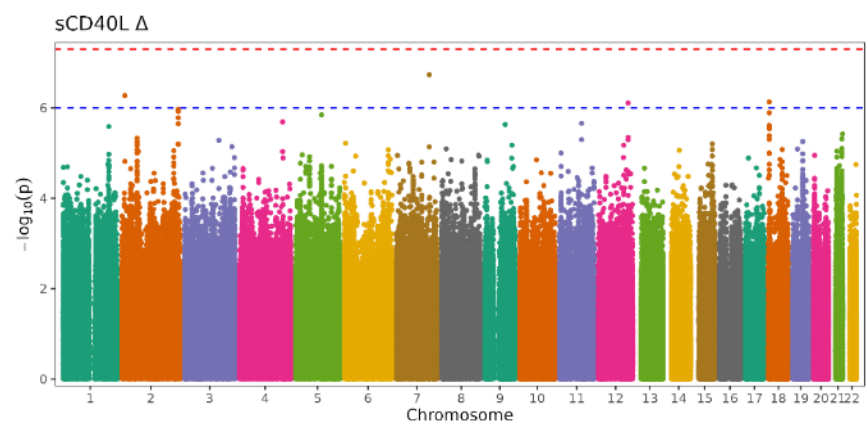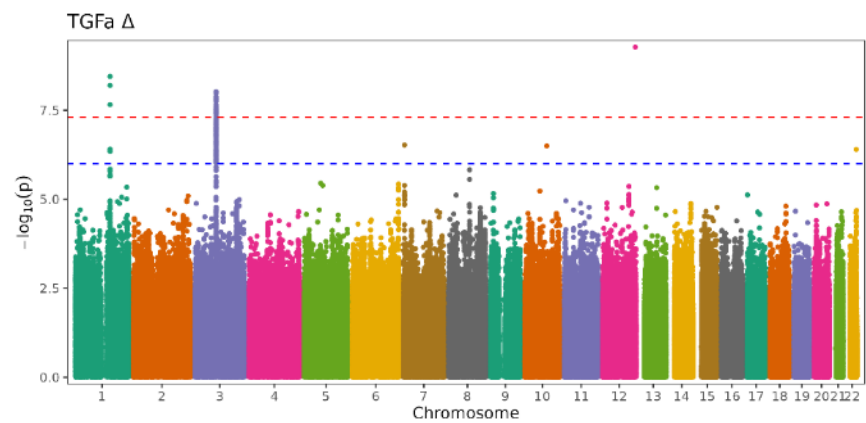

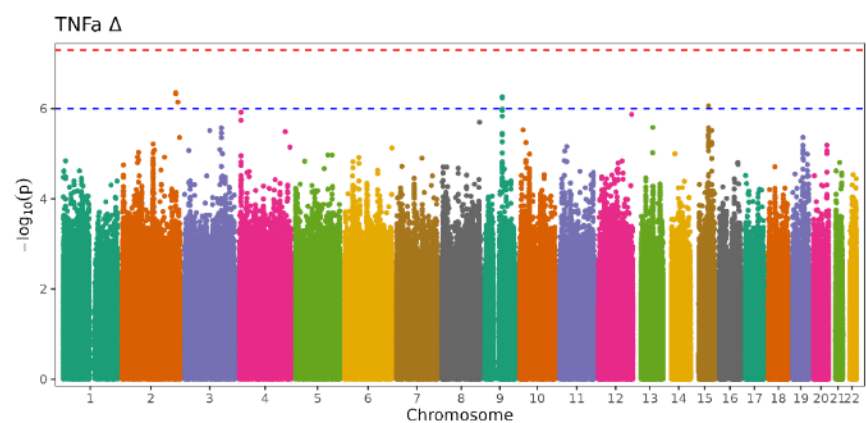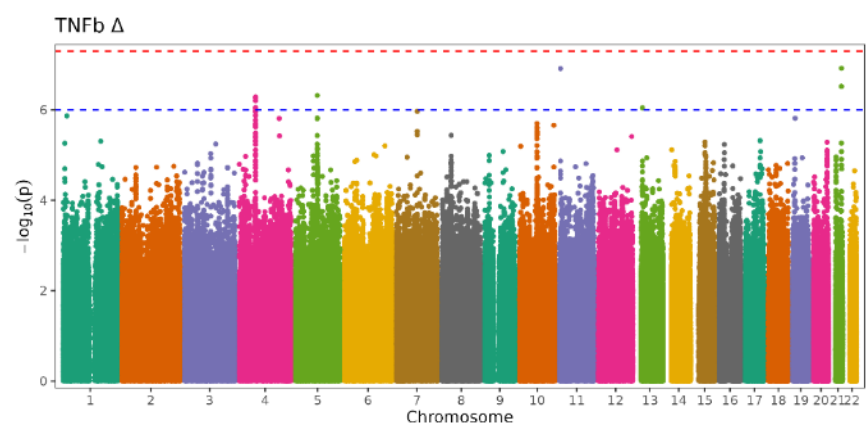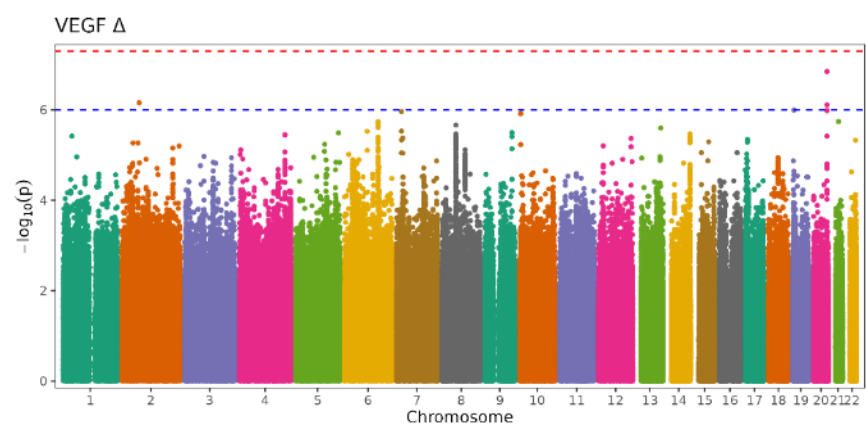

Supplement: S10 Fig — Manhattan plots for each T1 (a), T2 (b), and Δ (c) GWAS. Dashed red line represents P = 5x10^-8^ and dashed blue line represents P = 10-6. (PDF) [file pgen.1012204.s012.pdf]
